# Taxonomic revision of Telemidae (Arachnida, Araneae) from East and Southeast Asia

**DOI:** 10.3897/zookeys.933.38653

**Published:** 2020-05-18

**Authors:** Huifeng Zhao, Shuqiang Li, Aibing Zhang

**Affiliations:** 1 Hebei Key Laboratory of Animal Diversity, College of Life Science, Langfang Normal University, Langfang 065000, China Capital Normal University Beijing China; 2 College of Life Sciences, Capital Normal University, Beijing 100048, China Langfang Normal University Langfang China; 3 Institute of Zoology, Chinese Academy of Sciences, Beijing 100101, China Institute of Zoology, Chinese Academy of Sciences Beijing China

**Keywords:** Haplogynae, molecular phylogeny, new genus, new species, new combination

## Abstract

Species of the spider family Telemidae Fage, 1913 from East and Southeast Asia are revised. Four new genera are erected: *Mekonglema* Zhao & Li, **gen. nov.** with the type species *Mekonglema bailang* Zhao & Li, **sp. nov.** (♂♀, Yunnan, China), *Siamlema* Zhao & Li, **gen. nov.** with the type species *Siamlema changhai* Zhao & Li, **sp. nov.** (♂♀, southern Thailand), *Sundalema* Zhao & Li, **gen. nov.** with the type species *Sundalema bonjol* Zhao & Li, **sp. nov.** (♂♀, Sumatra), and *Zhuanlema* Zhao & Li, **gen. nov.** with the type species *Zhuanlema peteri* Zhao & Li, **sp. nov.** (♂♀, northern Laos). Eight additional new species are described: *Mekonglema kaorao* Zhao & Li, **sp. nov.** (♂♀, northern Laos), *M. walayaku* Zhao & Li, **sp. nov.** (♂♀, Yunnan, China), *M. yan* Zhao & Li, **sp. nov.** (♂♀, Yunnan, China), *Pinelema daguaiwan* Zhao & Li, **sp. nov.** (♂♀, Guangxi, China), *P. shiba* Zhao & Li, **sp. nov.** (♂♀, Guangxi, China), *P. tham* Zhao & Li, **sp. nov.** (♂♀, northern Laos), *Siamlema suea* Zhao & Li, **sp. nov.** (♂♀, southern Thailand), and *Sundalema khaorakkiat* Zhao & Li, **sp. nov.** (♂♀, southern Thailand). Thirty species are transferred from the genus *Telema* Simon, 1882 to the genera *Pinelema* Wang & Li, 2012, *Sundalema***gen. nov.**, and *Telemofila* Wunderlich, 1995. *Seychellia xinpingi* Lin & Li, 2008 is transferred to *Mekonglema***gen. nov.** as *M. xinpingi***comb. nov.** Furthermore, the genus *Pinelema* is divided into seven species groups based on male morphological characters.

## Introduction

The spider family Telemidae Fage, 1913 currently includes 85 species in ten genera (Li 2020). Telemids are tiny spiders, whose body length ranges from 0.9 to 2.2 mm, with much longer legs relative to their body. The dispersal ability of telemids is poor, resulting in high endemism (Lin and Li 2010; Wang and Li 2010b; Zhao et al. 2018a, b).

Telemids occur in tropical rainforests and karst caves in the southern Holarctic, Ethiopian, Oriental, Neotropical, and Australasian Realms (except Australia and New Zealand) (WSC 2020). *Telema tenella* Simon, 1882 occurs in Spain and France and is the type species of the genus *Telema* Simon, 1882 and the only known telemid species from Europe. Thirty-four Asian species were classified in the poorly defined genus *Telema* prior to the current study, and a revision of Asian *Telema* is necessary. Furthermore, the placement of *Seychellia xinpingi* Lin & Li, 2008 from southwestern China requires examination because the shape of the bulbal apophysis of the male differs from the generotype *Seychellia wiljoi* Saaristo, 1978, from Seychelles.

The goal of this paper is to revise all Asian telemid species using combined morphological and molecular approaches, adding new materials collected from Southeast Asia and southwestern China.

## Materials and methods

Specimens used in this paper were collected by sifting leaf litter in rainforests or collected by hand from caves. All samples were examined and measured using a LEICA M205C stereomicroscope. The habitus, left male palp, and endogyne were photographed using an Olympus C7070 wide zoom digital camera. Images were montaged using Helicon Focus Lite 7.5.6 software. Female genitalia were removed and treated in lactic acid before being photographed. All measurements are given in millimetres. Leg measurements are shown as: total length (femur, patella, tibia, metatarsus, tarsus). For SEM images, the tibial glands on leg III were photographed using a Hitachi SU8010 Environmental Scanning Electron Microscope.

For molecular phylogenetic analyses, we used all available materials of Asian telemids as well as the type species *Telema tenella* and *Seychellia wiljoi*. Our analyses contain 57 of 62 known Asian species from the type localities and 12 potentially new species. Five species for which we did not obtain molecular data are *Sundalema acicularis* (Wang & Li, 2010) comb. nov., *Pinelema claviformis* (Tong & Li, 2008) comb. nov., *Telemofila malaysiaensis* (Wang & Li, 2010) comb. nov., *Telema nipponica* (Yaginuma, 1972), and *Pinelema spina* (Tong & Li, 2008) comb. nov. We used two segestriid species as outgroups, as Segestriidae is considered the sister lineage of Telemidae (Shao and Li 2018). In total, 73 taxa were included in our molecular dataset.

Genomic DNA was extracted from the legs or prosoma using TIANamp Genomic DNA Kit DP304 (TIANGEN Co., Beijing, China). Two nuclear loci, Histone 3 and Wingless (H3 and Wnt), were amplified for subsequent molecular phylogenetic analyses. Primer information and PCR protocols are shown in Suppl. material 1: Table S1. All amplicons were sequenced using an ABI 3730 automated sequencer, and raw sequences were corrected manually in BioEdit (Hall 1999). Sequence alignments were produced using Clustal W in MEGA 5 (Tamura et al. 2011), and the sequences were checked for stop codons after translation to amino acid sequences.

Phylogenetic relationships were inferred using maximum likelihood (ML) and Bayesian inference (BI). ML was performed in RAxML 7.0.4 (Stamatakis 2014) using the default rapid hill-climbing algorithm and the GTRGAMMA model to search for the best tree. Clade support was assessed using 1000 rapid bootstrap replicates. BI was performed in MrBayes 3.2.6 (Ronquist et al. 2012) using the best model selected by jModeltest 2.1.7 (Darriba et al. 2012) based on the Akaike information criterion (AIC). BI analysis was run for 10 million generations, sampling every 1000 generations, and was checked to ensure the average standard deviation of split frequencies was less than 0.01.

References to figures from the cited papers are listed in lowercase (fig. or figs), and figures in this paper are noted with an initial capital (Fig. or Figs). The following abbreviations are used:

**Ca** Cymbial apophysis

**Em** Embolus

**BA** Bulbal apophysis

**Re** Receptacle

**SR** Spiral ridge on embolus

Abbreviations of institutes:

**AMNH** American Museum of Natural History, New York, USA

**IZCAS** Institute of Zoology, Chinese Academy of Sciences, Beijing, China

**MHBU** Museum of Hebei University, Baoding, Hebei, China

**MLR** Maolan National Natural Reserve, Libo, Guizhou, China

**RMNH** National Museum of Natural History, Leiden, the Netherlands

**SMF** Senckenberg Research Institute in Frankfurt, Germany

Types are deposited in IZCAS, except *Mekonglema kaorao* sp. nov. and *Zhuanlema peteri* sp. nov., which are lodged in SMF.

## Taxonomy

**Family Telemidae Fage, 1913**

### Key to telemid genera occurring in East and Southeast Asia (Males)

(*Apneumonella* Fage, 1921 was excluded as the male of *A. jacobsoni* Brignoli, 1977 is unknown).

**Table:** 

1	Tibial glands plate-shaped (Fig. 1A; Emerit 1984: fig. A), cymbial apophysis absent (Wang et al. 2012: fig. 2C)	***Telema* Simon, 1882**
–	Tibial glands belt-shaped (Fig. 1B–G; Emerit 1984: fig. C, D), cymbial apophysis present (Figs 12C, 20C, 25C, 28C; Wang and Li 2012: fig. 2; Wunderlich 1995: fig. 16)	**2**
2	Palpal tibia with a dorso-distal spine, cymbium shorter than femur (Fig. 20C, D)	***Siamlema* gen. nov.**
–	Palpal tibia without dorso-distal spine, cymbium longer than femur	**3**
3	Cymbial apophysis located baso-prolaterally (Fig. 28C), embolus twisted (Fig. 24C, D)	***Zhuanlema* gen. nov.**
–	Cymbial apophysis located sub-baso-, meso-, or sub-disto-prolaterally, embolus not twisted	**4**
4	Bulb spherical, embolus sickle-shaped (Wunderlich 1995: fig. 17), leg formula: 1-4-2-3	***Telemofila* Wunderlich, 1995**
–	Bulb ellipsoidal or nearly ellipsoidal, embolus not sickle-shaped, leg formula: 1-2-4-3	**5**
5	Bulb with one apophysis, and tip of embolus directed ventrally (Fig. 12B–D); or bulb without apophysis, and tip of embolus directed dorsally (Fig. 14C, D)	***Mekonglema* gen. nov.**
–	Bulb without apophysis, and tip of embolus directed ventrally	**6**
6	Embolus with spiral ridge, triangular, trapezoidal, or tube-like	***Pinelema* Wang & Li, 2012**
–	Embolus without spiral ridge, nearly L-shaped	***Sundalema* gen. nov.**

### Key to telemid genera occurring in East and Southeast Asia (Females)

**Table:** 

1	Tibial glands plate-shaped (Fig. 1A; Emerit 1984: fig. A), and endogyne walking-stick shaped, with membranous tubes (Wang et al. 2012: fig. 3C, D)	***Telema* Simon, 1882**
–	Tibial glands belt-shaped (Fig. 1B–G; Emerit 1984: fig. C, D), and endogyne not walking-stick shaped, without tube inside, or with sclerotized or membranous tubes	**2**
2	Leg formula: 1-4-2-3	***Telemofila* Wunderlich, 1995**
–	Leg formula: 1-2-4-3	**3**
3	Receptacle sclerotized	**4**
–	Receptacle membranous	**5**
4	Receptacle long and coiled 1.25 to several loops, not swollen distally (Fig. 24A)	***Sundalema* gen. nov.**
–	Receptacle globular, swollen distally	**6**
5	Receptacle without tubes inside	**7**
–	Receptacle with several membranous tubes inside	***Pinelema* Wang & Li, 2012 and *Apneumonella* Fage, 1921^1^**
6	Neck of receptacle sclerotized, umbrella-shaped (Fig. 23A)	***Siamlema* gen. nov. part I (*S. suea* sp. nov.)**
–	Neck of receptacle membranous, tube-shaped (Fig. 28A)	***Zhuanlema* gen. nov.**
7	Receptacle swollen distally	***Mekonglema* gen. nov. part I^2^**
–	Receptacle not swollen distally	***Mekonglema* gen. nov. part II and *Siamlema* gen. nov. part II^3^**

#### Genus Pinelema

Taxon classificationAnimaliaAraneaeTelemidae

Wang & Li, 2012

88DE0D63-DA08-5AAF-8011-24A921BAE057

Pinelema Wang and Li 2012: 76; Wang and Li 2016: 547; Zhao et al. 2018a: 14; Zhao et al. 2018b: 10.

##### Type species.

*Pinelema bailongensis* Wang & Li, 2012 from Guangxi, China.

##### Diagnosis.

**Figure 1.**
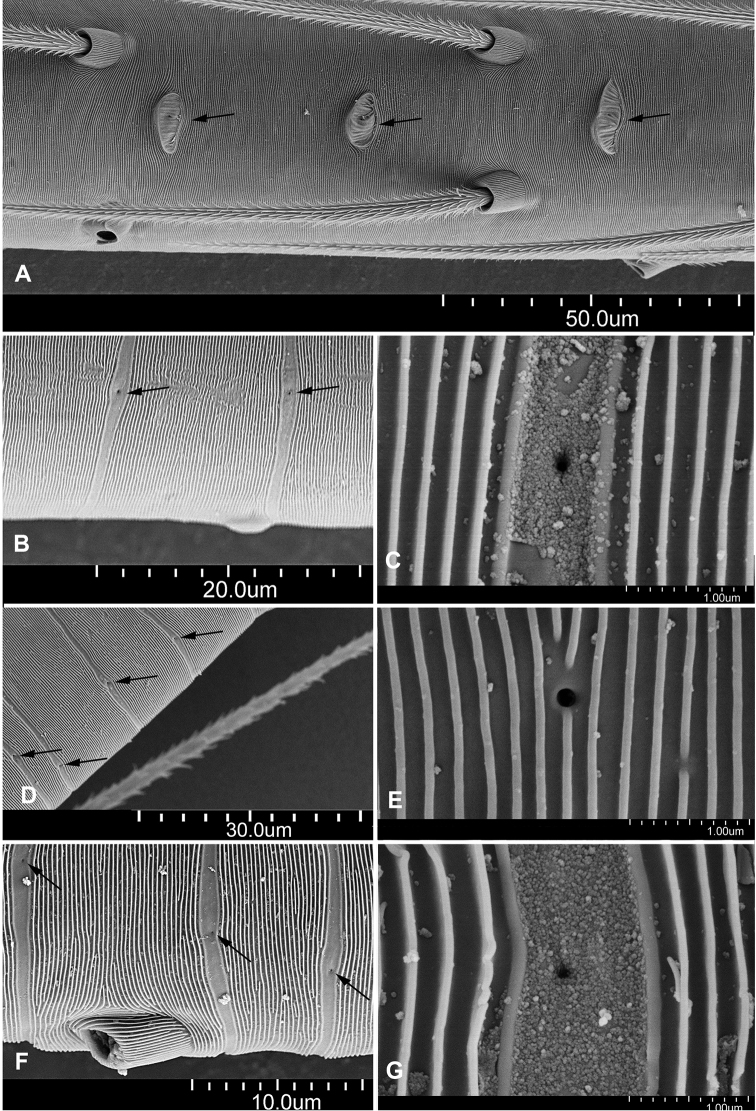
Tibial glands on leg III of telemids in East and Southeast Asia (*Zhuanlema* gen. nov. is not included). **A***Telema guihua***B***Pinelema bailongensis***C***Mekonglema xinpingi* comb. nov. **D***Siamlema changhai* sp. nov. **E***Sundalema anguina* comb. nov. **F***Apneumonella jacobsoni***G***Telemofila samosirensis*.

*Pinelema* can be distinguished from *Telema* by the following: belt-shaped tibial glands (arrows on Fig. 1B) (vs. plate-shaped), the presence of a cymbial apophysis (vs. absent), and the triangular, trapezoidal, or tube-like embolus (vs. duckbill shaped). The endogyne is extended distally (vs. no extension).

##### Description.

Total length: 1.07–1.77 (male), 1.20–2.02 (female). Carapace 0.48–0.82 long. Sternum with several long setae. Six eyes ringed with black, vestigial, or completely absent in some species. Legs thin, long relative to body. Leg formula: 1-2-4-3. Tibia I 0.94–2.20 long, glands of legs belt-shaped (Fig. 1B). Cymbial apophysis distinct, bulb large relative to body, oval, kidney-shaped, droplet-shaped, etc.; embolus triangular, tube-shaped, needle-shaped, etc., embolus short, medium, or long relative to cymbium. Receptacle with several membranous tubes inside and typically swollen distally.

##### Distribution.

China (Guangxi, Guizhou, Yunnan, Hainan), Laos (Vien Tiane), and Vietnam (northern and central Vietnam) (Figs 30–32).

##### Comments.

All 26 new combinations are supported by morphological characters, such as the presence of a cymbial apophysis and the extended tip of the receptacle. Including the new species described in this paper increases the total number of *Pinelema* species to 54, making it the most speciose genus in Telemidae.

##### Composition.

According to the morphological characters of the male palp, 54 *Pinelema* species have been divided into seven species groups as well as six species not attached to a species group. The composition of species groups is discussed below.

#### The adunca

Taxon classificationAnimaliaAraneaeTelemidae

-group

A06C7C6F-C435-521C-826D-140C810B3BB9

Figures 2A , 30

##### Diagnosis.

**Figure 2.**
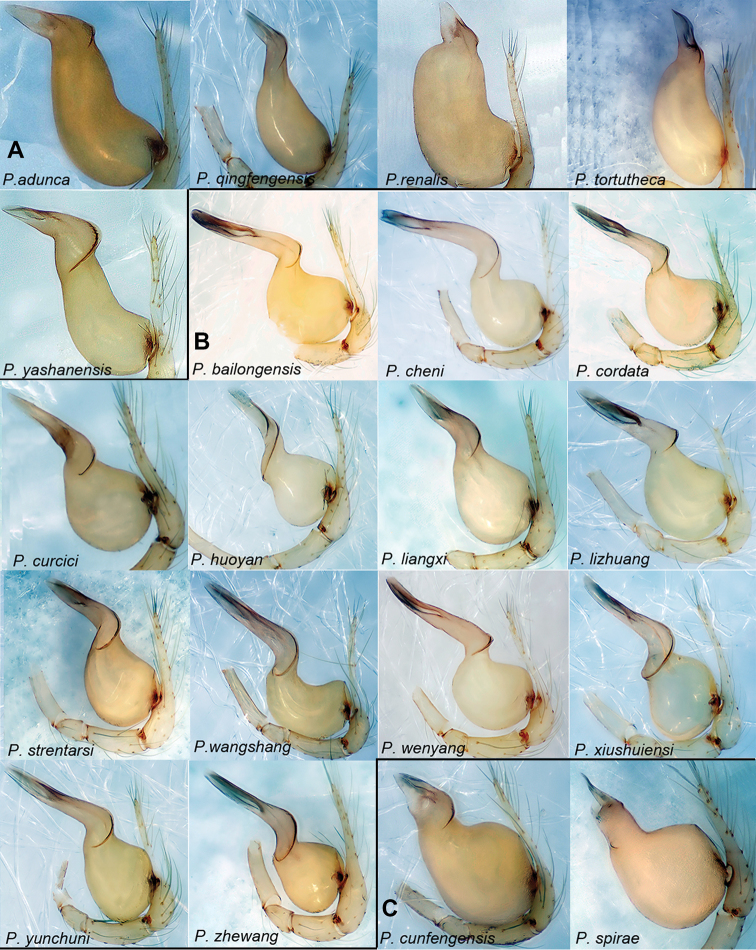
*Pinelema* spp., palp, retrolateral view. **A** The *adunca*-group **B** the *bailongensis*-group **C** the *cunfengensis*-group.

This group resembles the *pacchanensis*-group by having a long bulb but can be distinguished by the bulb is protruding ventro-basally and concave dorso-sub-basally (Fig. 2A) (vs. concave ventro-sub-basally and protruding dorso-mesially).

##### Description.

Body length 1.40–1.98. Carapace 0.60–0.82 long. Six eyes ringed with black or absent. Tibia I 1.40–2.20. Ratio of bulbal length/width 1.96–2.35, bulb protruding ventro-basally and concave dorso-subbasally, embolus membranous (except in *P. tortutheca* (Lin & Li) comb. nov.), length ratio of embolus/bulb 0.38–0.71. Receptacle J-shaped.

##### Distribution.

China (Guangxi, sites 1–5 in Fig. 30).

##### Composition.

*Pinelema adunca* (Wang & Li, 2010) comb. nov., *P. qingfengensis* Zhao & Li, 2017, *P. renalis* (Wang & Li, 2010) comb. nov., *P. tortutheca* comb. nov., and *P. yashanensis* (Wang & Li, 2010) comb. nov.

#### Pinelema adunca

Taxon classificationAnimaliaAraneaeTelemidae

(Wang & Li, 2010) comb. nov.

016DA6D6-5881-56DF-AEE1-9EF806602BE4

Figures 2A , 30

Telema adunca Wang and Li 2010b: 2, figs 1–6 (♂♀).

##### Type material.

Holotype: ♂ (IZCAS), China, Guangxi Zhuang Autonomous Region, Baise Prefecture, Pingguo County, Bafeng Mountain, Guandi Cave, 23.5670N, 107.6794E, elevation ca. 285 m, 3.VIII.2009, C. Wang and Z. Yao leg. Paratypes: 1♂ and 2♀ (IZCAS), same data as holotype. Examined.

##### Other material examined.

1♂ (molecular voucher, IZCAS), same data as holotype.

##### Diagnosis.

*Pinelema adunca* comb. nov. resembles *P. yashanensis* comb. nov. but can be distinguished by the following: the smaller length ratio of the embolus/bulb (0.64, Fig. 2A, and cf. Wang and Li 2010b: fig. 1A, B) (vs. 0.85), the cymbial apophysis which is as long as the cymbial base (cf. Wang and Li 2010b: fig. 1B) (vs. ¼ times shorter); the receptacle tip is 3.20 times wider than the neck (cf. Wang and Li 2010b: fig. 3C) (vs. 1.20 times).

##### Description.

See Wang and Li (2010b).

##### Distribution.

China (Guangxi, site 1 in Fig. 30), known only from the type locality.

#### Pinelema qingfengensis

Taxon classificationAnimaliaAraneaeTelemidae

Zhao & Li, 2017

1E08455D-2C1C-53FC-AE5F-5772173F8393

Figures 2A , 30

Pinelema qingfengensis Song et al. 2017: 91, figs 5, 6, 7D, 8D, 9D, 10D, 11D, 12D (♂♀).

##### Type material.

Holotype: ♂ (IZCAS), China, Guangxi Zhuang Autonomous Region, Chongzuo Prefecture, Tiandeng County, Qingfeng Cave, 23.1720N, 107.1565E, elevation ca. 444 m, 26.XII.2012, Z. Chen and Z. Zhao leg. Paratypes: 1♂ and 2♀ (IZCAS), same data as holotype. Examined.

##### Other material examined.

1♂ (molecular voucher, IZCAS), same data as holotype.

##### Diagnosis.

*Pinelema qingfengensis* resembles *P. renalis* comb. nov. but can be distinguished by the following: the absence of eyes (cf. Song et al. 2017: fig. 5A) (vs. present); the ventrally bent embolus, and the obtuse-angled bulb (Fig. 2A, and cf. Song et al. 2017: fig. 5C, D) (vs. right-angled), the bulb is not protruding ventro-distally (Fig. 2A, and cf. Song et al. 2017: fig. 5C, D) (vs. protruding), and the width ratio of the basal embolus/distal bulb is 1.0 (cf. Song et al. 2017: fig. 5C, D) (vs. 0.5).

##### Description.

See Song et al. (2017).

##### Distribution.

China (Guangxi, site 2 in Fig. 30), known only from the type locality.

#### Pinelema renalis

Taxon classificationAnimaliaAraneaeTelemidae

(Wang & Li, 2010) comb. nov.

14A33749-8CDC-51A1-8A14-B2E620CD8F3B

Figures 2A , 30

Telema renalis Wang and Li 2010b: 24, figs 24–27 (♂♀).

##### Type material.

Holotype: ♂ (IZCAS), China, Guangxi Zhuang Autonomous Region, Hezhou Prefecture, Zhongshan County, Guanyin Cave, 24.5233N, 111.3242E, elevation ca. 196 m, 25.VIII.2009, C. Wang and Z. Yao leg. Paratypes: 1♂ and 2♀ (IZCAS), same data as holotype. Examined.

##### Other material examined.

1♂ (molecular voucher, IZCAS), same data as holotype.

##### Diagnosis.

*Pinelema renalis* comb. nov. resembles *P. qingfengensis* but can be distinguished by the following: six eyes ringed with black (cf. Wang and Li 2010b: fig. 24A) (vs. no eyes); the embolus bent ventrally at a right angle (Fig. 2A, and cf. Wang and Li 2010b: fig. 24C, D) (vs. obtuse angle), the bulb protrudes ventro-distally (Fig. 2A, and cf. Wang and Li 2010b: fig. 24C, D) (vs. not protruding), and the width ratio of the basal embolus/distal bulb is 0.5 (cf. Wang and Li 2010b: fig. 24C, D) (vs. 1.0).

##### Description.

See Wang and Li (2010b).

##### Distribution.

China (Guangxi, site 3 in Fig. 30), known only from the type locality.

#### Pinelema tortutheca

Taxon classificationAnimaliaAraneaeTelemidae

(Lin & Li, 2010) comb. nov.

0B05CC45-1C1F-5F16-8685-AED7A7F60E84

Figures 2A , 30

Telema tortutheca Lin and Li 2010: 26, figs 16, 17 (♂♀).

##### Type material.

Holotype: ♂ (IZCAS), China, Guangxi Zhuang Autonomous Region, Nanning Prefecture, Mashan County, Guling Town, Yangyu Village, Jinlun Cave, 23.5663N, 107.2622E, elevation ca. 1490 m, 6.III.2007, J. Liu and Y. Lin leg. Paratypes: 1♂ and 2♀ (IZCAS), same data as holotype. Examined.

##### Other material examined.

1♂ (molecular voucher, IZCAS), same data as holotype.

##### Diagnosis.

*Pinelema tortutheca* comb. nov. can be distinguished from all four other species in this group by the sclerotized embolus (Fig. 2A, and cf. Lin and Li 2010: fig. 16C) (vs. membranous).

##### Description.

See Lin and Li (2010).

##### Distribution.

China (Guangxi, site 4 in Fig. 30), known only from the type locality.

#### Pinelema yashanensis

Taxon classificationAnimaliaAraneaeTelemidae

(Wang & Li, 2010) comb. nov.

4970D9F3-DCD1-5CDC-BA34-D447A2AAC523

Figures 2A , 30

Telema yashanensis Wang and Li 2010b: 33, figs 28–32 (♂♀).

##### Type material.

Holotype: ♂ (IZCAS), China, Guangxi Zhuang Autonomous Region, Laibin Prefecture, Yashan County, Yashan Cave, 23.6025N, 108.9124E, elevation ca. 115 m, 16.VIII.2009, C. Wang and Z. Yao leg. Paratypes: 1♂ and 2♀ (IZCAS), same data as holotype. Examined.

##### Other material examined.

1♂ (molecular voucher, IZCAS), same data as holotype.

##### Diagnosis.

*Pinelema yashanensis* comb. nov. resembles *P. adunca* comb. nov. but can be distinguished by the following: the larger length ratio of the embolus/bulb (0.85, Fig. 2A, and cf. Wang and Li 2010b: fig. 28A, B) (vs. 0.64); the cymbial apophysis ¼ as long as the cymbial base (cf. Wang and Li 2010b: fig. 28A) (vs. equal length); and the receptacle tip is 1.20 times wider than the neck (cf. Wang and Li 2010b: fig. 30C) (vs. 3.20 times).

##### Description.

See Wang and Li (2010b).

##### Distribution.

China (Guangxi, site 5 in Fig. 30), known only from the type locality.

#### The bailongensis

Taxon classificationAnimaliaAraneaeTelemidae

-group

CF5DE59A-DD2C-566E-A906-B5F5B4CDADCE

Figures 2B , 30

##### Diagnosis.

This group can be distinguished from other species groups in *Pinelema* by the following: the length ratio of the embolus/bulb is 1.19–1.80 (Fig. 2B) (vs. 0.37–0.84), the junction of the bulb and cymbium is located ventro-mesially on the bulb (Fig. 2B) (vs. ventro-basally, except the *cunfengensis*-group).

##### Description.

Body length 1.11–1.75. Carapace 0.50–0.82 long. Tibia I 0.90–2.18 long. Six eyes ringed with black or absent. Embolus longer than bulb, length ratio of embolus/bulb is 1.19–1.80, junction of bulb and cymbium located ventro-mesially on bulb (Fig. 2B), bulb with papillae proximo-retrolaterally (except *P. curcici* Wang & Li, 2016, *P. huoyan* Zhao & Li, 2018, and *P. xiushuiensis* Wang & Li, 2016). Endogyne U-shaped, J-shaped, or spiralled.

##### Distribution.

China (Guangxi, Guizhou, Yunnan, sites 6–18 in Fig. 30).

##### Composition.

*Pinelema bailongensis*, *P. cheni* Zhao & Li, 2018, *P. cordata* (Wang & Li, 2010), *P. curcici*, *P. huoyan*, *P. liangxi* (Zhu & Chen, 2002), *P. lizhuang* Zhao & Li, 2018, *P. strentarsi* (Lin & Li, 2010), *P. wangshang* Zhao & Li, 2018, *P. wenyang* Zhao & Li, 2018, *P. xiushuiensis*, *P. yunchuni* Zhao & Li, 2018, and *P. zhewang* (Chen & Zhu, 2009).

##### Remarks.

For the diagnoses and descriptions of this group (except *P. bailongensis* and *P. curcici*), see Zhao et al. (2018b).

#### Pinelema bailongensis

Taxon classificationAnimaliaAraneaeTelemidae

Wang & Li, 2012

F9ADA276-4235-5A4B-B699-6BD8C7F070C5

Figures 1B , 2B , 30

Pinelema bailongensis Wang and Li 2012: 82, figs 1–17 (♂♀); Song et al. 2017: 85, figs 7A, 8A, 9A, 10A, 11A, 12A (♂); Zhao et al. 2018b: fig. 1 (♂).

##### Type material.

Holotype: ♂ (IZCAS), China, Guangxi Zhuang Autonomous Region, Baise Prefecture, Pingguo County, Bailong Cave, 23.3182N, 107.5731E, elevation ca. 111 m, 1.VIII.2009, C. Wang and Z. Yao leg. Paratypes: 1♂ and 2♀ (IZCAS), same data as holotype. Examined.

##### Other material examined.

1♂ (molecular voucher, IZCAS), same data as holotype.

##### Diagnosis.

For differences between *P. bailongensis* and *P. curcici*, see Wang and Li (2016); for differences between *P. bailongensis* and the other eleven species in this group, see Zhao et al. (2018b).

##### Description-amendments.

Tibial glands belt-shaped (Fig. 1B), the arrangement of secretory orifices linear within a smooth, striped tegument (arrows on Fig. 1B). For a more detailed description, see Wang and Li (2012), Song et al. (2017), and Zhao et al. (2018b).

##### Distribution.

China (Guangxi, site 6 in Fig. 30), known only from the type locality.

#### Pinelema curcici

Taxon classificationAnimaliaAraneaeTelemidae

Wang & Li, 2016

AD249609-EC7E-5784-8D50-BBCE49A63B2B

Figures 2B , 30

Pinelema curcici Wang and Li 2016: 547, figs 1–4 (♂♀).

##### Type material.

Holotype: ♂ (IZCAS), China, Yunnan Province, Wenshan Zhuang and Miao Autonomous Prefecture, Qiubei County, Shuanglongying Town, Fengwei Cave, 24.3361N, 104.2862E, elevation ca. 1372 m, 20.VIII.2010, Z. Yao, C. Wang and X. Wang leg. Paratypes: 1♂ and 2♀ (IZCAS), same data as holotype. Examined.

##### Other material examined.

1♂ (molecular voucher, IZCAS), same data as holotype.

##### Diagnosis.

*Pinelema curcici* resembles *P. liangxi* but can be distinguished by the following: the presence of eyes (vs. absent); the embolic tip is much narrower than the embolic base (Fig. 2B) (vs. equal), the smaller ratio of the bulbal length/width (1.13, Fig. 2B, and cf. Wang and Li 2016: fig. 1C, D) (vs. 1.27), the greater length ratio of the embolus/bulb (1.31, Fig. 2B, and cf. Wang and Li 2016: fig. 1C, D) (vs. 1.19); the J-shaped receptacle (vs. C-shaped), with the distal part of the receptacle 1.75 times wider than the neck of the receptacle (cf. Wang and Li 2016: fig. 2C) (vs. three times).

##### Description.

See Wang and Li (2016).

##### Distribution.

China (Yunnan, site 9 in Fig. 30), known only from the type locality.

#### The cunfengensis

Taxon classificationAnimaliaAraneaeTelemidae

-group

D8D89AB1-9711-5F94-9A35-706965361622

Figures 2C , 30

##### Diagnosis.

This group resembles the *feilong*-group by having a triangular and short embolus relative to the bulb length but can be distinguished by the following: the bulb is bent at a right angle dorso-distally (Fig. 2C) (vs. not bent), and the junction of the bulb and cymbium is located dorso-mesially on the bulb (Fig. 2C) (vs. dorso-basally); the receptacle is U-shaped or coiled (vs. bag-like or globular).

##### Description.

Body length 1.33–1.48. Carapace 0.48–0.64 long. Tibia I 1.15–1.98 long. Six eyes ringed with black (*P. cunfengensis* Zhao & Li, 2017) or absent (*P. spirae* (Lin & Li, 2010) comb. nov.). Bulb bent dorso-distally at a right angle (Fig. 2C), ratio of bulbal length/width 1.22–1.39, length ratio of embolus/bulb 0.38–0.58, embolus triangular. Receptacle U-shaped (*P. cunfengensis*) or coiled (*P. spirae* comb. nov.).

##### Distribution.

China (Guangxi, sites 19–20 in Fig. 30).

##### Composition.

*Pinelema cunfengensis* and *P. spirae* comb. nov.

#### Pinelema cunfengensis

Taxon classificationAnimaliaAraneaeTelemidae

Zhao & Li, 2017

B3AAFD17-F2DA-52DB-B27E-505EF836DF12

Figures 2C , 30

Pinelema cunfengensis Song et al. 2017: 85, figs 1, 2, 7B, 8B, 9B, 10B, 11B, 12B (♂♀).

##### Type material.

Holotype: ♂ (IZCAS), China, Guangxi Zhuang Autonomous Region, Nanning Prefecture, Long’an County, Nanxu Town, Nawan Village, Feng Cave, 23.2098N, 107.5906E, elevation ca. 115 m, 13.V.2015, Z. Chen and Y. Li. leg. Paratypes: 1♂ and 2♀ (IZCAS), same data as holotype. Examined.

##### Other material examined.

1♂ (molecular voucher, IZCAS), same data as holotype.

##### Diagnosis.

*Pinelema cunfengensis* resembles *P. spirae* comb. nov. but can be distinguished by the following: the presence of eyes (cf. Song et al. 2017: fig. 1A) (vs. absence); the different shape of the embolus (Fig. 2C, and cf. Song et al. 2017: fig. 1C, D), the ratio of bulbal length/width is 1.22 (vs. 1.39), and the length ratio of the embolus/bulb is 0.58 (Fig. 2C, and cf. Song et al. 2017: fig. 1C, D) (vs. 0.38); the U-shaped receptacle (cf. Song et al. 2017: fig. 2C) (vs. coiled).

##### Description.

See Song et al. (2017).

##### Distribution.

China (Guangxi, site 19 in Fig. 30), known only from the type locality.

#### Pinelema spirae

Taxon classificationAnimaliaAraneaeTelemidae

(Lin & Li, 2010) comb. nov.

7ADD074C-E991-5534-AD13-8CF7B63FA96D

Figures 2C , 30

Telema spirae Lin and Li 2010: 21, figs 12, 13 (♂♀).

##### Type material.

Holotype: ♂ (IZCAS), China, Guangxi Zhuang Autonomous Region, Hechi Prefecture, Bama County, Poyue Town, Poyue Village, Dawan Cave, 24.3016N, 107.1155E, elevation ca. 438 m, 16.VIII.2007, J. Liu and Y. Lin leg. Paratypes: 1♂ and 2♀ (IZCAS), same data as holotype. Examined.

##### Other material examined.

1♂ (molecular voucher, IZCAS), same data as holotype.

##### Diagnosis.

*Pinelema spirae* comb. nov. resembles *P. cunfengensis* but can be distinguished by the following: the absence of eyes (vs. presence); the different shape of the embolus (Fig. 2C), the ratio of bulbal length/width is 1.39 (vs. 1.22), and the length ratio of the embolus/bulb is 0.38 (Fig. 2C) (vs. 0.58); the coiled receptacle (cf. Lin and Li 2010: fig. 13B, C) (vs. U-shaped).

##### Description.

See Lin and Li (2010).

##### Distribution.

China (Guangxi, site 20 in Fig. 30), known only from the type locality.

#### The feilong

Taxon classificationAnimaliaAraneaeTelemidae

-group

86590941-BFD3-5E4C-A2AE-5F187DDE3592

Figures 3A , 31

##### Diagnosis.

**Figure 3.**
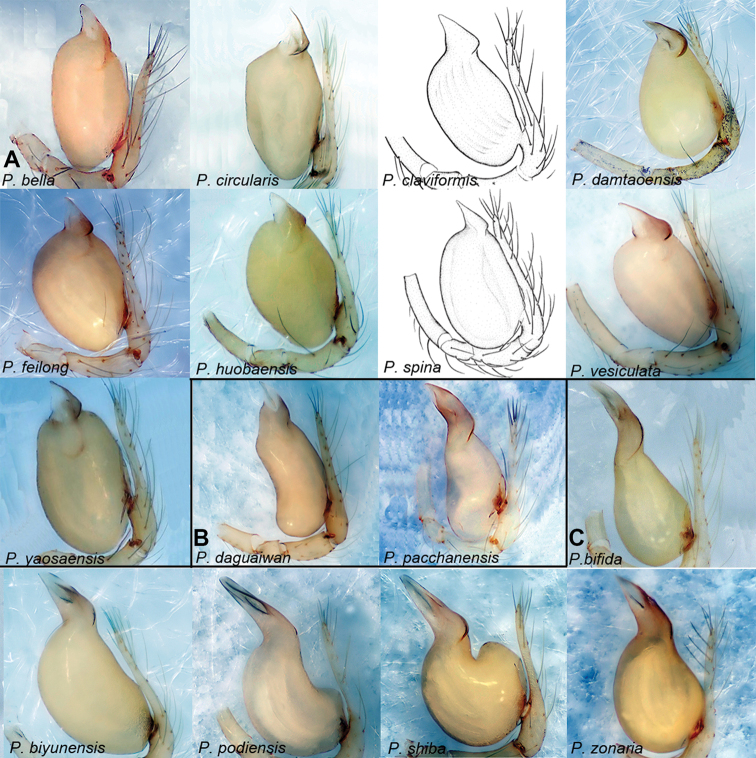
*Pinelema* spp., palp, retrolateral view. **A** The *feilong*-group **B** the *pacchanensis*-group **C** the *podiensis*-group. *P. claviformis* comb. nov. and *P. spina* comb. nov. are modified from Tong and Li 2008b and 2008a, respectively.

This group resembles the *xiezi*-group by the short embolus relative to the bulb but can be distinguished by the triangular shape of the embolus (Fig. 3A) (vs. trapezoidal).

##### Description.

Body length 0.98–1.85. Carapace length 0.47–0.76. Tibia I length 0.85–2.20. Six eyes ringed with black (*P. bella* (Tong & Li, 2008) comb. nov., *P. damtaoensis* Zhao & Li, 2018, and *P. spina* comb. nov.) or absent (other species in this group), bulb oval, the junction of the bulb and the cymbium is located ventro-basally, the embolus is triangular, and the length ratio of the embolus/bulb is 0.33–0.49. The receptacle is stick-shaped (*P. bella* comb. nov., *P. claviformis* comb. nov., and *P. feilong* (Chen & Zhu, 2009) comb. nov.), boot-shaped (*P. circularis* (Tong & Li, 2008) comb. nov.), or globular.

##### Distribution.

China (Guangxi, Yunnan, Hainan) and Vietnam (Vinh Phuc) (Fig. 31).

##### Composition.

*Pinelema bella* comb. nov., *P. circularis* comb. nov., *P. claviformis* comb. nov., *P. damtaoensis*, *P. feilong* comb. nov., *P. huobaensis* Wang & Li, 2016, *P. spina* comb. nov., *P. vesiculata* (Lin & Li, 2010) comb. nov., and *P. yaosaensis* Wang & Li, 2016.

#### Pinelema bella

Taxon classificationAnimaliaAraneaeTelemidae

(Tong & Li, 2008) comb. nov.

39C1A874-45C3-5709-8C2B-372E817AA090

Figures 3A , 31

Telema bella Tong and Li 2008a: 68, figs 1A, 2A–G, 6 (♂♀); Tong 2013: 71, figs 31M, 86A–G (♂♀).

##### Type material.

Holotype: ♂ (IZCAS), China, Hainan Province, Dongfang County, Datian National Natural Reserve, Mihou Cave, 18.9114N, 109.0639E, elevation ca. 293 m, 28.V.2005, Y. Song, X. Han, G. Deng and Y. Tong leg. Paratypes: 1♂ and 2♀ (IZCAS), same data as holotype. Examined.

##### Other material examined.

1♂ (molecular voucher, IZCAS), same data as holotype.

##### Diagnosis.

*Pinelema bella* comb. nov. resembles *P. spina* comb. nov. but can be distinguished by the following: the larger ratio of the bulbal length/width (1.75, Fig. 3A, and cf. Tong and Li 2008a: fig. 2C) (vs. 1.45); the obtuse-angled ventral bend between the embolus and bulb (Fig. 3A, and cf. Tong and Li 2008a: fig. 2B, C) (vs. right-angled), and the blunt tip of the embolus (Fig. 3A, and cf. Tong and Li 2008a: fig. 2B, C) (vs. sharp).

##### Description.

See Tong and Li (2008a).

##### Distribution.

China (Hainan, site 1 in Fig. 31), known only from the type locality.

#### Pinelema circularis

Taxon classificationAnimaliaAraneaeTelemidae

(Tong & Li, 2008) comb. nov.

EF51B997-AC0B-5A52-98FA-6A9C33A0A27D

Figures 3A , 31

Telema circularis Tong and Li 2008b: 363, figs 1A, 2 (♂♀).

##### Type material.

Holotype: ♂ (IZCAS), China, Guizhou Province, Anshun Prefecture, Guanling County, Shangguan Town, Ganzhishu Cave, 25.8120N, 105.6716E, elevation ca. 987 m, 8.V.2005, Y Tong and Y. Lin leg. Paratypes: 1♂ and 2♀ (IZCAS), same data as holotype. Examined.

##### Other material examined.

1♂ (molecular voucher, IZCAS), same data as holotype.

##### Diagnosis.

*Pinelema circularis* comb. nov. resembles *P. claviformis* comb. nov. but can be distinguished by the following: the sharp tip of the embolus (Fig. 3A, and cf. Tong and Li 2008b: fig. 2B, C) (vs. blunt); the boot-shaped receptacle (cf. Tong and Li 2008b: fig. 2D, E) (vs. stick-shaped).

##### Description.

See Tong and Li (2008b).

##### Distribution.

China (Guizhou, site 2 in Fig. 31), known only from the type locality.

#### Pinelema claviformis

Taxon classificationAnimaliaAraneaeTelemidae

(Tong & Li, 2008) comb. nov.

611E324F-E4AC-57BB-A2CE-3C0B4981F347

Figures 3A , 31

Telema claviformis Tong and Li 2008b: 364, figs 1B, 3 (♂♀).

##### Type material.

Holotype: ♂ (IZCAS), China, Guizhou Province, Qianxinan Buyei and Miao Autonomous Prefecture, Xingyi County, Maling Town, Qiuxiang Cave, 25.2000N, 104.8833E, 12.V.2005, Y. Tong and Y. Lin leg. Paratypes: 1♂ and 2♀ (IZCAS). Examined.

##### Diagnosis.

*Pinelema claviformis* comb. nov. resembles *P. circularis* comb. nov. but can be distinguished by the following: the blunt tip of the embolus (Fig. 3A) (vs. sharp); the stick-shaped receptacle (cf. Tong and Li 2008b: fig. 3D, E) (vs. boot-shaped).

##### Description.

See Tong and Li (2008b).

##### Distribution.

China (Guizhou, site 3 in Fig. 31), known only from the type locality.

#### Pinelema damtaoensis

Taxon classificationAnimaliaAraneaeTelemidae

Zhao & Li, 2018

B809D566-A8AF-592C-81BD-D702D7F3B0A0

Figures 3A , 31

Pinelema damtaoensis Zhao et al. 2018a: 15, figs 1–3 (♂♀).

##### Type material.

Holotype: ♂ (IZCAS), Vietnam, Vinh Phuc Province, Dam Tao National Park, leaf litter, 21.4600N, 105.6480E, elevation ca. 999 m, 1.XI.2012, H. Zhao and Z. Chen leg. Paratypes: 1♂ and 2♀ (IZCAS), same data as holotype. Examined.

##### Other material examined.

1♂ (molecular voucher, IZCAS), same data as holotype.

##### Diagnosis.

*Pinelema damtaoensis* resembles *P. bella* comb. nov. but can be distinguished by the following: a black spot and radial stripes on the carapace (cf. Zhao et al. 2018a: fig. 1A) (vs. carapace without pattern); the spiral ridge of the embolus is sclerotized (Fig. 3A, and cf. Zhao et al. 2018a: fig. 1B) (vs. membranous); the receptacle is globular (cf. Zhao et al. 2018a: fig. 3C) (vs. stick-shaped).

##### Description.

See Zhao et al. (2018a).

##### Distribution.

Vietnam (Vinh Phuc, site 4 in Fig. 31), known only from the type locality.

#### Pinelema feilong

Taxon classificationAnimaliaAraneaeTelemidae

(Chen & Zhu, 2009) comb. nov.

B03899B9-30B8-5690-8D4C-FB346A8BBD10

Figures 3A , 31

Telema feilong Chen and Zhu 2009: 1707, fig. 2A–K (♂♀).

##### Type material.

Holotype: ♂ (MLR), China, Guizhou Province, Qianxinan Buyei and Miao Autonomous Prefecture, Xingyi County, Feilong Cave, 24.9166N, 104.8833E, elevation ca. 1335 m, 25.V.2004, H. Chen and Y. Zhang leg. Paratypes: 3♂ and 1♀ (MHBU), same data as holotype. Not examined.

##### Other material examined.

1♂ and 2♀ (including molecular voucher, IZCAS), same data as the type locality, 8.III.2011, C. Wang and L. Lin leg.

##### Diagnosis.

*Pinelema feilong* comb. nov. resembles *P. vesiculata* comb. nov. but can be distinguished by the following: the smaller ratio of bulbal length/width (1.31, Fig. 3A, and cf. Chen and Zhu 2009: fig. 2C,) (vs. 1.44), the smaller length ratio of the embolus/bulb (0.36, Fig. 3A, and cf. Chen and Zhu 2009: fig. 2C, D) (vs. 0.48); the unmodified stick-shaped receptacle (cf. Chen and Zhu 2009: fig. 2J) (vs. receptacle with a vesicle ventro-distally).

##### Description.

See Chen and Zhu (2009).

##### Distribution.

China (Guizhou, site 5 in Fig. 31), known only from the type locality.

#### Pinelema huobaensis

Taxon classificationAnimaliaAraneaeTelemidae

Wang & Li, 2016

2E194189-C8A4-5DCE-B2C7-4CF0E1D95E89

Figures 3A , 31

Pinelema huobaensis Wang and Li 2016: 556, figs 5–8 (♂♀).

##### Type material.

Holotype: ♂ (IZCAS), China, Yunnan Province, Wenshan Zhuang and Miao Autonomous Prefecture, Qiubei County, Shuanglongying Town, Puzhehei Village, Huoba Cave, 24.1385N, 104.1126E, elevation ca. 1457 m, 18.VIII.2009, Z. Yao, C. Wang and X. Wang leg. Paratypes: 1♂ and 1♀ (IZCAS), same data as holotype. Examined.

##### Other material examined.

1♂ (molecular voucher, IZCAS), same data as holotype.

##### Diagnosis.

*Pinelema huobaensis* resembles *P. yaosaensis* but can be distinguished by the following: the bulb protrudes ventro-subdistally (Fig. 3A, and cf. Wang and Li 2016: fig. 5B, C) (vs. protrudes dorso-distally); the distal part of the receptacle is five times wider than the neck of the receptacle (cf. Wang and Li 2016: fig. 6C) (vs. eight times).

##### Description.

See Wang and Li (2016).

##### Distribution.

China (Yunnan, site 6 in Fig. 31), known only from the type locality.

#### Pinelema spina

Taxon classificationAnimaliaAraneaeTelemidae

(Tong & Li, 2008) comb. nov.

04381E80-6714-50AB-B789-1E202FA63ED7

Figures 3A , 31

Telema spina Tong and Li 2008a: 73, figs 1D, 5, 6 (♂); Tong 2013: 73, figs 31P, 89A–C (♂).

##### Type material.

Holotype: ♂ (IZCAS), China, Hainan Province, Wuzhishan County, Wuzhi Mountain, leaf litter, 18.8167N, 109.6500E, Y. Song leg. Examined.

##### Diagnosis.

*Pinelema spina* comb. nov. resembles *P. bella* comb. nov. but can be distinguished by the following: the smaller ratio of the bulbal length/width (1.45, Fig. 3A) (vs. 1.75); the obtuse-angled ventral bend between the embolus and bulb (Fig. 3A) (vs. right-angled), the sharp tip of the embolus (Fig. 3A) (vs. blunt).

##### Description.

See Tong and Li (2008a).

##### Distribution.

China (Hainan, site 7 in Fig. 31), known only from the type locality.

#### Pinelema vesiculata

Taxon classificationAnimaliaAraneaeTelemidae

(Lin & Li, 2010) comb. nov.

B5407746-8D2F-592C-BF63-5E4F4503E282

Figures 3A , 31

Telema vesiculata Lin and Li 2010: 29, figs 18–20 (♂♀).

##### Type material.

Holotype: ♂ (IZCAS), China, Yunnan Province, Qujing Prefecture, Luoping County, Luoxiong Town, Pingtian Village, Laobie Cave, 24.8419N, 104.2678E, elevation ca. 1490 m, 20.III.2007, J. Liu and Y. Lin leg. Paratypes: 1♂ and 2♀ (IZCAS), same data as holotype. Examined.

##### Other material examined.

1♂ (molecular voucher, IZCAS), same data as holotype.

##### Diagnosis.

*Pinelema vesiculata* comb. nov resembles *P. feilong* comb. nov. but can be distinguished by the following: the larger ratio of the bulbal length/width (1.44, Fig. 3A, and cf. Lin and Li 2010: fig. 18D) (vs. 1.31), the larger length ratio of the embolus/bulb (Fig. 3A, and 0.48, cf. Lin and Li 2010: fig. 18E) (vs. 0.36); the receptacle with a vesicle ventro-distally (cf. Lin and Li 2010: fig. 20B–D) (vs. unmodified).

##### Description.

See Lin and Li (2010).

##### Distribution.

China (Yunnan, site 8 in Fig. 31), known only from the type locality.

#### Pinelema yaosaensis

Taxon classificationAnimaliaAraneaeTelemidae

Wang & Li, 2016

6C8CC31E-E34A-5CCD-B939-D53CE7A95449

Figures 3A , 31

Pinelema yaosaensis Wang and Li 2016: 561, figs 13–17 (♂♀).

##### Type material.

Holotype: ♂ (IZCAS), China, Yunnan Province, Wenshan Zhuang and Miao Autonomous Prefecture, Xichou County, Niuchangba Township, Mosa Village, Yaosa Cave, 23.5072N, 104.9023E, elevation ca. 1297 m, 6.VIII.2009, Z. Yao, C. Wu and X. Wang leg. Paratypes: 2♀ (IZCAS), same data as holotype. Examined.

##### Other material examined.

1♂ (molecular voucher, IZCAS), same data as holotype.

##### Diagnosis.

*Pinelema yaosaensis* resembles *P. huobaensis* but can be distinguished by the following: the bulb does not protrude ventro-subdistally but protrudes dorso-distally (Fig. 3A, and cf. Wang and Li 2016: fig. 13B, C) (vs. protruding ventro-subdistally but not dorso-distally); the distal part of the receptacle is eight times wider than the neck of the receptacle (cf. Wang and Li 2016: fig. 14C) (vs. five times).

##### Description.

See Wang and Li (2016).

##### Distribution.

China (Yunnan, site 9 in Fig. 31), known only from the type locality.

#### The pacchanensis

Taxon classificationAnimaliaAraneaeTelemidae

-group

37C7F4DC-F44B-55A4-8469-8EC8860EA6EA

Figures 3B , 31

##### Diagnosis.

This group resembles the *adunca*-group by having a long bulb but can be distinguished by the bulb being slightly concave ventrally and protruding dorso-mesially (Fig. 3B) (vs. protrude ventro-basally and concave dorso-subbasally).

##### Description.

Body length 1.33–1.60. Carapace 0.56–0.71 long. Eyes absent. Tibia I 1.33–2.00 long. Ratio of bulbal length/width 1.80–2.11, bulb slightly concave ventrally and protruding dorso-mesially (Fig. 3B), embolus triangular or tube-like. Receptacle swollen distally and globular.

##### Distribution.

China (Guizhou) and Vietnam (Bac Kan) (sites 10–11 in Fig. 31)

##### Composition.

*Pinelema daguaiwan* sp. nov. and *P. pacchanensis* Zhao & Li, 2018.

#### Pinelema daguaiwan

Taxon classificationAnimaliaAraneaeTelemidae

Zhao & Li sp. nov.

A714C17B-9D77-5F9C-A728-19FBF5058588

http://zoobank.org/9B313412-1CF2-487E-B1C3-F18992851E01

**Figure 4.**
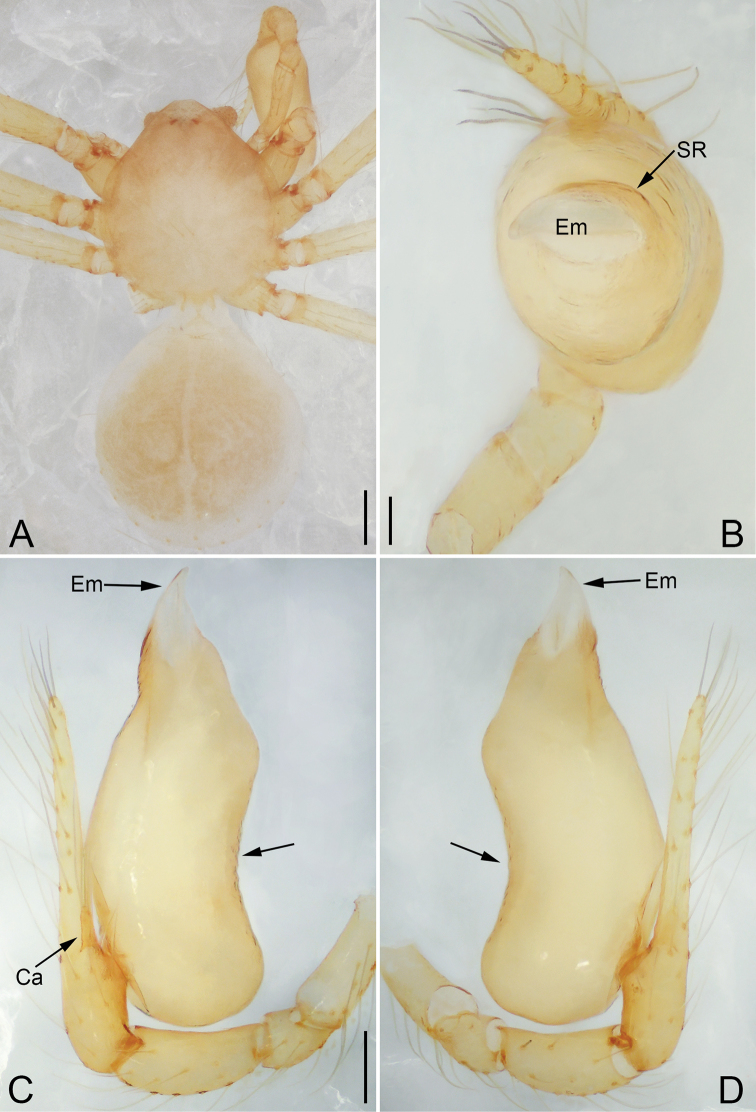
*Pinelema daguaiwan* sp. nov., male holotype. **A** Habitus, dorsal view **B** embolus, apical view **C** palp, prolateral view **D** palp, retrolateral view. Scale bars: 0.2 mm (**A**), 0.05 mm (**B**), 0.1 mm (**C, D**).

**Figure 5.**
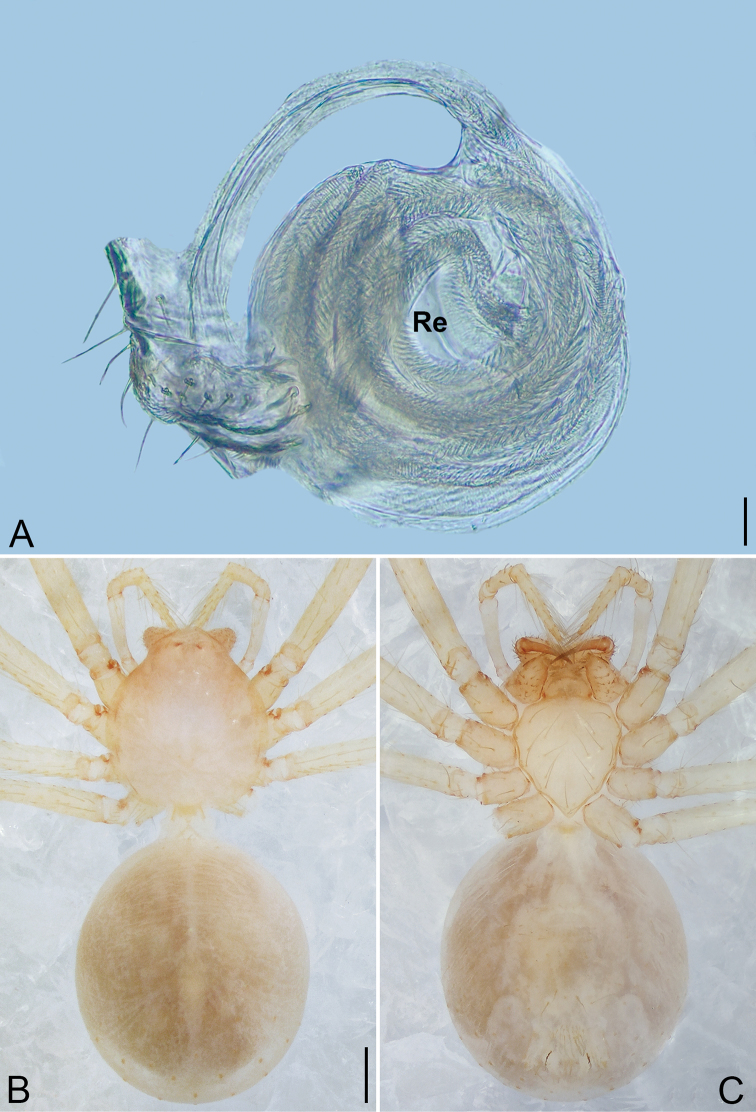
*Pinelema daguaiwan*, sp. nov., female paratype. **A** Endogyne, lateral view **B** habitus, dorsal view **C** habitus, ventral view. Scale bars: 0.05 mm (**A**), 0.2 mm (**B, C**).

Figures 3B , 4 , 5 , 31

##### Type material.

Holotype: ♂ (IZCAS), China, Guizhou Province, Qianxinan Buyi and Miao Autonomous Prefecture, Wangmo County, Sanglang Town, Xinghe Village, Daguaiwan Cave. 25.2706N, 106.4328E, elevation ca. 886 m, 29.XII.2010, Z. Zha and Z. Chen leg. Paratypes: 1♂ and 5♀ (IZCAS), same data as holotype.

##### Etymology.

The species name refers to the type locality; noun in apposition.

##### Diagnosis.

*Pinelema daguaiwan* sp. nov. resembles *P. pacchanensis* by the bulb being concave ventrally but can be distinguished by the following: the bulb is more deeply concave (arrow in Fig. 4C, D) (vs. weaker), the smaller length ratio of the embolus/bulb (0.48, Figs 3B, 4C, D) (vs. 0.71), the triangular embolus (Figs 3B, 4C, D) (vs. tube-shaped); the diameter ratio of the receptacle tip/neck is 7.0 (Fig. 5A) (vs. 2.0).

##### Description.

**Male (holotype).** Total length 1.60. Carapace 0.71 long, 0.67 wide. Abdomen 0.85 long, 0.76 wide. Carapace light brown (Fig. 4A). Eyes absent (Fig. 4A). Chelicerae, legs, labium, and endites brown. Sternum light brown with sparse setae. Leg measurements: I 6.25 (1.80, 0.26, 2.00, 1.41, 0.78); II 5.46 (1.56, 0.26, 1.74, 1.19, 0.71); III (1.20, 0.25, 1.14, –, –); IV 4.96 (1.52, 0.24, 1.44, 1.13, 0.63). Abdomen pale brown (Fig. 4A).

Palp. Tibia two times longer than patella, cymbium 2.47 times longer than tibia, 1.88 times longer than femur, cymbial apophysis finger shaped (Fig. 4C); bulb concave ventrally, and shaped as in Fig. 4C, D, length ratio of bulb/cymbium about 0.9; embolus triangular and membranous, 1/5 as long as bulb (Fig. 4C, D), spiral ridge brown (Fig. 4B).

**Female.** Total length 1.72. Carapace 0.66 long, 0.59 wide. Abdomen 1.04 long, 0.87 wide. Coloration as in male (Fig. 5B, C). Leg measurements: I 5.33 (1.56, 0.24, 1.68, 1.15, 0.70); II 4.59 (1.33, 0.24, 1.41, 0.99, 0.62); III 3.37 (1.05, 0.22, 0.94, 0.67, 0.49); IV 4.15 (1.25, 0.22, 1.21, 0.91, 0.56). Abdomen pale brown (Fig. 5B, C). Neck of receptacle membranous, as long as diameter of receptacle (Fig. 5A); receptacle tip globular with several membranous tubes, much wider than neck (Fig. 5A).

##### Distribution.

China (Guizhou, site 10 in Fig. 31), known only from the type locality.

#### Pinelema pacchanensis

Taxon classificationAnimaliaAraneaeTelemidae

Zhao & Li, 2018

5822D1F7-7042-585A-ADF0-70939573B055

Figures 3B , 31

Pinelema pacchanensis Zhao et al. 2018a: 26, figs 10–12 (♂♀).

##### Type material.

Holotype: ♂ (IZCAS), Vietnam, Bac Kan Province, Cho Don District, Pac Chan Cave, 22.3790N, 105.6130E, elevation ca. 225 m, 18.X.2012, H. Zhao and Z. Chen leg. Paratypes: 1♂ and 2♀ (IZCAS), same data as holotype. Examined.

##### Other material examined.

1♂ (molecular voucher, IZCAS), same data as holotype.

##### Diagnosis.

*Pinelema pacchanensis* resembles *P. daguaiwan* sp. nov. but can be distinguished by the following: the ventrally concave part of the bulb is weaker (Fig. 3B, and cf. Zhao et al. 2018a: fig. 11A, B) (vs. stronger), the larger length ratio of the embolus/bulb (0.71, Fig. 3B, and cf. Zhao et al. 2018a: fig. 11A, B) (vs. 0.48), and the triangular embolus (cf. Zhao et al. 2018a: fig. 11 A–D) (vs. tube-shaped); the diameter ratio of the receptacle tip/neck is 2.0 (cf. Zhao et al. 2018a: fig. 12C) (vs. 7.0).

##### Description.

See Zhao et al. (2018a).

##### Distribution.

Vietnam (Bac Kan, site 11 in Fig. 31), known only from the type locality.

#### The podiensis

Taxon classificationAnimaliaAraneaeTelemidae

-group

C57B2566-0C3B-5CDF-96FB-C8EF3AB041B6

Figures 3C , 31

##### Diagnosis.

This group resembles the *feilong*-group by the embolus which is short relative to the bulb but can be distinguished by the following: the length ratio of the embolus/bulb ranges from 0.57 to 0.79 (Fig. 3C) (vs. 0.33 to 0.49), and the shape of the embolus is a long isosceles triangle (Fig. 3C) (vs. equilateral triangle).

##### Description.

Body length 1.22–1.75. Carapace 0.48–0.75 long. Tibia I 1.15–1.87 long. Six eyes ringed with black (*P. biyunensis* (Wang & Li, 2010) comb. nov., *P. shiba* sp. nov., and *P. zonaria* (Wang & Li, 2010) comb. nov.), vestigial (*P. podiensis* Zhao & Li, 2017), or absent (*P. bifida* (Lin & Li, 2010) comb. nov.). Ratio of bulbal length/width 1.31–1.67, embolus shorter than bulb, length ratio of embolus/bulb 0.57–0.79, embolic shape long isosceles triangle (Fig. 3C). Receptacle J-shaped, slightly swollen distally.

##### Distribution.

China (Guangxi, sites 10–14 in Fig. 31).

##### Composition.

*Pinelema bifida* comb. nov., *P. biyunensis* comb. nov., *P. podiensis*, *P. shiba* sp. nov., and *P. zonaria* comb. nov.

#### Pinelema bifida

Taxon classificationAnimaliaAraneaeTelemidae

(Lin & Li, 2010) comb. nov.

F0B46613-9703-559F-9D83-B751DE009A65

Figures 3C , 31

Telema bifida Lin and Li 2010: 5, figs 2, 3 (♂♀).

##### Type material.

Holotype: ♂ (IZCAS), China, Guangxi Zhuang Autonomous Region, Dahua County, Qibainong Town, Qiaoxu Village, Qiaoxu Cave, 24.0761N, 107.6706E, elevation ca. 550 m, 9.III.2007, J. Liu and Y. Lin leg. Paratypes: 1♂ and 2♀ (IZCAS), same data as holotype. Examined.

##### Other material examined.

1♂ (molecular voucher, IZCAS), same data as holotype.

##### Diagnosis

. *Pinelema bifida* comb. nov. resembles *P. zonaria* comb. nov. but can be distinguished by the following: the absence of eyes (vs. present); the bulb does not protrude ventro-distally (Fig. 3C, and cf. Lin and Li 2010: fig. 2C, D) (vs. protruding), the ratio of bulbal length/width is larger (1.43, cf. Lin and Li 2010: fig. 2C, D) (vs. 1.31).

##### Description.

See Lin and Li (2010).

##### Distribution.

China (Guangxi, site 12 in Fig. 31), known only from the type locality.

#### Pinelema biyunensis

Taxon classificationAnimaliaAraneaeTelemidae

(Wang & Li, 2010) comb. nov.

F3091877-3B85-522F-81C5-9D43AB5E8867

Figures 3C , 31

Telema biyunensis Wang and Li 2010b: 9, figs 7–10 (♂♀).

##### Type material.

Holotype: ♂ (IZCAS), China, Guangxi Zhuang Autonomous Region, Hezhou Prefecture, Zhongshan County, Biyun Cave, 24.357N, 111.1923E, elevation ca. 131 m, 25.VIII.2009, C. Wang and Z. Yao leg. Paratypes: 1♂ and 2♀ (IZCAS), same data as holotype. Examined.

##### Other material examined.

1♂ (molecular voucher, IZCAS), same data as holotype.

##### Diagnosis.

*Pinelema biyunensis* comb. nov. resembles *P. zonaria* comb. nov. but can be distinguished by the following: the larger ratio of bulbal length/width (1.59, Fig. 3C, and cf. Wang and Li 2010b: fig. 7C, D) (vs. 1.31), the smaller length ratio of the embolus/bulb (0.57, Fig. 3C, and cf. Wang and Li 2010b: fig. 7C, D) (vs. 0.76); the tip of the receptacle is 2.10 times wider than the neck (cf. Wang and Li 2010b: fig. 8C) (vs. 1.10 times).

##### Description.

See Wang and Li (2010b).

##### Distribution.

China (Guangxi, site 13 in Fig. 31), known only from the type locality.

#### Pinelema podiensis

Taxon classificationAnimaliaAraneaeTelemidae

Zhao & Li, 2017

D87E9807-1B04-5683-81EF-BAD7E309F5E6

Figures 3C , 31

Pinelema podiensis Song et al. 2017: 88, figs 3, 4, 7C, 8C, 9C, 10C, 11C, 12C (♂♀).

##### Type material.

Holotype: ♂ (IZCAS), China, Guangxi Zhuang Autonomous Region, Baise Prefecture, Debao County, Podi Cave, 23.3919N, 106.6400E, elevation ca. 578 m, 4.VIII.2011, C. Wang leg. Paratypes: 1♂ and 2♀ (IZCAS), same data as holotype. Examined.

##### Other material examined.

1♂ (molecular voucher, IZCAS), same data as holotype.

##### Diagnosis.

*Pinelema podiensis* resembles *P. shiba* sp. nov. but can be distinguished by the following: the weakly concave dorsum of the bulb (Fig. 3C, and cf. Song et al. 2017: fig. 3C, D) (vs. strongly concave), the larger bulbal length/width ratio (1.75, Fig. 3C, and cf. Song et al. 2017: fig. 3C, D) (vs. 1.33); the tip of the receptacle is 2.20 times wider than the neck (cf. Song et al. 2017: fig. 4C) (vs. 4.10 times).

##### Description.

See Song et al. (2017).

##### Distribution.

China (Guangxi, site 14 in Fig. 31), known only from the type locality.

#### Pinelema shiba

Taxon classificationAnimaliaAraneaeTelemidae

Zhao & Li sp. nov.

EE4B27F4-F28F-51CB-A61D-42AE2FC1FCF5

http://zoobank.org/8DB8ED40-DC1F-46D3-B2ED-E2B7E6C8BEE0

**Figure 6.**
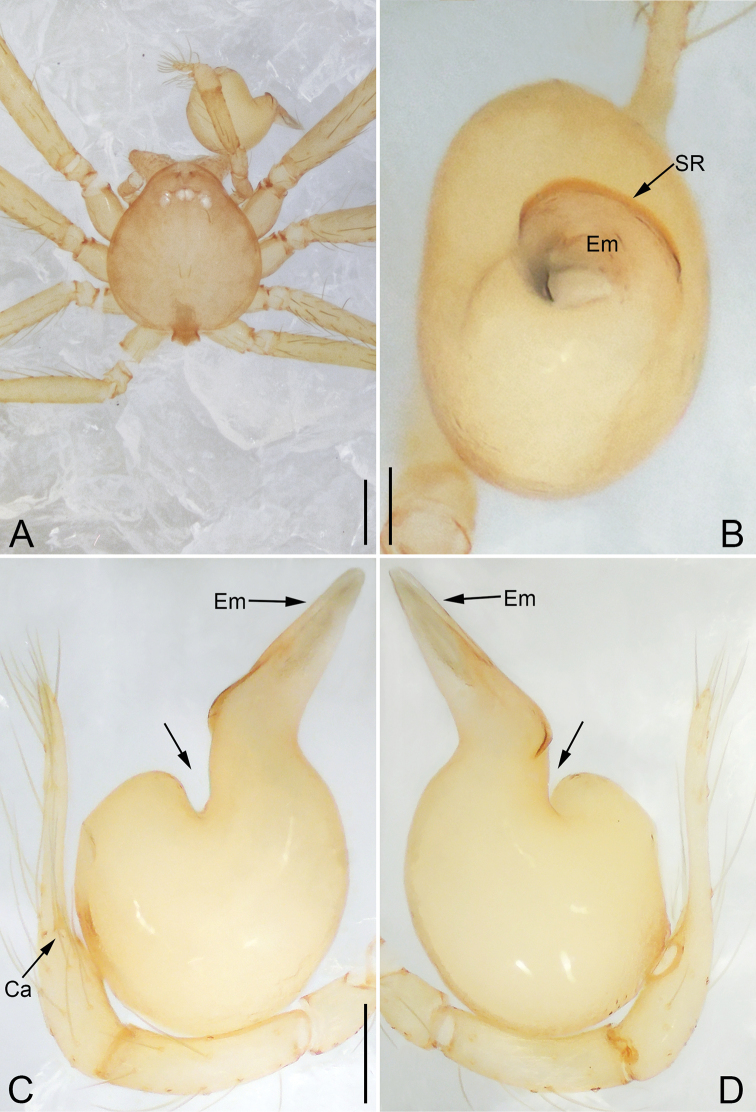
*Pinelema shiba* sp. nov., male holotype. **A** Habitus, dorsal view **B** embolus, apical view **C** palp, prolateral view **D** palp, retrolateral view. Scale bars: 0.2 mm (**A**), 0.05 mm (**B**), 0.1 mm (**C, D**).

**Figure 7.**
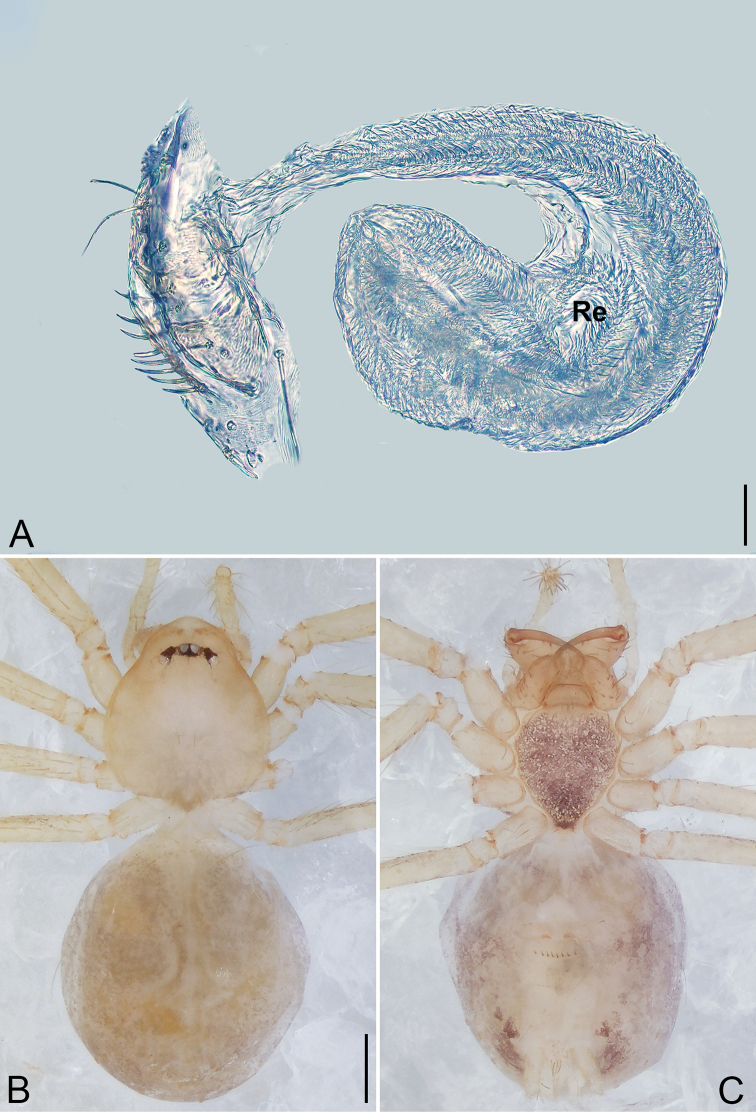
*Pinelema shiba* sp. nov., female paratype. **A** Endogyne, lateral view **B** habitus, dorsal view **C** habitus, ventral view. Scale bars: 0.05 mm (**A**), 0.2 mm (**B, C**).

Figures 3C , 6 , 7 , 31

##### Type material.

Holotype: ♂ (IZCAS); China, Guangxi Zhuang Autonomous Region, Chongzuo Prefecture, Daxin County, Hucheng Town, Baoxian Village, Shiba Cave. 22.8133N, 107.1632E, elevation ca. 157 m. 17.XI.2011, C. Wang leg. Paratypes: 1♂ and 2♀ (IZCAS), same data as holotype.

##### Etymology.

The species name refers to the type locality; noun in apposition.

##### Diagnosis.

*Pinelema shiba* sp. nov. resembles *P. podiensis* but can be distinguished by the following: the strongly concave dorso-mesial part of the bulb (Fig. 6C, D) (vs. weakly concave), the ratio of the bulbal length/width is 1.33 (Fig. 6C, D) (vs. 1.75); the tip of the receptacle is 4.10 times wider than the neck (Fig. 7C) (vs. 2.20 times).

##### Description.

**Male (holotype).** Total length 1.22. Carapace 0.51 long, 0.48 wide. Abdomen 0.71 long, 0.63 wide. Carapace brown (Fig. 6A). Six vestigial eyes (Fig. 6A). Chelicerae, legs, labium, and endites light brown (Fig. 6A). Sternum light brown with sparse setae. Leg measurements: I 5.00 (1.47, 0.21, 1.60, 1.06, 0.66); II 4.26 (1.28, 0.21, 1.33, 0.88, 0.56); III 3.05 (0.97, 0.18, 0.89, 0.56, 0.45); IV 3.55 (1.23, 0.17, 1.00, 0.65, 0.50). Abdomen pale yellow with a few long setae.

Palp. Tibia 2.30 times longer than patella, cymbium 1.91 times longer than tibia, 1.90 times longer than femur, cymbial apophysis short, as long as 1/4 width of cymbium base (Fig. 6C); bulb strongly concave dorsally (arrow in Fig. 6C, D); embolus triangular and half as long as cymbium (Fig. 6C, D), spiral ridge brown (Fig. 6B).

**Female.** Total length 1.39. Carapace 0.54 long, 0.50 wide. Abdomen 0.82 long, 0.73 wide. Six eyes ringed with black (Fig. 7B). Coloration similar to male (Fig. 7B, C). Leg measurements: I 4.94 (1.52, 0.21, 1.56, 1.00, 0.65); II 4.18 (1.31, 0.20, 1.27, 0.85, 0.55); III 3.08 (1.00, 0.19, 0.91, 0.53, 0.45); IV 3.92 (1.25, 0.19, 1.19, 0.78, 0.51). Abdomen brown (Fig. 7B, C). Receptacle U-shaped with several membranous tubes, distally swollen (Fig. 7A).

##### Distribution.

China (Guangxi, site 15 in Fig. 31), known only from the type locality.

#### Pinelema zonaria

Taxon classificationAnimaliaAraneaeTelemidae

(Wang & Li, 2010) comb. nov.

D27EC30A-82C9-5108-B747-2F01450ED436

Figures 3C , 31

Telema zonaria Wang and Li 2010b: 33, figs 33–38 (♂♀).

##### Type material.

Holotype: ♂ (IZCAS), China, Guangxi Zhuang Autonomous Region, Hechi Prefecture, Yizhou County, Xiannv Cave, 24.4887N, 108.5701E, elevation ca. 205 m, 28.VII.2009, C. Wang and Z. Yao leg. Paratypes: 1♂ and 2♀ (IZCAS), same data as holotype. Examined.

##### Other material examined.

1♂ (molecular voucher, IZCAS), same data as holotype.

##### Diagnosis.

*Pinelema zonaria* comb. nov. resembles *P. bifida* comb. nov. but can be distinguished by the following: eyes are present (vs. absent); the bulb protrudes ventro-distally (Fig. 3C, and cf. Wang and Li 2010b: fig. 33C, D) (vs. not protrude), the ratio of the bulbal length/width is smaller (1.31, Fig. 3C, and cf. Wang and Li 2010b: fig. 33C, D) (vs. 1.43).

##### Description.

See Wang and Li (2010b).

##### Distribution.

China (Guangxi, site 16 in Fig. 31), known only from the type locality.

#### The xiezi

Taxon classificationAnimaliaAraneaeTelemidae

-group

D1A12473-A402-5C02-9EF4-6B3554C30978

Figures 8 , 32

##### Diagnosis.

**Figure 8.**
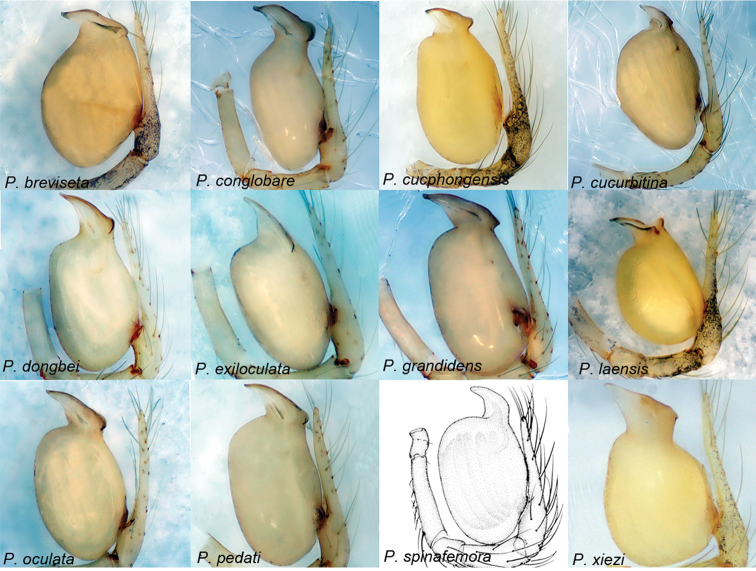
*Pinelema* spp., palp, retrolateral view. The *xiezi*-group. *P. spinafemora* comb. nov. is modified from Lin and Li 2010.

This group resembles the *feilong*-group by the short embolus relative to the bulb but can be distinguished by the trapezoidal shape of the embolus (Fig. 8) (vs. triangular).

##### Description.

Body length 0.98–2.05. Carapace 0.51–0.82 long. Tibia I 0.81–2.13 long. Six eyes ringed with black, vestigial (*P. exiloculata* (Lin, Pham & Li, 2009) comb. nov.), or absent, bulb oval, junction of bulb and cymbium located ventro-basally on bulb, embolus trapezoidal, length ratio of embolus/bulb 0.30–0.59. Receptacle J-shaped.

##### Distribution.

China (Guangxi, Guizhou, Hainan) and Vietnam (Hai Phong, Ninh Binh, Phu Tho, Quang Binh) (Sites 1–12 in Fig. 32).

##### Composition.

*Pinelema breviseta* (Tong & Li, 2008) comb. nov., *P. conglobare* (Lin & Li, 2010) comb. nov., *P. cucphongensis* (Lin, Pham & Li, 2009) comb. nov., *P. cucurbitina* (Wang & Li, 2010) comb. nov., *P. dongbei* (Wang & Ran, 1998) comb. nov., *P. exiloculata* comb. nov., *P. grandidens* (Tong & Li, 2008) comb. nov., *P. laensis* Zhao & Li, 2018, *P. oculata* (Tong & Li, 2008) comb. nov., *P. pedati* (Lin & Li, 2010) comb. nov., *P. spinafemora* (Lin & Li, 2010) comb. nov., and *P. xiezi* Zhao & Li, 2018.

#### Pinelema breviseta

Taxon classificationAnimaliaAraneaeTelemidae

(Tong & Li, 2008) comb. nov.

5CBD5F6F-7513-5EA5-A3C9-182830C2DA9A

Figures 8 , 32

Telema breviseta Tong and Li 2008a: 69, figs 1B, 3A–I, 6 (♂♀); Tong 2013: 72, figs 31O, 87A–I (♂♀).

##### Type material.

Holotype: ♂ (IZCAS), China, Hainan Province, Dongfang County, Donghe Town, Yalong Village, Yalong Cave, 18.9770N, 108.8935E, elevation ca. 273 m, Y. Song, X. Han, G. Deng and Y. Tong leg. 1.IV.2005. Paratypes: 1♂ and 2♀ (IZCAS), same data as holotype. Examined.

##### Other material examined.

1♂ (molecular voucher, IZCAS), same data as holotype.

##### Diagnosis.

*Pinelema breviseta* comb. nov. resembles *P. laensis* but can be distinguished by the following: the ventro-distal bulb does not protrude (Fig. 8, and cf. Tong and Li 2008a: fig. 3B, C) (vs. protrude), the embolus lacks modification retrolaterally (Fig. 8, and cf. Tong and Li 2008a: fig. 3B, C) (vs. embolus with a vertical groove retrolaterally); the tip of the receptacle is three times wider than the neck (cf. Tong and Li 2008a: fig. 3H) (vs. four times).

##### Description.

See Tong and Li (2008a).

##### Distribution.

China (Hainan, site 1 in Fig. 32), known only from the type locality.

#### Pinelema conglobare

Taxon classificationAnimaliaAraneaeTelemidae

(Lin & Li, 2010) comb. nov.

27F066E2-340E-53F3-A93D-17EEFCA72DF2

Figures 8 , 32

Telema conglobare Lin and Li 2010: 8, figs 4, 5 (♂♀).

##### Type material.

Holotype: ♂ (IZCAS), China, Guangxi Zhuang Autonomous Region, Hechi Prefecture, Fengshan County, Fengcheng Town, Songren Village, Xi’an Cave, 24.5657N, 107.0411E, elevation ca. 574 m, 12.III.2007, J. Liu and Y. Lin leg. Paratypes: 1♂ and 2♀ (IZCAS), same data as holotype. Examined.

##### Other material examined.

1♂ (molecular voucher, IZCAS), same data as holotype.

##### Diagnosis.

*Pinelema conglobare* comb. nov. resembles *P. pedati* comb. nov. but can be distinguished by the following: the larger ratio of bulbal length/width (1.77, Fig. 8, and cf. Lin and Li 2010: fig. 4D, E) (vs. 1.60), the ventral bend of the embolus and bulb is right-angled (Fig. 8, and cf. Lin and Li 2010: fig. 4D, E) (vs. acute-angled); the distal part of the receptacle is globular (cf. Lin and Li 2010: fig. 5B–E) (vs. boot-shaped).

##### Description.

See Lin and Li (2010).

##### Distribution.

China (Guangxi, site 2 in Fig. 32), known only from the type locality.

#### Pinelema cucphongensis

Taxon classificationAnimaliaAraneaeTelemidae

(Lin, Pham & Li, 2009) comb. nov.

080F5BDB-1606-5A45-89E6-AF2DEE86A495

Figures 8 , 32

Telema cucphongensis Lin et al. 2009: 327, figs 5A–E, 6A–I (♂♀).

##### Type material.

Holotype: ♂ (IZCAS), Vietnam, Cuc Phuong National Park, Prehistoric Man Cave, 20.2930N, 105.6670E, elevation ca. 256 m, 19.VII.2008, S. Li leg. Paratypes: 1♂ and 2♀ (IZCAS), same data as holotype. Examined.

##### Other material examined.

1♂ (molecular voucher, IZCAS), same data as holotype.

##### Diagnosis.

*Pinelema cucphongensis* comb. nov. resembles *P. xiezi* but can be distinguished by the following: a pair of lateral scutae on abdomen (cf. Lin et al. 2009: fig. 5H, I) (vs. absent); the different shape of the embolus, the larger ratio of the bulbal length/width (1.49, Fig. 8, and cf. Lin et al. 2009: fig. 5A–C) (vs. 1.28), and the smaller length ratio of the embolus/bulb (0.36, Fig. 8, and cf. Lin et al. 2009: fig. 5A–C) (vs. 0.63); the distal part of the receptacle is seven times wider than the neck of the receptacle (cf. Lin et al. 2009: fig. 6F) (vs. five times).

##### Description.

See Lin et al. (2009).

##### Distribution

. Vietnam (Cuc Phuong National Park, site 3 in Fig. 32), known only from the type locality.

#### Pinelema cucurbitina

Taxon classificationAnimaliaAraneaeTelemidae

(Wang & Li, 2010) comb. nov.

C7BC6F9E-058D-58B9-9EA3-0108281099B7

Figures 8 , 32

Telema cucurbitina Wang and Li 2010b: 19, figs 16–19 (♂♀).

##### Type material.

Holotype: ♂ (IZCAS), China, Guangxi Zhuang Autonomous Region, Guilin Prefecture, Lingui County, Shuixian Cave, 25.2137N, 110.2008E, elevation ca. 161 m, 18.VII.2009, C. Wang and Z. Yao leg. Paratypes: 1♂ and 2♀ (IZCAS), same data as holotype. Examined.

##### Other material examined.

1♂ (molecular voucher, IZCAS), same data as holotype.

##### Diagnosis.

*Pinelema cucurbitina* comb. nov. resembles *P. spinafemora* comb. nov. but can be distinguished by the following: the bulb does not protrude ventro-distally (Fig. 8, and cf. Wang and Li 2010b: fig. 16C, D) (vs. protrude), the ventral bend between the embolus and the bulb is acute-angled (Fig. 8, and cf. Wang and Li 2010b: fig. 16C, D) (vs. right-angled).

##### Description.

See Wang and Li (2010b).

##### Distribution.

China (Guangxi, site 4 in Fig. 32).

#### Pinelema dongbei

Taxon classificationAnimaliaAraneaeTelemidae

(Wang & Ran, 1998) comb. nov.

12E49492-DA8A-515D-9595-6B7749DEA00D

Figures 8 , 32

Telema dongbei Wang and Ran 1998: 94, figs 1–5 (♂♀); Song et al. 1999: 51, fig. 21R–U (♂♀).

##### Type material.

Holotype: ♂ (AMNH), China, Guizhou Province, Qiannan Buyei and Miao Autonomous Prefecture, Libo County, Yuping Town, Dongbei Cave, 25.4804N, 107.8959E, elevation ca. 812 m, 13.IX.1996, J. Ran leg. Paratype: 1♀ (AMNH), same data as holotype. Not examined.

##### Other material examined.

1♂ and 2♀ (including molecular voucher, IZCAS), from the type locality, 18.III.2011, C. Wang and L. Lin leg.

##### Diagnosis.

*Pinelema dongbei* comb. nov. resembles *P. exiloculata* comb. nov. and *P. oculata* comb. nov. but can be distinguished by the following: eyes are absent (vs. vestigial); the embolus protrudes dorsally (Fig. 8, and cf. Wang and Ran 1998: figs 1, 2) (vs. not protruding); the receptacle is slightly swollen distally (cf. Wang and Ran 1998: fig. 5) (vs. distinctively swollen).

##### Description.

See Wang and Ran (1998).

##### Distribution.

China (Guizhou, site 5 in Fig. 32), known only from the type locality.

#### Pinelema exiloculata

Taxon classificationAnimaliaAraneaeTelemidae

(Lin, Pham & Li, 2009) comb. nov.

BE3FE699-D99B-5CA0-B88B-794C51FA7169

Figures 8 , 32

Telema exiloculata Lin et al. 2009: 332, figs 7A–F, 8A–G (♂♀).

##### Type material.

Holotype: ♂ (IZCAS), Vietnam, Hai Phong Province, Cat Ba National Park, Trung Trang Cave, 20.8000N, 106.9833E, elevation ca. 256 m, 16.VII.2008, S. Li leg. Paratypes: 1♂ and 2♀ (IZCAS), same data as holotype. Examined.

##### Other material examined.

1♂ (molecular voucher, IZCAS), same data as holotype.

##### Diagnosis.

*Pinelema exiloculata* comb. nov. resembles *P. dongbei* comb. nov. but can be distinguished by the following: vestigial eyes (vs. absent); the embolus does not protrude dorsally (Fig. 8, and cf. Lin et al. 2009: fig. 7B) (vs. protruding); the receptacle is distinctively swollen distally (cf. Lin et al. 2009: fig. 8D–G) (vs. swollen slightly).

##### Description.

See Lin et al. (2009).

##### Distribution.

Vietnam (Cat Ba National Park, site 6 in Fig. 32), known only from the type locality.

#### Pinelema grandidens

Taxon classificationAnimaliaAraneaeTelemidae

(Tong & Li, 2008) comb. nov.

1CDB9549-4C2F-5D95-94EF-DCBC1E54151A

Figures 8 , 32

Telema grandidens Tong and Li 2008b: 366, figs 1C, 4 (♂♀).

##### Type material.

Holotype: ♂ (IZCAS), China, Guizhou Province, Qiannan Buyei and Miao Autonomous Prefecture, Dushan County, Xiasi Town, Xinhe Village, Bayoudadong Cave, 25.4457N, 107.4316E, elevation ca. 929 m, 21.V.2005, Y. Tong and Y. Lin leg. Paratypes: 1♂ and 2♀ (IZCAS), same data as holotype. Examined.

##### Other material examined.

1♂ (molecular voucher, IZCAS), same data as holotype.

##### Diagnosis.

*Pinelema grandidens* comb. nov. resembles *P. oculata* comb. nov. but can be distinguished by the following: the eyes are absent (vs. present); the dorsal bend between the embolus and bulb is ca. 100° (Fig. 8, and cf. Tong and Li 2008b: fig. 4C) (vs. ca. 180°); the distal part of the receptacle is two times wider than the receptacle neck (cf. Tong and Li 2008b: fig. 4E) (vs. three times).

##### Description.

See Tong and Li (2008b).

##### Distribution.

China (Guizhou, site 7 in Fig. 32), known only from the type locality.

#### Pinelema laensis

Taxon classificationAnimaliaAraneaeTelemidae

Zhao & Li, 2018

FFC0ED09-B8E4-5ACD-8D88-FA18A06C24E3

Figures 8 , 32

Pinelema laensis Zhao et al. 2018a: 19, figs 7–9 (♂♀).

##### Type material.

Holotype: ♂ (IZCAS), Vietnam, Phu Tho Province, Tan Son District, Xuan Dai, Xuan Son National Park, La Cave, 21.1380N, 104.9390E, elevation ca. 424 m, 27.X.2012, H. Zhao and Z. Chen leg. Paratypes: 1♂ and 2♀ (IZCAS), same data as holotype. Examined.

##### Other material examined.

1♂ (molecular voucher, IZCAS), same data as holotype.

##### Diagnosis.

*Pinelema laensis* resembles *P. breviseta* comb. nov. but can be distinguished by the following: the bulb does not protrude ventro-distally (Fig. 8, and cf. Zhao et al. 2018a: fig. 7C, D) (vs. protrude), the embolus with a retrolateral vertical groove (Fig. 8, and cf. Zhao et al. 2018a: fig. 8B) (vs. embolus without any structure retrolaterally); the tip of receptacle is four times wider than the neck (cf. Zhao et al. 2018a: fig. 9C) (vs. three times).

##### Description.

See Zhao et al. (2018a).

##### Distribution.

Vietnam (Phu Tho, site 8 in Fig. 32), known only from the type locality.

#### Pinelema oculata

Taxon classificationAnimaliaAraneaeTelemidae

(Tong & Li, 2008) comb. nov.

6B6C3182-ABA9-5965-9B1E-0CBD30722E08

Figures 8 , 32

Telema oculata Tong and Li 2008b: 369, figs 1D, 5 (♀).

##### Type material.

Paratypes: 11 ♀ (IZCAS), China, Guizhou Province, Qiannan Buyei and Miao Autonomous Prefecture, Dushan County, Xiasi Town, Guojiafen Cave, 25.4833N, 107.4500E, 24.V.2005, Y. Tong and Y. Lin leg. Examined.

##### Other material examined.

1♂ and 2♀ (including one molecular voucher, IZCAS), same data as holotype.

##### Diagnosis.

*Pinelema oculata* comb. nov. resembles *P. grandidens* comb. nov. but can be distinguished by the following: the eyes are present (vs. absent); the dorsal bend between the embolus and bulb is ca. 180° (Fig. 8) (vs. ca. 100°); the distal part of the receptacle is three times wider than the receptacle neck (cf. Tong and Li 2008b: fig. 5C) (vs. two times).

##### Description.

See Tong and Li (2008b).

##### Distribution.

China (Guizhou, site 9 in Fig. 32), known only from the type locality.

#### Pinelema pedati

Taxon classificationAnimaliaAraneaeTelemidae

(Lin & Li, 2010) comb. nov.

6F6A5F7B-E700-5355-A497-C04EFA085983

Figures 8 , 32

Telema pedati Lin and Li 2010: 15, figs 8, 9 (♂♀).

##### Type material.

Holotype: ♂ (IZCAS), China, Guangxi Zhuang Autonomous Region, Hechi Prefecture, Nandan County, Chengguan Town, En Village, Encun Cave, 25.0693N, 107.6033E, elevation ca. 605 m, 3.III.2007, J. Liu and Y. Lin leg. Paratypes: 1♂ and 2♀ (IZCAS), same data as holotype. Examined.

##### Other material examined.

1♂ (molecular voucher, IZCAS), same data as holotype.

##### Diagnosis.

*Pinelema pedati* comb. nov. resembles *P. conglobare* comb. nov. but can be distinguished by the following: the smaller ratio of the bulbal length/width (1.60, Fig. 8, and cf. Lin and Li 2010: fig. 8A, B) (vs. 1.77), the ventral bend of the embolus and bulb is acute-angled (Fig. 8, and cf. Lin and Li 2010: fig. 8A, B) (vs. right-angled); the distal part of the receptacle is boot-shaped (cf. Lin and Li 2010: fig. 9B–D) (vs. globular).

##### Description.

See Lin and Li (2010).

##### Distribution.

China (Guangxi, site 10 in Fig. 32), known only from the type locality.

#### Pinelema spinafemora

Taxon classificationAnimaliaAraneaeTelemidae

(Lin & Li, 2010) comb. nov.

B4779359-85AB-5E31-8AD5-A3A4ADCF99A9

Figures 8 , 32

Telema spinafemora Lin and Li 2010: 18, figs 10, 11 (♂♀).

##### Type material.

Holotype: ♂ (IZCAS), China, Guangxi Zhuang Autonomous Region, Baise Prefecture, Lingyun County, Sicheng Town, Shuiyuan Cave, 24.3464N, 106.5579E, elevation ca. 573 m, 14.III.2007, J. Liu and Y. Lin leg. Paratypes: 1♂ and 2♀ (IZCAS), same data as holotype. Examined.

##### Other material examined.

1♂ (molecular voucher, IZCAS), same data as holotype.

##### Diagnosis.

*Pinelema spinafemora* comb. nov. resembles *P. cucurbitina* comb. nov. but can be distinguished by the following: the bulb protrudes ventro-distally (Fig. 8) (vs. not protrude), the ventral bend between the embolus and bulb is right-angled (Fig. 8) (vs. acute-angled).

##### Description.

See Lin and Li (2010).

##### Distribution.

China (Guangxi, site 11 in Fig. 32), known only from the type locality.

#### Pinelema xiezi

Taxon classificationAnimaliaAraneaeTelemidae

Zhao & Li, 2018

0A8EBCD7-E562-5E3B-8AE7-F3B4A9428A60

Figures 8 , 32

Pinelema xiezi Zhao et al. 2018a: 34, figs 16–18 (♂♀).

##### Type material.

Holotype: ♂ (IZCAS), Vietnam, Quang Binh Province, Phong Nha-Ke Bang National Park, Tien Son Cave, 17.5800N, 106.2820E, elevation ca. 102 m, 17.V.2016, Z. Chen and Q. Zhao leg. Paratypes: 1♂ and 2♀ (IZCAS), same data as holotype. Examined.

##### Other material examined.

1♂ (molecular voucher, IZCAS), same data as holotype.

##### Diagnosis.

*Pinelema xiezi* resembles *P. cucphongensis* comb. nov. but can be distinguished by the following: the lateral scuta on the abdomen is absent (cf. Zhao et al. 2018a: figs 16A, 17A) (vs. present); the different shape of the embolus, the smaller ratio of the bulbal length/width (1.28, Fig. 8, and cf. Zhao et al. 2018a: fig. 16C, D) (vs. 1.49), the larger length ratio of the embolus/bulb (0.63, Fig. 8, and cf. Zhao et al. 2018a: fig. 16C, D) (vs. 0.36); the distal part of the receptacle is five times wider than the neck of the receptacle (cf. Zhao et al. 2018a: fig. 18C) (vs. seven times).

##### Description.

See Zhao et al. (2018a).

##### Distribution.

Vietnam (Quang Binh, site 12 in Fig. 32), known only from the type locality.

#### Species group uncertain

Figures 9, 32

**Remarks.**

Six species: *Pinelema dengi* (Tong & Li, 2008) comb. nov., *P. mikrosphaira* (Wang & Li, 2010) comb. nov., *P. nuocnutensis* Zhao & Li, 2018, *P. spirulata* Zhao & Li, 2018, *P. tham* sp. nov., and *P. zhenzhuang* Zhao & Li, 2018 are not grouped and seem to represent groups of their own.

**Figure 9.**
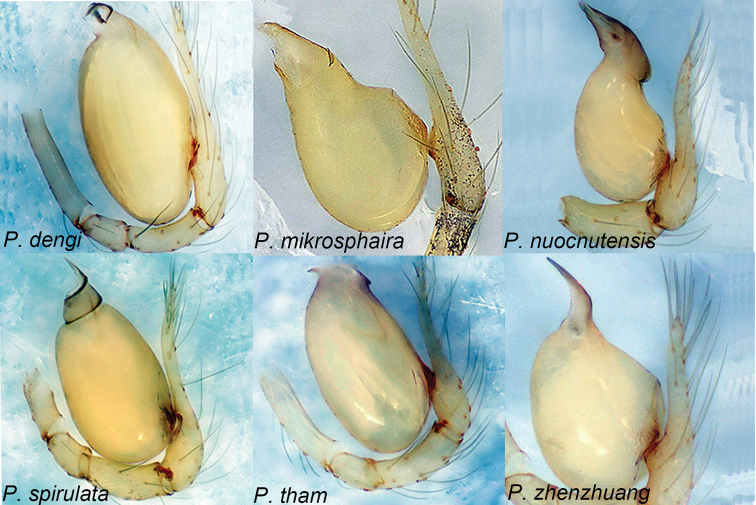
*Pinelema* spp., palp, retrolateral view. Six species not attached to a species group.

##### Pinelema dengi

Taxon classificationAnimaliaAraneaeTelemidae

(Tong & Li, 2008) comb. nov.

3842A246-F6EC-5280-8FC3-62BCE9BCD47B

Figures 9 , 32

Telema dengi Tong and Li 2008a: 69, figs 1C, 4A–H, 6 (♂♀); Tong 2013: 73, figs 31N, 88A–H (♂♀).

###### Type material.

Holotype: ♂ (IZCAS), China, Hainan Province, Sanya Prefecture, Lizhigou Town, Luobi Cave, 18.3318N, 109.5491E, elevation ca. 46 m, 10.IV.2005, X. Han, Y. Song, G. Deng and Y. Tong leg. Paratypes: 1♂ and 2♀ (IZCAS), same data as holotype. Examined.

###### Other material examined.

1♂ (molecular voucher, IZCAS), same data as holotype.

###### Diagnosis.

*Pinelema dengi* comb. nov. resembles *P. spirulata* but can be distinguished by the following: the cylindrical embolus (Fig. 9, and cf. Tong and Li 2008a: fig. 4B, C) (vs. twisted); the distal part of the receptacle is seven times wider than the receptacle neck (cf. Tong and Li 2008a: fig. 4D, E) (vs. five times).

###### Description.

See Tong and Li (2008a).

###### Distribution.

China (Hainan, site 13 in Fig. 32), known only from the type locality.

##### Pinelema mikrosphaira

Taxon classificationAnimaliaAraneaeTelemidae

(Wang & Li, 2010) comb. nov.

A402A1F8-A0E0-566C-81F9-48F6214D2F28

Figures 9 , 32

Telema mikrosphaira Wang and Li 2010b: 24, figs 20–23 (♂♀).

###### Type material.

Holotype: ♂ (IZCAS), China, Guangxi Zhuang Autonomous Region, Chongzuo Prefecture, Pinxiang County, Yinglong Cave, 22.1426N, 106.7128E, elevation ca. 200 m, 9.VIII.2009, C. Wang and Z. Yao leg. Paratypes: 1♂ and 2♀ (IZCAS), same data as holotype. Examined.

###### Other material examined.

1♂ (molecular voucher, IZCAS), same data as holotype.

###### Diagnosis.

*Pinelema mikrosphaira* comb. nov. resembles *P. nuocnutensis* but can be easily distinguished by the following: the eyes are encircled by black rings (vs. vestigial), the width ratio of the bulb/palpal tibia is 4.0 (Fig. 9) (vs. 2.0); the embolus is shaped like an equilateral-triangle (Fig. 9) (vs. beak-shaped); the distal part of the receptacle is swollen and globular (cf. Wang and Li 2010b: fig. 21C, D) (vs. not swollen).

###### Description.

See Wang and Li (2010b).

###### Distribution.

China (Guangxi, site 14 in Fig. 32), known only from the type locality.

##### Pinelema nuocnutensis

Taxon classificationAnimaliaAraneaeTelemidae

Zhao & Li, 2018

4D01D7E3-B6F2-5EDC-AD7F-933DCA46F225

Figures 9 , 32

Pinelema nuocnutensis Zhao et al. 2018a: 19, figs 4–6 (♂♀).

###### Type material.

Holotype: ♂ (IZCAS), Vietnam, Quang Binh Province, Phong Nha-Ke Bang National Park, Nuoc Nut Cave, 17.4940N, 106.2940E, elevation ca. 143 m, 25.V.2016, Z. Chen and Q. Zhao leg. Paratypes: 1♂ and 2♀ (IZCAS), same data as holotype. Examined.

###### Other material examined.

1♂ (molecular voucher, IZCAS), same data as holotype.

###### Diagnosis.

*Pinelema nuocnutensis* resembles *P. mikrosphaira* comb. nov. but can be distinguished by the following: the eyes are vestigial (vs. present), the width ratio of the bulb/palpal tibia is 2.0 (Fig. 9) (vs. 4.0); the beak-shaped embolus (Fig. 9) (vs. equilateral triangle); the distal part of the receptacle is not swollen (cf. Zhao et al. 2018a: fig. 6C) (vs. swollen).

###### Description.

See Zhao et al. (2018a).

###### Distribution.

Vietnam (Quang Binh, site 15 in Fig. 32), known only from the type locality.

##### Pinelema spirulata

Taxon classificationAnimaliaAraneaeTelemidae

Zhao & Li, 2018

AAEAF44E-0194-5DC3-B481-5272444CBF3B

Figures 9 , 32

Pinelema spirulata Zhao et al. 2018a: 30, figs 13–15 (♂♀).

###### Type material.

Holotype: ♂ (IZCAS), Vietnam, Phu Tho Province, Xuan Son National Park, Lap Cave, 21.1400N, 104.9430E, elevation ca. 403 m, 2.X.2012, H. Zhao and Z. Chen leg. Paratypes: 1♂ and 2♀ (IZCAS), same data as holotype. Examined.

###### Other material examined.

1♂ (molecular voucher, IZCAS), same data as holotype.

###### Diagnosis.

*Pinelema spirulata* resembles *P. dengi* comb. nov. but can be distinguished by the following: the embolus is twisted (Fig. 9) (vs. cylindrical); the distal part of the receptacle is five times wider than the receptacle neck (cf. Zhao et al. 2018a: fig. 15C) (vs. seven times).

###### Description.

See Zhao et al. (2018a).

###### Distribution.

Vietnam (Phu Tho, site 16 in Fig. 32), known only from the type locality.

##### Pinelema tham

Taxon classificationAnimaliaAraneaeTelemidae

Zhao & Li sp. nov.

739F8A41-B68E-5567-94BA-CF5B7C16A9B1

http://zoobank.org/AB6D01D9-B27D-410D-BAA1-C29DECA940BA

**Figure 10.**
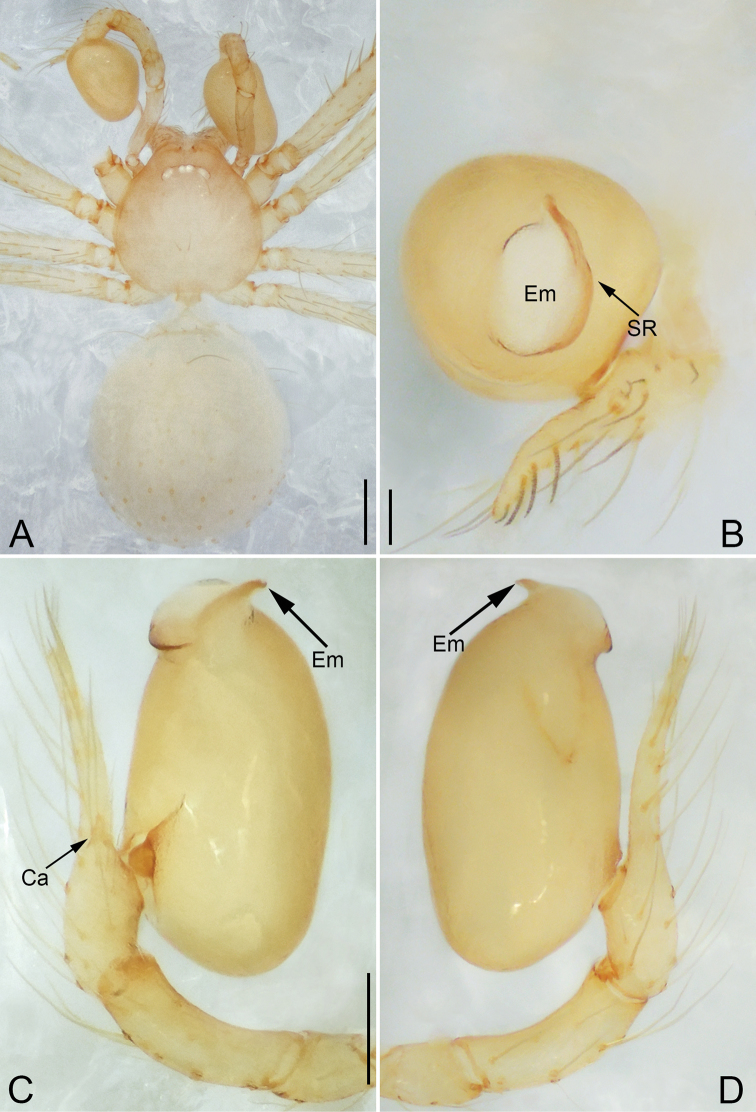
*Pinelema tham* sp. nov., male holotype. **A** Habitus, dorsal view **B** palp, ventral view **C** palp, prolateral view **D** palp, retrolateral view. Scale bars: 0.2 mm (**A**), 0.1 mm (**B–D**).

**Figure 11.**
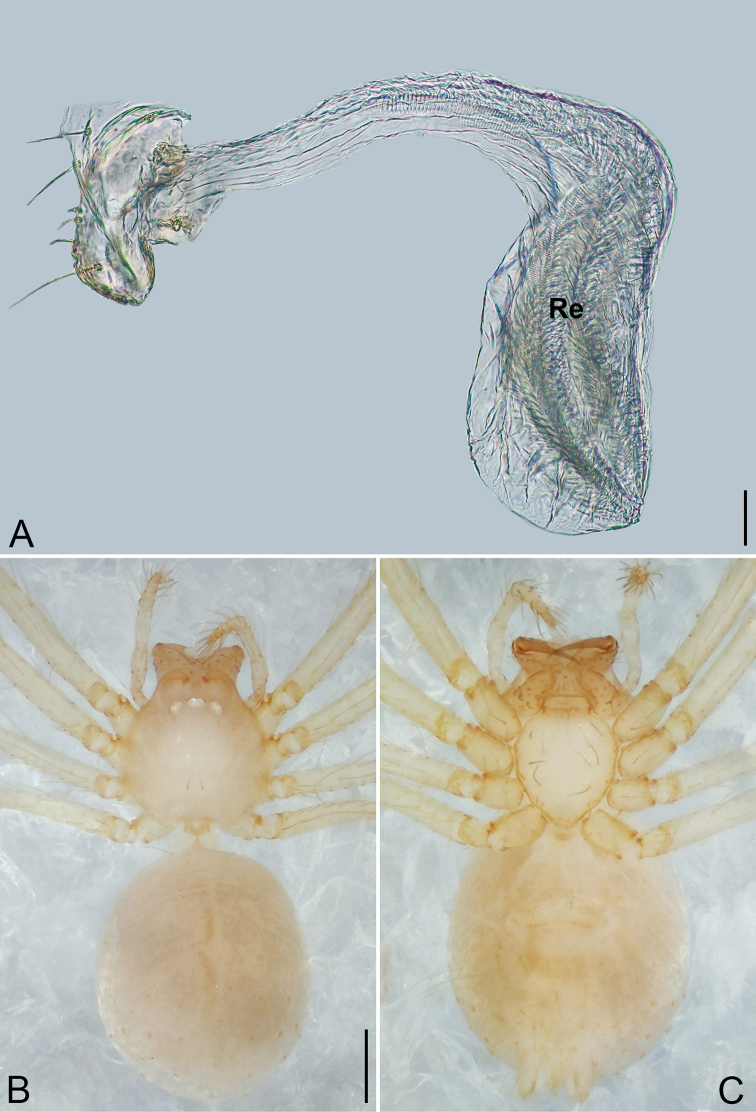
*Pinelema tham* sp. nov., female paratype. **A** Endogyne, lateral view **B** habitus, dorsal view **C** habitus, ventral view. Scale bars: 0.05 mm (**A**), 0.2 mm (**B, C**).

Figures 9 , 10 , 11 , 32

###### Type material.

Holotype: ♂ (IZCAS), Laos, Vien Tiane Province, Vang Vieng District, 1.54 km south of Vieng keo Village, Tham Cave, 18.9092N, 102.4421E, elevation ca. 270 m, XI.2012, S. Li and Z. Yao leg. Paratypes: 4♂ and 4♀ (IZCAS), same data as holotype.

###### Etymology.

The species name refers to the type locality; noun in apposition.

###### Diagnosis.

*Pinelema tham* sp. nov. resembles *P. zhenzhuang* but can be easily distinguished by the following: the embolus is hawk-beak-shaped (Figs 9, 10B) (vs. needle-shaped), the tip of the embolus is directed ventro-prolaterally (Figs 9, 10B) (vs. ventrally). The terminal part of the receptacle is 3.50 times wider than the neck (Fig. 11A) (vs. six times).

###### Description.

**Male (holotype).** Total length 1.23. Carapace 0.48 long, 0.47 wide. Abdomen 0.71 long, 0.59 wide. Carapace pale brown (Fig. 10A). Six vestigial eyes (Fig. 10A). Chelicerae, labium, and endites light brown, legs milky white (Fig. 10A). Sternum brown with sparse setae. Leg measurements: I 3.77 (1.08, 0.18, 1.19, 0.76, 0.56); II 3.20 (0.95, 0.19, 0.95, 0.60, 0.51); III 2.41 (0.75, 0.17, 0.63, 0.45, 0.41); IV 2.84 (0.89, 0.16, 0.79, 0.55, 0.45). Abdomen grey (Fig. 10A).

Palp. Tibia 1.96 times longer than patella, cymbium 1.67 times longer than tibia, 1.45 times longer than femur, cymbial apophysis short, as long as half width of cymbium base (Fig. 10C); bulb bean shaped (Fig. 10C, D); spiral ridge brown; embolus short relative to bulb, tip well sclerotized, hawk-beak-shaped, directed ventro-prolaterally (Fig. 10B–D).

**Female.** Total length 1.24. Carapace 0.44 long, 0.43 wide. Abdomen 0.74 long, 0.60 wide. Coloration as in male (Fig. 11B, C). Leg measurements: I 3.31 (0.93, 0.18, 1.03, 0.63, 0.55); II 2.79 (0.82, 0.17, 0.82, 0.51, 0.47); III 2.18 (0.67, 0.16, 0.57, 0.40, 0.38); IV 2.55 (0.79, 0.16, 0.71, 0.49, 0.40). Abdomen pale brown. Receptacle with two membranous tubes, swollen distally (Fig. 11A), tip of receptacle four times wider than the neck (Fig. 11A).

###### Distribution.

Laos (Vien Tiane, site 17 in Fig. 32), known only from the type locality.

##### Pinelema zhenzhuang

Taxon classificationAnimaliaAraneaeTelemidae

Zhao & Li, 2018

EDA559C0-633F-512F-860E-4F2F5DE9D370

Figures 9 , 32

Pinelema zhenzhuang Zhao et al. 2018a: 35, figs 19–22 (♂♀).

###### Type material.

Holotype: ♂ (IZCAS), Vietnam, Quang Binh Province, Phong Nha-Ke Bang National Park, Tien Duong Cave, 17.5190N, 106.2230E, elevation ca. 133 m, 18.V.2016, Z. Chen and Q. Zhao leg. Paratypes: 1♂ and 2♀ (IZCAS), same data as holotype. Examined.

###### Other material examined.

1♂ (molecular voucher, IZCAS), same data as holotype.

###### Diagnosis.

*Pinelema zhenzhuang* resembles *P. tham* sp. nov. but can be distinguished by the following: the embolus is needle shaped (cf. Zhao et al. 2018a: fig. 19C, D) (vs. hawk beak shaped), the tip of the embolus is directed ventrally (Fig. 9) (vs. ventro-prolaterally). The terminal part of the receptacle is six times wider than the neck (cf. Zhao et al. 2018a: fig. 21C) (vs. 3.50 times).

###### Description.

See Zhao et al. (2018a).

###### Distribution.

Vietnam (Quang Binh, site 18 in Fig. 32), known only from the type locality.

#### Genus Apneumonella

Taxon classificationAnimaliaAraneaeTelemidae

Fage, 1921

CBBFACBA-0BD6-51CB-A964-7DAC296A4B24

Apneumonella Fage, 1921: 620; Brignoli 1978: 113; Song et al. 2017: 14.

##### Type species.

*Apneumonella oculata* Fage, 1921 from Tanzania, Africa; Simon and Fage 1922: 528, fig. II.

##### Comments.

This genus currently includes three species: two from Africa and one from Sumatra. The Sumatran species, *A. jacobsoni* Brignoli, 1977, is known by the female only and is most likely misplaced in this genus (see comments below).

#### Apneumonella jacobsoni

Taxon classificationAnimaliaAraneaeTelemidae

Brignoli, 1977

936E11F5-1883-5F22-B96E-01875D33FB2B

Figure 1F

Apneumonella jacobsoni Brignoli, 1977: 221, figs 1–6 (♀); Lehtinen 1986: 155, fig. 6 (♀).

##### Type material.

Holotype: ♀ (RMNH), Indonesia, Sumatra, West Sumatra Province, Fort de Kock, 0.2484S, 100.4832E, elevation ca. 920 m, 1926, E. Jacobson leg. Not examined.

##### Other material examined.

3♀ (including molecular voucher, IZCAS) from the type locality: I. 2014, H. Zhao leg.

##### Distribution.

Indonesia (Sumatra, West Sumatra, site 1 in Fig. 33).

##### Comments.

The placement of this species in *Apneumonella* is doubtful because the males of both *A. oculata* Fage, 1921 (the type species of *Apneumonella*) and *A. jacobsoni* are unknown, and females of the above two species provide little information regarding their generic belonging. To test the relationship of *A. jacobsoni* to *A. oculata*, molecular data of *A. oculata* is necessary.

#### Genus Mekonglema

Taxon classificationAnimaliaAraneaeTelemidae

Zhao & Li gen. nov.

FEF9BB8A-A08B-5A48-AC91-7958DCFCD58B

http://zoobank.org/119C8ACC-75B3-4422-9FBB-99ED8C2B52F0

##### Type species.

*Mekonglema bailang* sp. nov. from Yunnan, China.

##### Etymology.

The generic name is a combination of “Mekong” referring to the Mekong-Lancang River which encompasses the distributional range of the genus, and “-lema”, a convention used because it is part of the genus *Telema*, which was the first genus described in Telemidae. The gender is feminine.

##### Diagnosis.

**Figure 12.**
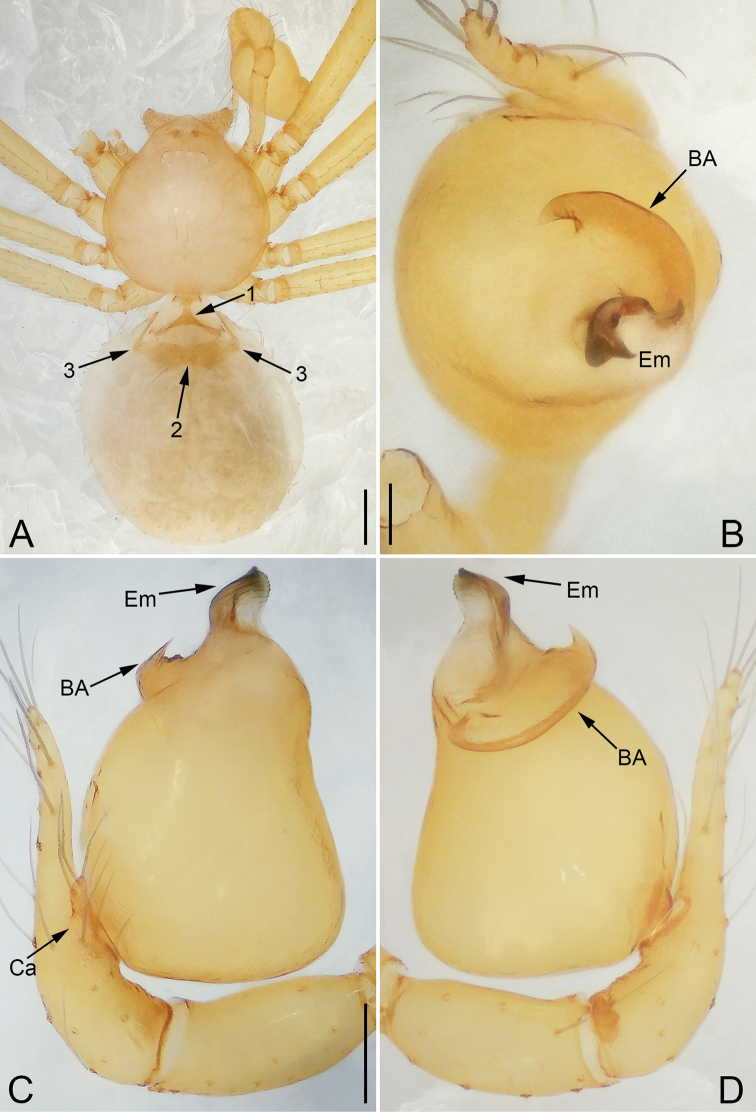
*Mekonglema bailang* sp. nov., male holotype **A** Habitus, dorsal view **B** embolus, apical view **C** palp, prolateral view **D** palp, retrolateral view. Scale bars: 0.2 mm (**A**), 0.05 mm (**B**), 0.1 mm (**C, D**).

**Figure 13.**
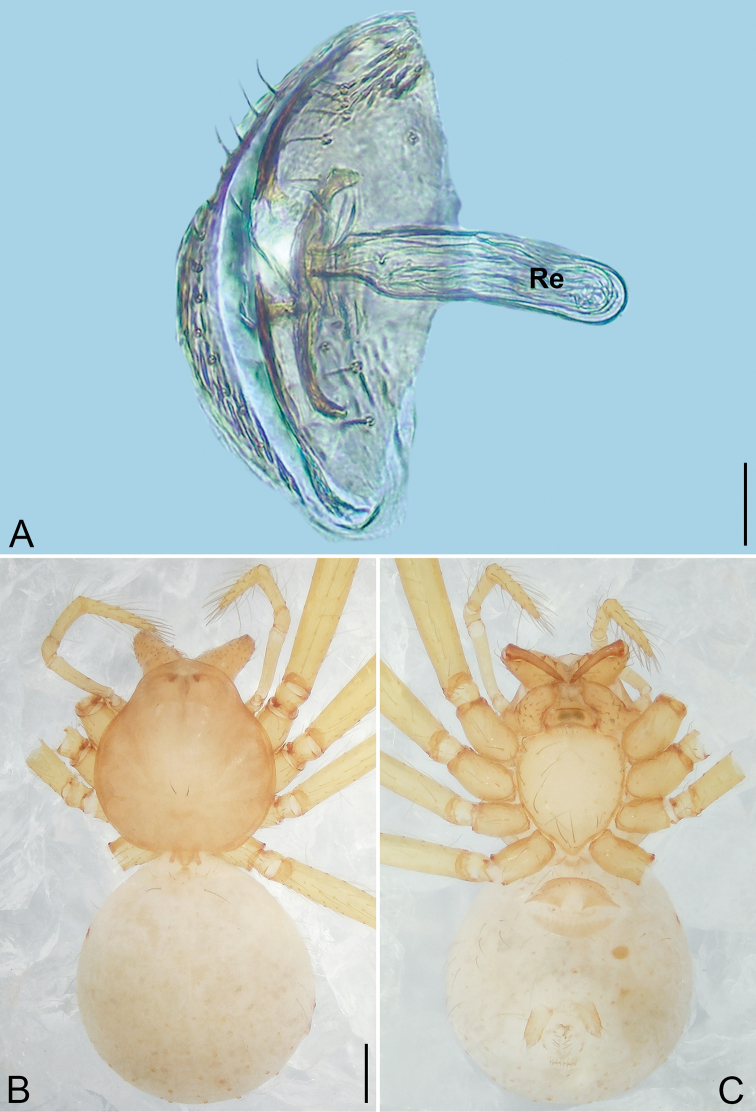
*Mekonglema bailang* sp. nov., female paratype **A** Endogyne, lateral view **B** habitus, dorsal view **C** habitus, ventral view. Scale bars: 0.05 mm (**A**), 0.2 mm (**B, C**).

**Figure 14.**
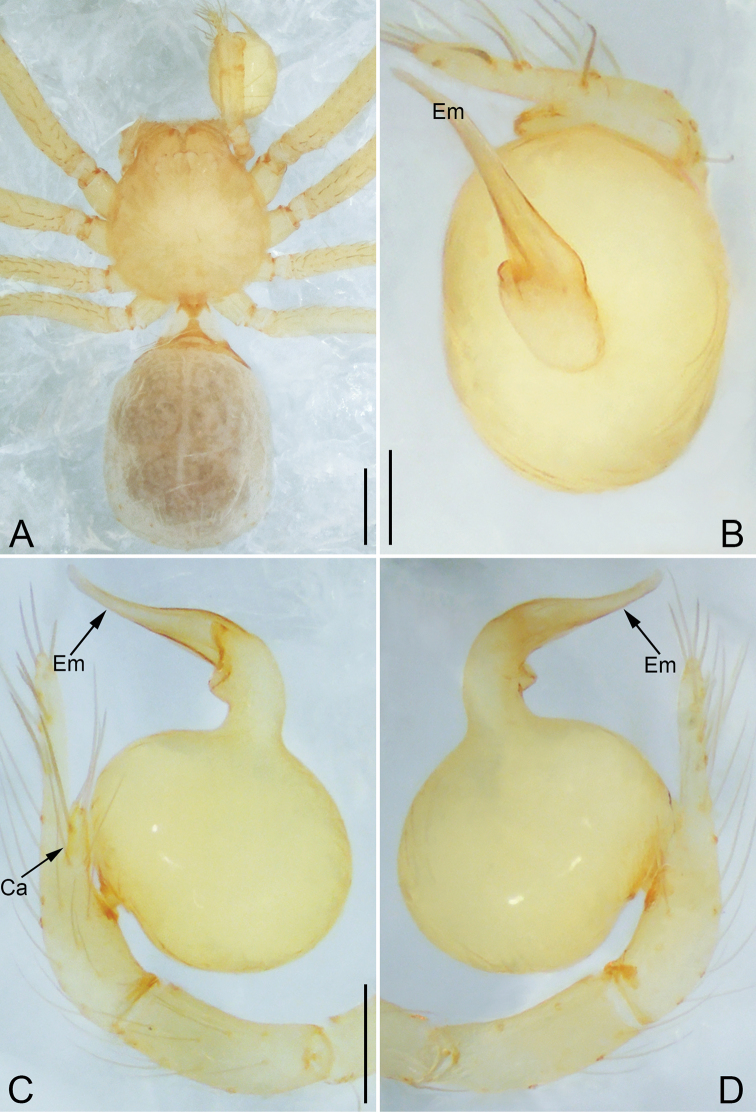
*Mekonglema kaorao* sp. nov., male holotype **A** Habitus, dorsal view **B** embolus, apical view **C** palp, prolateral view **D** palp, retrolateral view. Scale bars: 0.2 mm (**A**), 0.05 mm (**B**), 0.1 mm (**C, D**).

*Mekonglema* gen. nov. resembles *Pinelema* but can be distinguished by the following: Males of *Mekonglema* gen. nov. have a bulbal apophysis (*M. bailang* sp. nov., *M. xinpingi* comb. nov., and *M. yan* sp. nov.) (Fig. 12D) (vs. absent), or the tip of the embolus is directed dorsally (*M. kaorao* sp. nov. and *M. walayaku* sp. nov.) (Figs 14C, D, 16C, D) (vs. tip of embolus directed ventrally). Females of *Mekonglema* gen. nov. can be distinguished from those of *Pinelema* by the receptacle lacking tubes inside (Fig. 13A) (vs. receptacle with several membranous tubes inside).

##### Description.

Total length: 1.06–1.50 (male), 1.15–1.70 (female). Carapace without pattern in troglobitic species or with radial striae in rainforest species, i.e. *M. xinpingi* comb. nov. Sternum with sparse setae, milky white or light brown in troglobitic species, or nearly black in *M. xinpingi* comb. nov. Eyes ringed with black, vestigial, or absent; Leg formula: 1-2-4-3, tibial glands belt-shaped (Fig. 1C). Abdomen of males with three different types of scutae (except in *M. yan* sp. nov.): the first scuta connects to pedicel, dorsal (arrow 1 on Fig. 12A); the second scuta is posterior to the first one, dorsal (arrow 2 on Fig. 12A); and the third scutae are paired, lateral (arrows 3 in Fig. 12A). Abdomen of females without scuta. Male palp: cymbial apophysis cone shaped and located medially (Fig. 12C); bulb ellipsoid or nearly ellipsoid, bulbal apophysis present (except *M. kaorao* sp. nov. and *M. walayaku* sp. nov.); embolus short relative to cymbium, sclerotized, directed outward from the cymbium in *M. bailang* sp. nov., *M. xinpingi* comb. nov., and *M. yan* sp. nov.; embolus long relative to cymbium, unsclerotized, directed toward cymbium in *M. kaorao* sp. nov. and *M. walayaku* sp. nov. Endogyne: composed of single tube-like or globular receptacle with short neck, membranous tubes absent.

##### Composition.

*Mekonglema bailang* sp. nov., *M. kaorao* sp. nov., *M. walayaku* sp. nov., *M. xinpingi* comb. nov., and *M. yan* sp. nov. (Fig. 34).

##### Distribution.

China (Yunnan) and Laos (Luang Prabang) (sites 2–6 in Fig. 33).

#### Mekonglema bailang

Taxon classificationAnimaliaAraneaeTelemidae

Zhao & Li sp. nov.

8F32EB07-629D-5B02-AED7-5BF00650BC2B

http://zoobank.org/83DB76B2-BC6C-44FE-9D93-9DDB2B5CBB40

Figures 12 , 13 , 33

##### Type material.

Holotype: ♂ (IZCAS), China, Yunnan Province, Baoshan Prefecture, Shidian County, Bailang Town, Xianren Cave, 24.6536N, 99.2645E, elevation ca. 1987 m, 29.VII.2010, C. Wang and Q. Zhao leg. Paratypes: 5♂ and 1♀ (IZCAS), same data as holotype.

##### Etymology.

The species name refers to the type locality; noun in apposition.

##### Diagnosis.

This species resembles *M. yan* sp. nov. but can be distinguished by the following characters: abdominal scutae present in the male (vs. absent); the embolus is sclerotized (Fig. 12B–D) (vs. unsclerotized), the bulb length/width ratio is ca. 1.2 (Fig. 12C, D) (vs. ca. 1.7); the receptacle is short and almost straight (Fig. 13A) (vs. long and U-shaped). This species also resembles *M. xinpingi* comb. nov. but can be differentiated by the following: the absence of eyes (vs. presence), the nearly ellipsoid shape of the bulb (Fig. 12C, D) (vs. droplet shaped), the fin-like embolus (Fig. 12C) (vs. cone shaped), and the receptacle is short and not swollen distally (vs. receptacle long and swollen distally).

##### Description.

**Male (holotype).** Total length 1.50. Carapace 0.65 long, 0.61 wide. Abdomen 0.85 long, 0.79 wide. Carapace brown (Fig. 12A). Four vestigial eyes (Fig. 12A). Chelicerae, legs, labium, and endites light brown. Sternum bright brown with sparse setae (Fig. 12A). Leg measurements: I 5.05 (1.45, 0.23, 1.60, 1.13, 0.64); II 4.54 (1.36, 0.23, 1.41, 0.95, 0.59); III 3.20 (0.98, 0.21, 0.92, 0.62, 0.47); IV 4.17 (1.30, 0.20, 1.24, 0.88, 0.55). Abdomen pale brown (Fig. 12A).

Palp. Tibia 2.12 times longer than patella, cymbium 1.74 times longer than tibia, cymbial apophysis length 2/3 as wide as cymbial base (Fig. 12C); bulb shaped as shown in Fig. 12C, D; bulbal apophysis sclerotized and semi-circular with claw-like tip, embolus sclerotized (Fig. 12B–D).

**Female.** Total length 1.48. Carapace 0.65 long, 0.63 wide. Abdomen 0.81 long, 0.80 wide. Coloration as in male (Fig. 13B, C). Leg measurements: I 4.78 (1.47, 0.24, 1.45, 1.00, 0.62); II 4.35 (1.34, 0.22, 1.33, 0.88, 0.58); III 3.04 (0.96, 0.22, 0.87, 0.54, 0.45); IV 4.05 (1.28, 0.19, 1.18, 0.85, 0.55). Abdomen pale grey. Insemination entrance membranous, two times thinner than receptacle (Fig. 13A); receptacle tube-like and straight (Fig. 13A).

##### Distribution.

China (Yunnan, Baoshan, site 2 in Fig. 33), known only from the type locality.

#### Mekonglema kaorao

Taxon classificationAnimaliaAraneaeTelemidae

Zhao & Li sp. nov.

A3DD00AF-26E2-59A0-9935-E196ED44AD36

http://zoobank.org/F2C24399-518A-4CDB-B1DF-3EEF3EEC1E99

**Figure 15.**
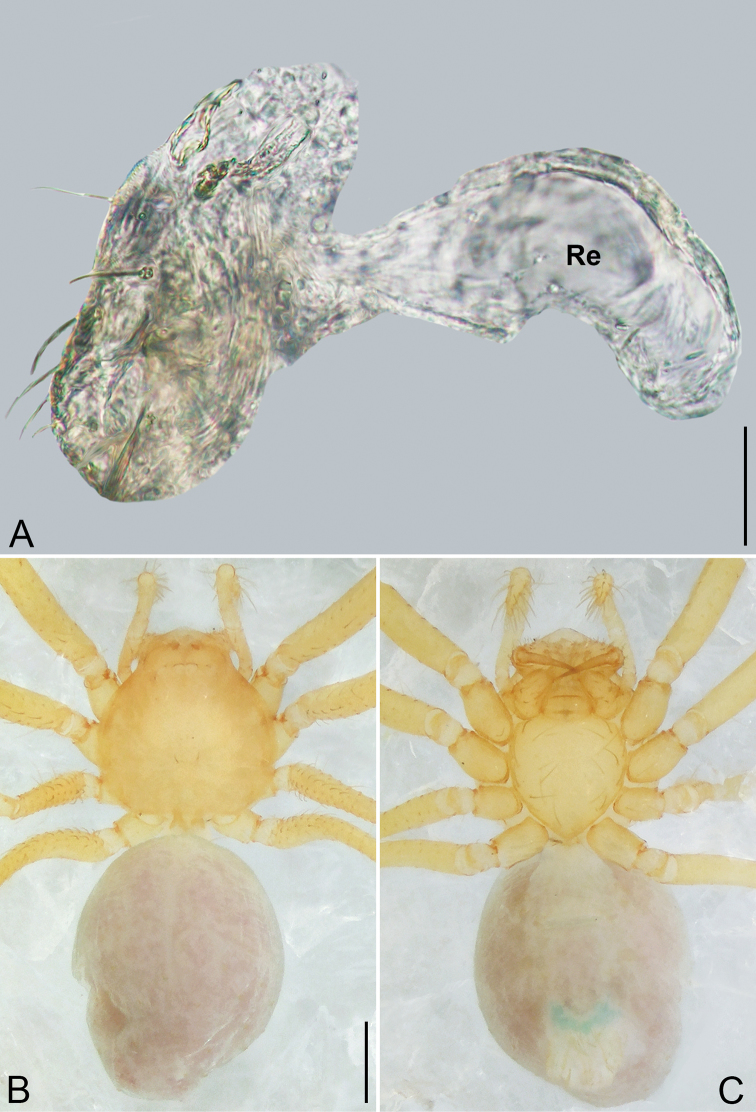
*Mekonglema kaorao* sp. nov., female paratype **A** Endogyne, lateral view **B** habitus, dorsal view **C** habitus, ventral view. Scale bars: 0.05 mm (**A**), 0.2 mm (**B, C**).

Figures 14 , 15 , 33

##### Type material.

Holotype: ♂ (SMF), Laos, Luang Prabang Province, Vieng Phoukha town, Ban Nam Eng, Kao Rao Cave, 20.7251N, 101.1541E, elevation ca. 729 m, P. Jäger leg. Paratype: 1♀ (SMF), same data as holotype.

##### Etymology.

The species name refers to the type locality; noun in apposition.

##### Diagnosis.

This species resembles *M. walayaku* sp. nov. but can be distinguished by the following characters: the bulb is nearly globular (Fig. 14C, D) (vs. ellipsoidal), the tip of the embolus is not sclerotized (Fig. 14B) (vs. well sclerotized). The tip of receptacle is 1.50 times wider than the neck (Fig. 15A) (vs. three times).

##### Description.

**Male (holotype).** Total length 1.13. Carapace 0.49 long, 0.44 wide. Abdomen 0.58 long, 0.45 wide. Carapace brown (Fig. 14A). Six vestigial eyes (Fig. 14A). Chelicerae, legs, labium, and endites light brown. Sternum bright brown with sparse setae. Leg measurements: I 3.06 (0.80, 0.18, 0.98, 0.62, 0.48); II 2.84 (0.85, 0.17, 0.84, 0.53, 0.45); III 2.06 (0.63, 0.15, 0.56, 0.37, 0.35); IV 2.73 (0.81, 0.15, 0.81, 0.56, 0.40). Abdomen light brown (Fig. 14A).

Palp. Tibia 2.71 times longer than patella, cymbium 1.65 times longer than tibia, length of cymbial apophysis as wide as cymbial base (Fig. 14C); bulb nearly globular (Fig. 14C, D); embolus 2/3 as long as cymbium, directed dorsally (Fig. 14C, D).

**Female.** Total length 1.18. Carapace 0.46 long, 0.45 wide. Abdomen 0.65 long, 0.52 wide. Eyes vestigial (Fig. 15B). Coloration as in male (Fig. 15B, C). Leg measurements: I 2.95 (0.88, 0.18, 0.88, 0.56, 0.45); II 2.62 (0.79, 0.17, 0.75, 0.50, 0.41); III 1.92 (0.59, 0.14, 0.53, 0.34, 0.32); IV 2.62 (0.79, 0.15, 0.75, 0.53, 0.40). Abdomen grey. Receptacle membranous, without tubes inside, neck 1.50 times thinner than tip (Fig. 15A).

##### Distribution.

Laos (Luang Prabang, site 3 in Fig. 33), known only from the type locality.

#### Mekonglema walayaku

Taxon classificationAnimaliaAraneaeTelemidae

Zhao & Li sp. nov.

4286A8EA-9C6B-5338-BA87-846182BDDDEE

http://zoobank.org/5F673823-850D-4F65-B0AA-47094A9AAB16

**Figure 16.**
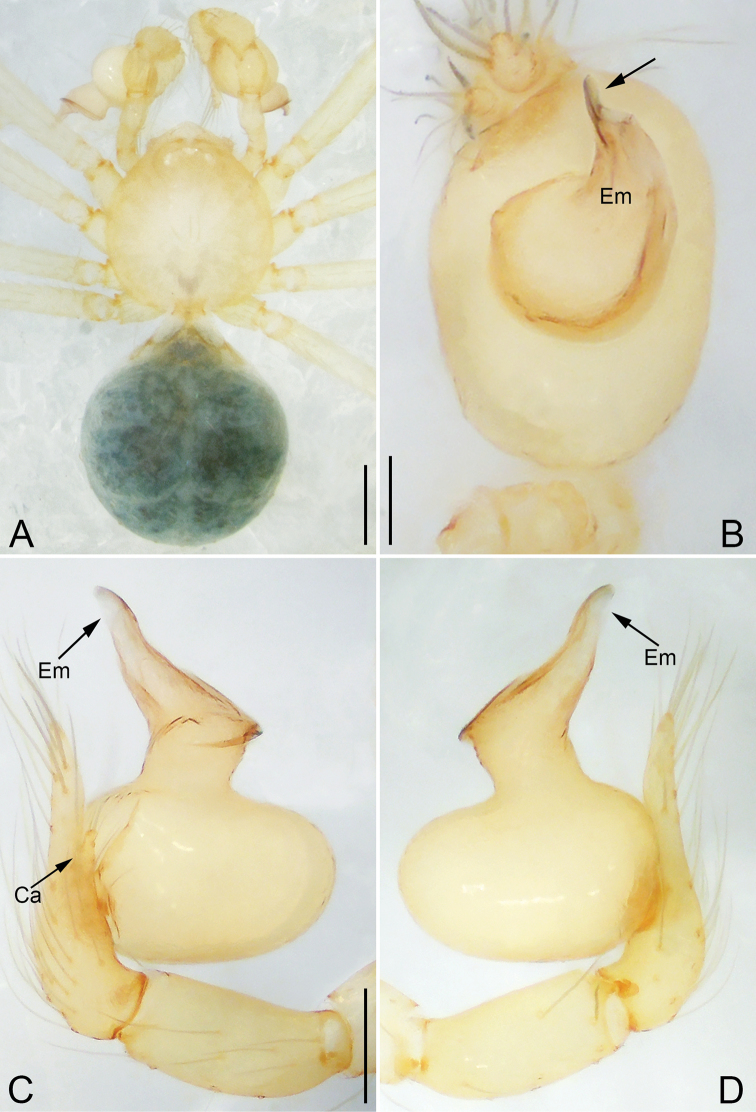
*Mekonglema walayaku* sp. nov., male holotype **A** Habitus, dorsal view **B** embolus, apical view **C** palp, prolateral view **D** palp, retrolateral view. Scale bars: 0.2 mm (**A**), 0.05 mm (**B**), 0.1 mm (**C, D**).

**Figure 17.**
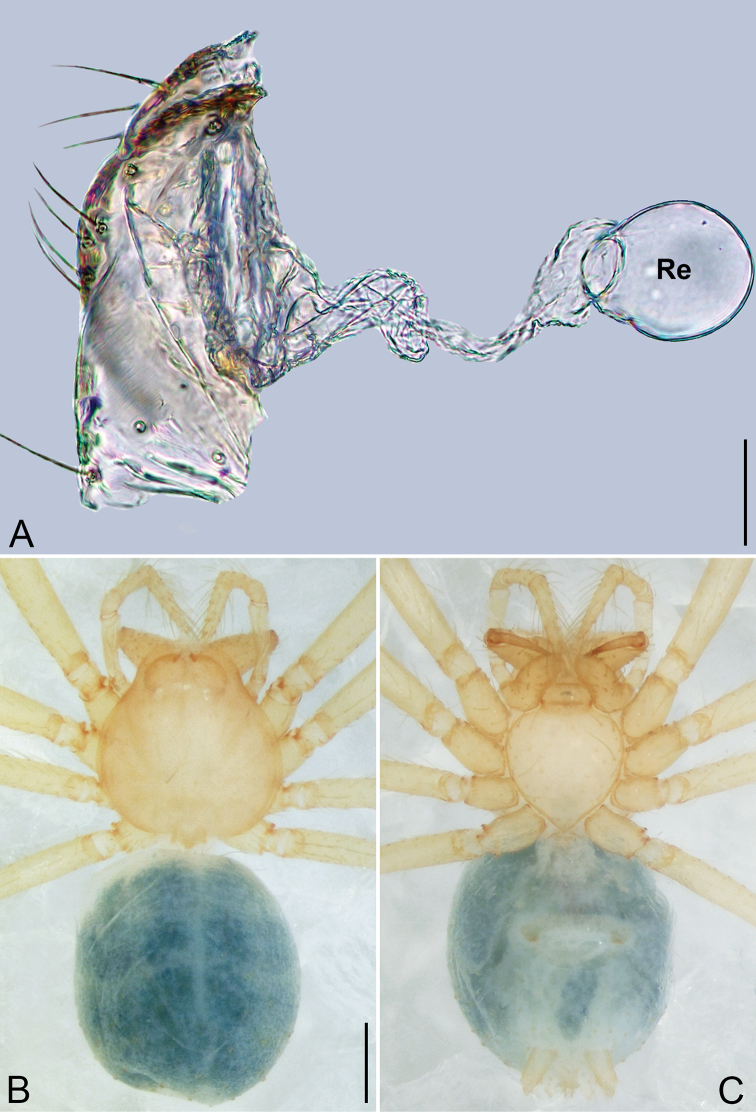
*Mekonglema walayaku* sp. nov., female paratype **A** Endogyne, lateral view **B** habitus, dorsal view **C** habitus, ventral view. Scale bars: 0.05 mm (**A**), 0.2 mm (**B, C**).

Figures 16 , 17 , 33

##### Type material.

Holotype: ♂ (IZCAS), China, Yunnan Province, Nujiang Lisu Autonomous Prefecture, Lushui County, Daxingdi Township, Walayaku Cave. 26.1215N, 98.8581E, elevation ca. 895 m, 24.VI.2016, Y. Li and J. Liu leg. Paratypes: 2♂ and 4♀ (IZCAS), same data as holotype.

##### Etymology.

The species name refers to the type locality; noun in apposition.

##### Diagnosis.

This species resembles *M. kaorao* sp. nov. but can be distinguished by the following characters: the bulb is ellipsoidal (Fig. 16C, D) (vs. nearly globular), the tip of the embolus is well sclerotized (arrow on Fig. 16B) (vs. unsclerotized); the neck of the receptacle is ca. three times thinner (vs. 1.50 times thinner) than the distal part of the receptacle (Fig. 17A).

##### Description.

**Male (holotype).** Total length 1.06. Carapace 0.49 long, 0.43 wide. Abdomen 0.56 long, 0.55 wide. Carapace light brown (Fig. 16A). Eyes vestigial (Fig. 16A). Chelicerae, legs, labium, and endites light yellow. Sternum bright brown with sparse setae. Leg measurements: I 3.65 (1.03, 0.18, 1.13, 0.78, 0.53); II 3.20 (0.96, 0.17, 0.95, 0.65, 0.47); III 2.26 (0.67, 0.16, 0.61, 0.44, 0.38); IV 2.86 (0.85, 0.16, 0.83, 0.59, 0.43). Abdomen dark blue (Fig. 16A).

Palp: Tibia 1.88 times longer than patella, cymbium 1.34 times longer than tibia, cymbial apophysis length as wide as cymbial base (Fig. 16C); bulb ellipsoidal as in Fig. 16C, D; embolus 5/7 as long as cymbium, directed dorsally (Fig. 16C, D).

**Female.** Total length 1.15. Carapace 0.50 long, 0.47 wide. Abdomen 0.63 long, 0.58 wide. Coloration as in male (Fig. 17B, C). Leg measurements: I 3.36 (0.97, 0.19, 1.03, 0.69, 0.48); II 3.00 (0.89, 0.19, 0.88, 0.59, 0.45); III 2.15 (0.65, 0.17, 0.59, 0.40, 0.34); IV 2.80 (0.85, 0.18, 0.79, 0.56, 0.42). Abdomen blue. Receptacle membranous, neck much longer than tip, and tip three times wider than neck (Fig. 17A).

##### Distribution.

China (Yunnan, Nujiang, site 4 in Fig. 33), known only from the type locality.

#### Mekonglema xinpingi

Taxon classificationAnimaliaAraneaeTelemidae

(Lin & Li, 2008) comb. nov.

05CD52D1-16D6-5838-A3CE-2A431D72A1A9

Figure 1C

Seychellia xinpingi Lin and Li 2008: 650, figs 1–9 (♂♀).

##### Type material.

Paratypes: 1♂ and 1♀ (IZCAS), China, Yunnan Province, Xishuangbanna Autonomous Prefecture, Mengla County, Menglun Town, rainforest, leaf litter, 21.9001N, 101.1833E, V–VII.2005, G. Zheng leg. Examined.

##### Other material examined.

3♀ (including one molecular voucher, IZCAS), same data as paratypes.

##### Diagnosis.

*Mekonglema xinpingi* comb. nov. resembles *M. bailang* sp. nov. but can be distinguished by the following characters: eyes are present (vs. absent); carapace with distinct radial striae (vs. no pattern); bulb is droplet-shaped (cf. Lin and Li 2008: fig. 2) (vs. nearly ellipsoidal).

##### Description.

See Lin and Li (2008).

##### Distribution.

China (Yunnan, Xishuangbanna, site 5 in Fig. 33), known only from the type locality.

##### Comments.

This species is transferred to *Mekonglema* gen. nov. because it shares a similar shape of the copulatory organs with *M. bailang* sp. nov., the type species of the genus. This placement is also supported by molecular phylogenetic analyses (Fig. 34).

#### Mekonglema yan

Taxon classificationAnimaliaAraneaeTelemidae

Zhao & Li sp. nov.

B697FFA5-8200-53CF-8A7F-2FDB97EE7A65

http://zoobank.org/B8F48215-808E-47D3-956F-EFD83BE32B58

**Figure 18.**
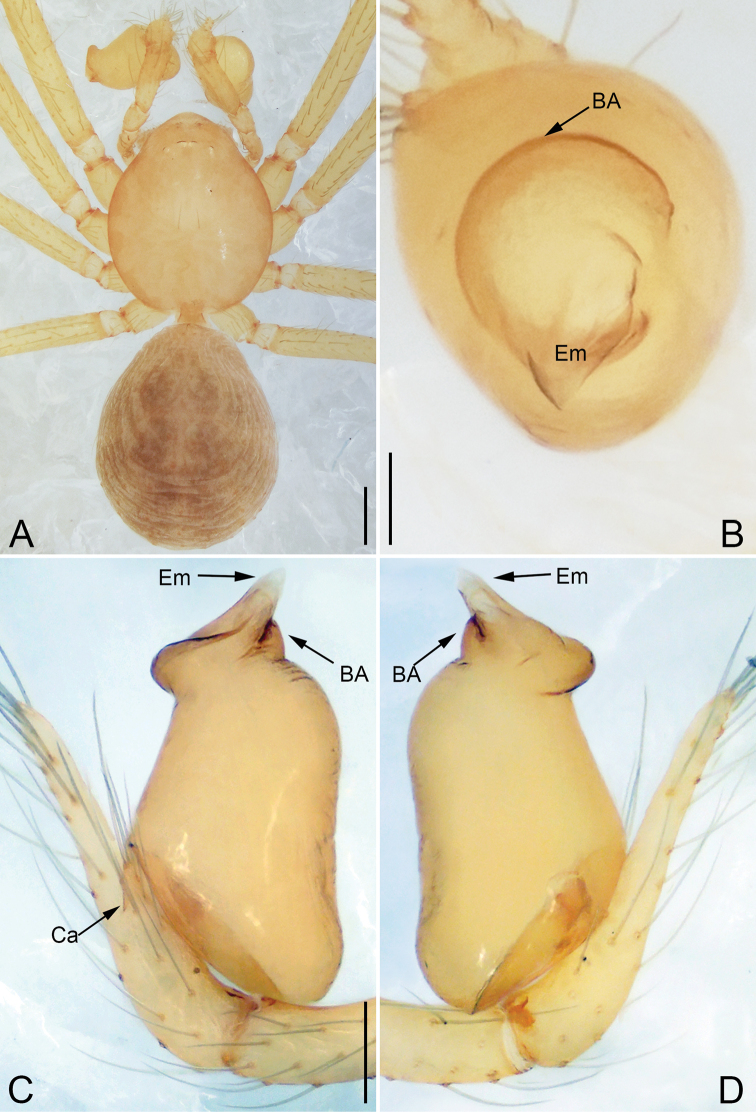
*Mekonglema yan* sp. nov., male holotype **A** Habitus, dorsal view **B** embolus, apical view **C** palp, prolateral view **D** palp, retrolateral view. Scale bars: 0.2 mm (**A**), 0.05 mm (**B**), 0.1 mm (**C, D**).

**Figure 19.**
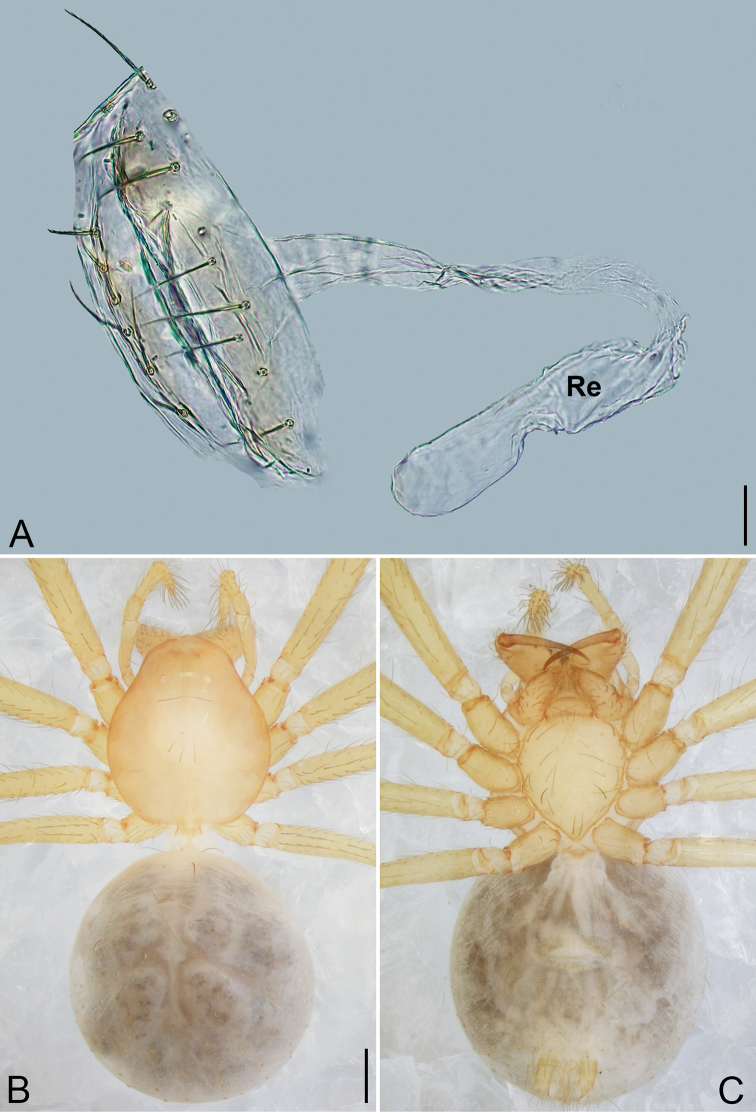
*Mekonglema yan* sp. nov., female paratype **A** Endogyne, lateral view **B** habitus, dorsal view **C** habitus, ventral view. Scale bars: 0.05 mm (**A**), 0.2 mm (**B, C**).

Figures 18 , 19 , 33

##### Type material.

Holotype: ♂ (IZCAS), China, Yunnan Province, Baoshan Prefecture, Tengchong County, Diantan Town, Lianzu Village, Yan Cave, 25.5501N, 98.4452E, elevation ca. 1867 m, 27.XI.2013, Y. Li and J. Liu leg. Paratypes: 2♂ and 4♀ (IZCAS), same data as holotype.

##### Etymology.

The species refers to the type locality; noun in apposition.

##### Diagnosis.

*Mekonglema yan* sp. nov. resembles *M. bailang* sp. nov. but can be distinguished by the following characters: an abdominal scuta is absent in the male (vs. present); the tip of the embolus is unsclerotized (Fig. 18B–D) (vs. sclerotized), the bulbal length/width ratio is approximately 1.7 (Fig. 18C, D) (vs. 1.2); the receptacle is long and U-shaped (Fig. 19A) (vs. short and nearly straight).

##### Description.

**Male (holotype).** Total length 1.48. Carapace 0.69 long, 0.57 wide. Abdomen 0.77 long, 0.63 wide. Carapace brown (Fig. 18A). Four vestigial eyes (Fig. 18A). Chelicerae, labium, and endites brown, legs yellow. Sternum bright brown with sparse setae. Leg measurements: I 4.86 (1.44, 0.23, 1.52, 1.00, 0.67); II 4.33 (1.34, 0.23, 1.25, 0.91, 0.60); III 3.20 (1.00, 0.21, 0.88, 0.61, 0.50); IV 4.04 (1.25, 0.20, 1.16, 0.87, 0.56). Abdomen light brown (Fig. 18A).

Palp: Tibia 2.05 times longer than patella, cymbium 1.73 times longer than tibia, length of cymbial apophysis as wide as cymbial base (Fig. 18C); bulb shaped as in Fig. 18C, D; bulbal apophysis sclerotized and looped about 270° (Fig. 18B–D); embolus membranous and finger-like (Fig. 18C, D).

**Female**: Total length 1.70. Carapace 0.71 long, 0.61 wide. Abdomen 0.97 long, 0.90 wide. Coloration lighter than in male (Fig. 19B, C). Leg measurements: I 4.72 (1.44, 0.23, 1.45, 0.95, 0.65); II 4.24 (1.30, 0.23, 1.25, 0.85, 0.61); III 3.49 (1.33, 0.20, 0.89, 0.59, 0.48); IV 4.05 (1.25, 0.20, 1.19, 0.85, 0.56). Abdomen grey. Receptacle membranous, U-shaped, neck as wide as distal part (Fig. 19A).

##### Distribution.

China (Yunnan, Baoshan, site 6 in Fig. 33), known only from the type locality.

#### Genus Siamlema

Taxon classificationAnimaliaAraneaeTelemidae

Zhao & Li gen. nov.

EC9EC00E-6DC9-5FD3-B541-8DA054FFB32D

http://zoobank.org/3D02F68F-741F-4045-A1D3-0B1A7F52A91B

##### Type species.

*Siamlema changhai* sp. nov. from Trang Province, Thailand.

##### Etymology.

The generic name is derived from “Siam”, referring to the old name of Thailand, and “-lema” is a convention from *Telema*, the type genus of the family. Feminine in gender.

##### Diagnosis.

**Figure 20.**
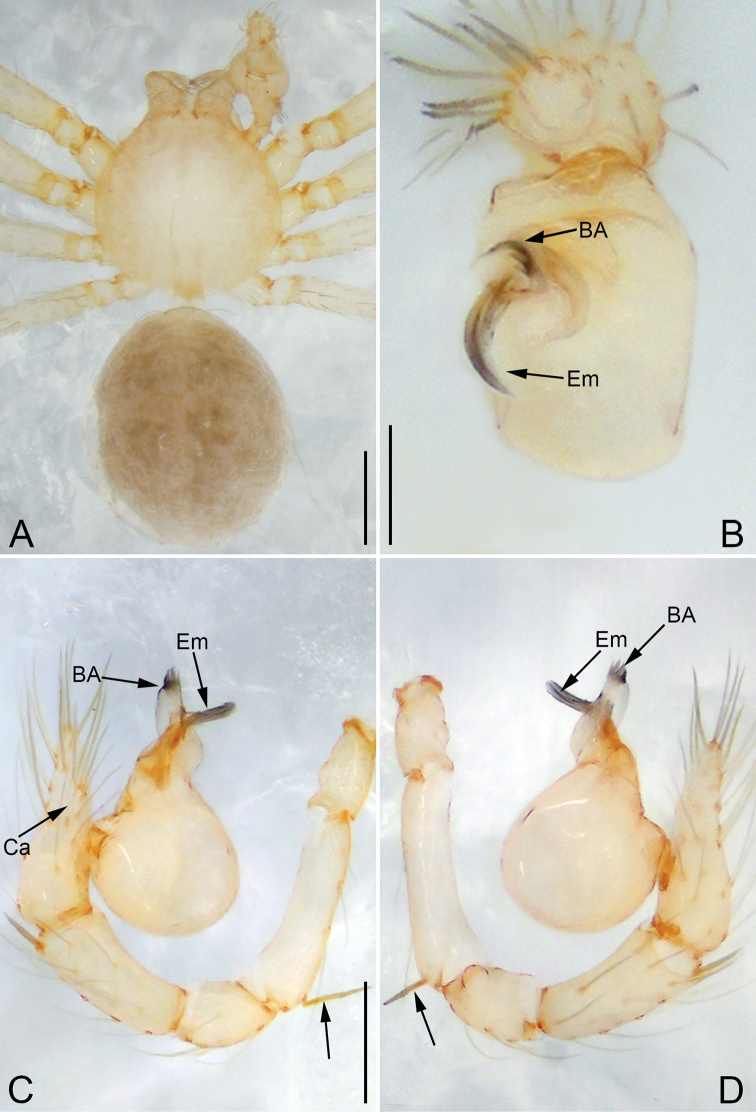
*Siamlema changhai* sp. nov., male holotype. **A** Habitus, dorsal view **B** embolus, apical view **C** palp, prolateral view **D** palp, retrolateral view. Scale bars: 0.2 mm (**A**), 0.05 mm (**B**), 0.1 mm (**C, D**).

**Figure 21.**
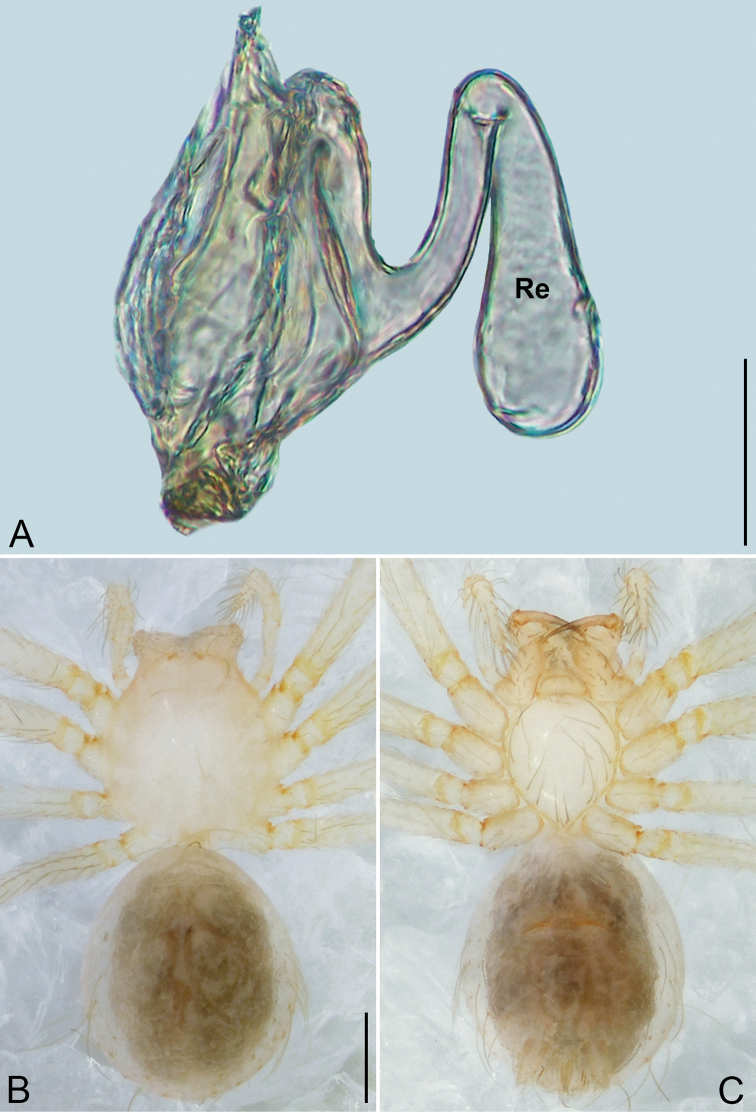
*Siamlema changhai* sp. nov., female paratype. **A** Endogyne, lateral view **B** habitus, dorsal view **C** habitus, ventral view. Scale bars: 0.05 mm (**A**), 0.2 mm (**B, C**).

*Siamlema* gen. nov. can be distinguished from *Telema* by the following: belt-shaped tibial glands (Fig. 1E) (vs. plate-shaped); males can be distinguished from those of *Telema* by having a cymbial apophysis (Fig. 20C) (vs. absent), a distinct dorsal spine present on the palpal tibia (Fig. 20C, D) (vs. absent), and the palpal femur is longer than the cymbium (Fig. 20C, D) (vs. shorter). Receptacle without tubes inside (Fig. 21A) (vs. receptacle with membranous tubes).

##### Description.

Total length: 0.92–1.11 (male), 1.04–1.20 (female). Carapace 0.41–0.48 long. Sternum with sparse setae. Eyes normally developed, vestigial, or absent. Leg formula: 1-2-4-3, tibia I 0.97–1.05 long, leg glands belt shaped (arrows on Fig. 1D). Male palp: cymbium, tibia, patella, and femur robust relative to bulb, femur longer than tibia and cymbium, dorsal spine present on distal part of palpal tibia, distinct cymbial apophysis located prolaterally on mesial part; bulb droplet-shaped or nearly globular; embolus sclerotized. Endogyne: receptacle tube-like or globular, without tubes inside (Fig. 21A).

##### Distribution.

Thailand (Trang, Yala, sites 7, 8 in Fig. 33).

##### Composition.

*Siamlema changhai* sp. nov. and *S. suea* sp. nov. (Fig. 34).

#### Siamlema changhai

Taxon classificationAnimaliaAraneaeTelemidae

Zhao & Li sp. nov.

BE41BB41-ED55-5584-8280-D3DB9A0BAB71

http://zoobank.org/38E14102-FE4E-4C61-B436-2936DC5A6D3B

Figures 1D , 20 , 21 , 33

##### Type material.

Holotype: ♂ (IZCAS), Thailand, Trang Province, Noyong District, Chang Hai Cave, 7.5893N, 99.6688E, elevation ca. 32 m, XI.2015, Z. Chen, G. Zhou, and Q. Zhao leg. Paratypes: 2♂ and 4♀ (IZCAS), same data as holotype.

##### Other material examined.

1♀ (molecular voucher, IZCAS), same data as holotype.

##### Etymology.

The species name refers to the type locality; noun in apposition.

##### Diagnosis.

This species resembles *S. suea* sp. nov. but can be distinguished by the following: the absence of eyes (Figs 20A, 21B) (vs. presence), the presence of a bulbal apophysis (Fig. 20B–D) (vs. absence), the presence of a dorsal spine on the palpal femur distally (arrows on Fig. 20C, D) (vs. spine absent), and the absence of a spine on the palpal tibia retrolaterally (Fig. 20D) (vs. spine present). Receptacle is tube-shaped (Fig. 21A) (vs. globular).

##### Description.

**Male (holotype).** Total length 0.92. Carapace 0.41 long, 0.40 wide. Abdomen 0.50 long, 0.44 wide. Carapace brown (Fig. 20A). Eyes absent (Fig. 20A). Chelicerae, legs, labium, and endites brown. Sternum bright brown. Leg measurements: I 3.28 (0.95, 0.17, 1.00, 0.69, 0.47); II 2.93 (0.86, 0.16, 0.85, 0.63, 0.43); III 2.17 (0.66, 0.14, 0.58, 0.43, 0.36); IV 2.73 (0.86, 0.14, 0.75, 0.56, 0.42). Abdomen brown with a few long setae (Fig. 20A).

Palp. Tibia 1.57 times longer than patella, cymbium 1.22 times longer than tibia, femur 1.06 times longer than cymbium (Fig. 20C, D); cymbial apophysis present at sub-distal part (Fig. 20C), one femoral distal spine (Fig. 20C, D), a semi-round extension on patella proximally (Fig. 20D); bulb shaped as in Fig. 20C, D; bulbal apophysis nearly transparent, with 4 sclerotized teeth (Fig. 20B–D); embolus sclerotized, finger shaped (Fig. 20B–D).

**Female.** Total length 1.04. Carapace 0.48 long, 0.41 wide. Abdomen 0.56 long, 0.50 wide. Carapace, sternum, legs milky white (Fig. 21B, C). Leg measurements: I 3.31 (1.00, 0.17, 0.99, 0.67, 0.48); II 2.97 (0.90, 0.16, 0.86, 0.60, 0.45); III 2.18 (0.67, 0.14, 0.59, 0.42, 0.36); IV 2.73 (0.86, 0.15, 0.75, 0.56, 0.41). Abdomen dark brown with some long setae (Fig. 21B, C). Receptacle tube-shaped, neck two times thinner than distal part (Fig. 21A).

##### Distribution.

Thailand (Trang, site 9 in Fig. 33), known only from the type locality.

#### Siamlema suea

Taxon classificationAnimaliaAraneaeTelemidae

Zhao & Li sp. nov.

71608FBD-3C4D-5377-898C-9BFBDE6D6D09

http://zoobank.org/663258A4-7C24-44AA-9422-7EC1BE6F0430

**Figure 22.**
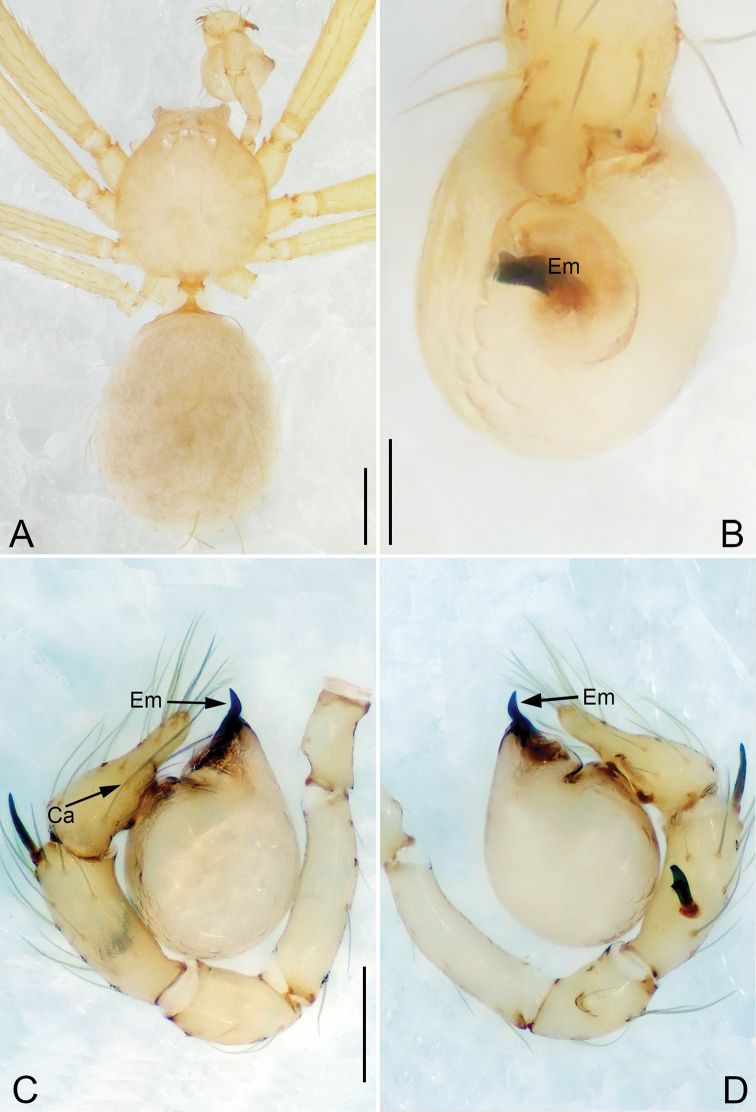
*Siamlema suea* sp. nov., male holotype. **A** Habitus, dorsal view **B** embolus, apical view **C** palp, prolateral view **D** palp, retrolateral view. Scale bars: 0.2 mm (**A**), 0.05 mm (**B**), 0.1 mm (**C, D**).

**Figure 23.**
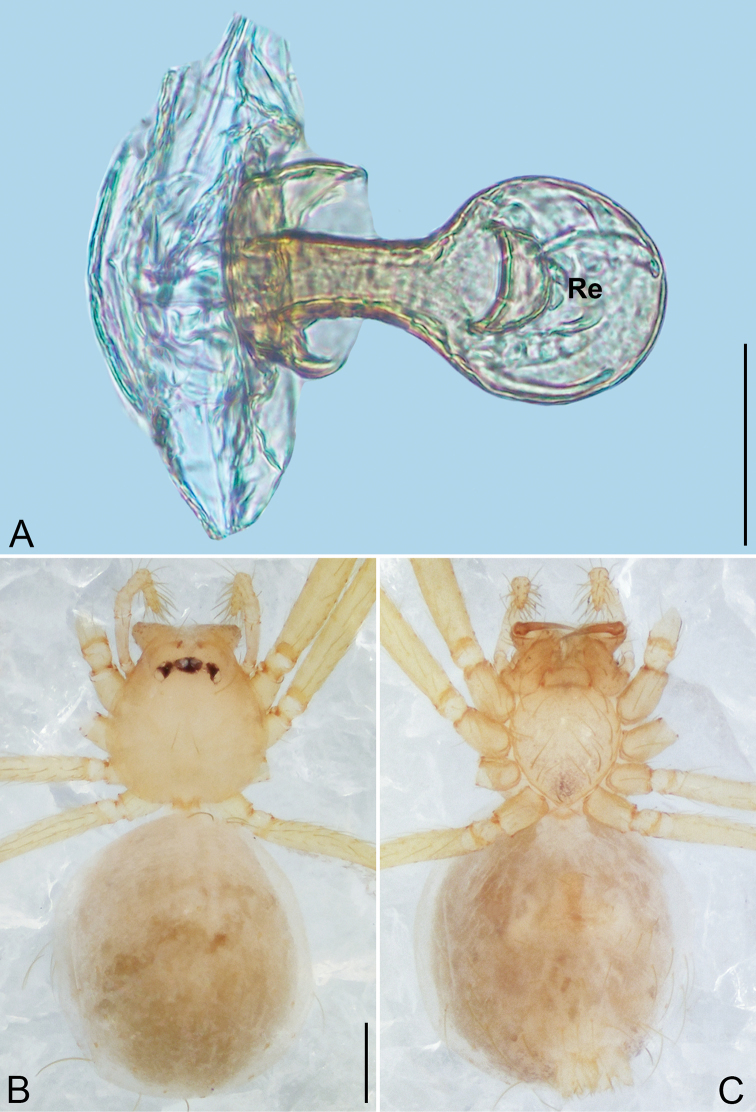
*Siamlema suea* sp. nov., female paratype. **A** Endogyne, lateral view **B** habitus, dorsal view **C** habitus, ventral view. Scale bars: 0.05 mm (**A**), 0.2 mm (**B, C**).

Figures 22 , 23 , 33

##### Type material.

Holotype: ♂ (IZCAS), Thailand, Yala Province, Mueang District, Suea Cave, 6.5226N, 101.2311E, elevation ca. 43 m, XI.2015, Z. Chen, G. Zhou, and Q. Zhao leg. Paratypes: 1♂ and 4♀ (IZCAS), same data as holotype.

##### Other material examined.

1♀ (molecular voucher, IZCAS), same data as holotype.

##### Etymology.

The species name refers to the type locality; noun in apposition.

##### Diagnosis.

This species resembles *S. changhai* sp. nov. but can be distinguished by the following: six eyes ringed with black or eyes vestigial (Figs 22A, 23B) (vs. absent); the lack of a bulbal apophysis (Fig. 22B–D) (vs. present), having a spine on the palpal tibia retrolaterally (Fig. 22D) (vs. absent), the absence of a femoral dorsal spine (Fig. 22C, D) (vs. present); and a globular receptacle (Fig. 23A) (vs. tube-shaped).

##### Description.

**Male (holotype).** Total length 1.11. Carapace 0.46 long, 0.41 wide. Abdomen 0.60 long, 0.50 wide. Carapace light brown (Fig. 22A). Six vestigial eyes (Fig. 22A). Chelicerae, legs, labium, and endites brown. Sternum bright brown with sparse setae. Leg measurements: I 3.46 (1.03, 0.16, 1.05, 0.74, 0.48); II 2.92 (0.90, 0.16, 0.83, 0.60, 0.43); III 2.07 (0.63, 0.13, 0.59, 0.40, 0.32); IV 2.77 (0.85, 0.16, 0.77, 0.59, 0.40). Abdomen brown with a few long setae (Fig. 22A).

Palp. Tibia 1.38 times longer than patella, cymbium 0.94 times shorter than tibia, femur 1.17 times longer than cymbium (Fig. 22C, D); cymbial apophysis stout and cone shaped (Fig. 22C); tibia with 2 spines, one dorsal spine distally, the other bifurcate, located retrolaterally medially (Fig. 22D); patella with retrolateral semi-round extension mesially (Fig. 22D); bulb droplet shaped as in Fig. 22C, D; embolus strongly sclerotized, and tiny in comparison to bulb.

**Female.** Total length 1.20. Carapace 0.45 long, 0.41 wide. Abdomen 0.70 long, 0.66 wide. Coloration as in male (Fig. 23B, C). Six eyes ringed with black (Fig. 23B). Leg measurements: I 3.15 (0.94, 0.16, 0.97, 0.61, 0.47); II 2.79 (0.84, 0.16, 0.81, 0.55, 0.43); III 2.02 (0.62, 0.13, 0.56, 0.38, 0.33); IV 2.73 (0.85, 0.14, 0.76, 0.56, 0.42). Abdomen brown (Fig. 23B). Neck of receptacle umbrella-shaped, and distal part of receptacle globular (Fig. 23A).

##### Distribution.

Thailand (Yala, site 8 in Fig. 33), known only from the type locality.

#### Genus Sundalema

Taxon classificationAnimaliaAraneaeTelemidae

Zhao & Li gen. nov.

C4A40329-EB51-5E8B-8B92-92FF3946E590

http://zoobank.org/8BC0DB45-C792-4792-87DB-8C1685D06AAB

##### Type species.

*Sundalema bonjol* sp. nov. from West Sumatra Province, Indonesia.

##### Etymology.

The generic name is derived from “Sunda”, referring to Sundaland (distributional range of this genus), and “-lema” is a convention from the type genus of the family. Feminine in gender.

##### Diagnosis.

**Figure 24.**
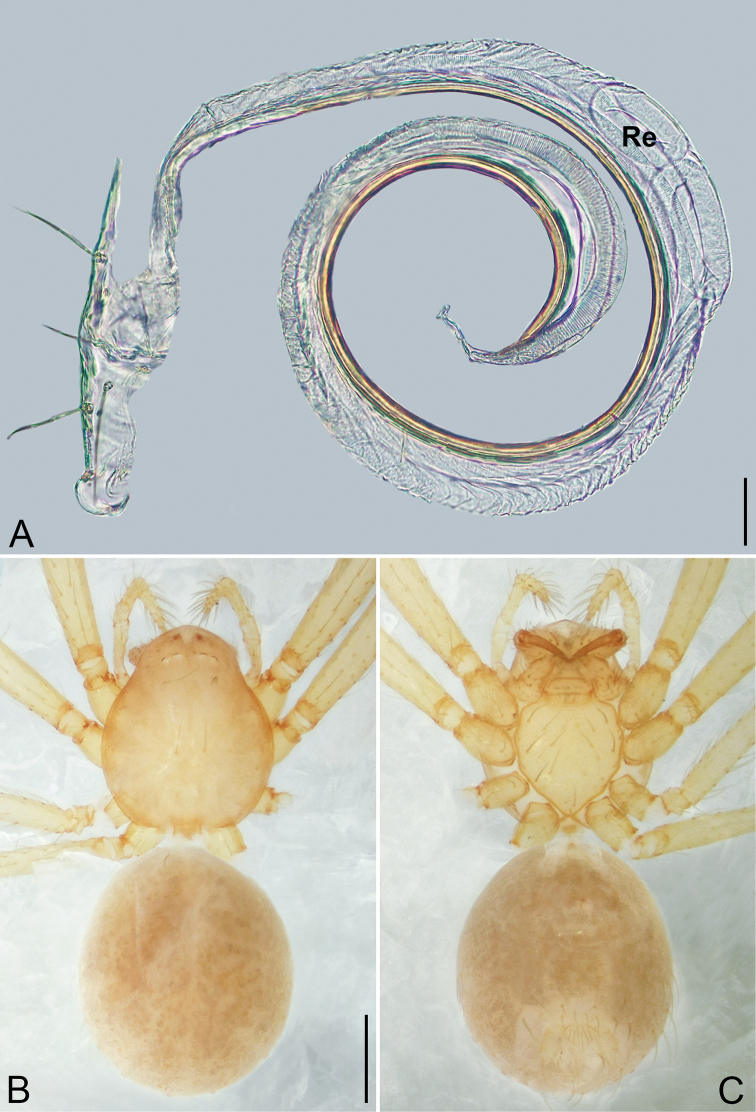
*Sundalema bonjol* sp. nov., female holotype. **A** Endogyne, lateral view **B** habitus, dorsal view **C** habitus, ventral view. Scale bars: 0.05 mm (**A**), 0.2 mm (**B, C**).

**Figure 25.**
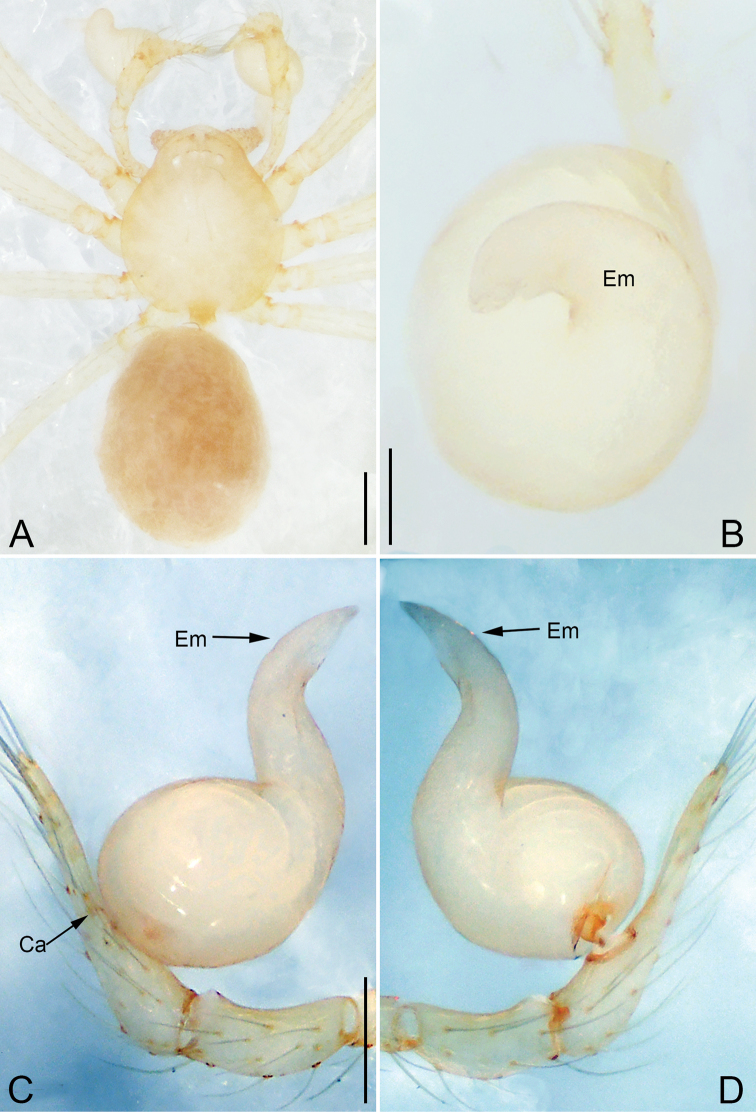
*Sundalema bonjol* sp. nov., male paratype. **A** Habitus, dorsal view **B** embolus, apical view **C** palp, prolateral view **D** palp, retrolateral view. Scale bars: 0.2 mm (**A**), 0.05 mm (**B**), 0.1 mm (**C, D**).

Females belonging to *Sundalema* gen. nov. can be distinguished from those of other genera by the following: a sclerotized and spiral receptacle (Fig. 24A) (vs. unsclerotized or not spiral). Males belonging to *Sundalema* gen. nov. resemble species in the *bailongensis*-group by having a long embolus but can be distinguished by the embolus lacking a spiral ridge and being nearly L-shaped (Fig. 25B–D) (vs. a distinct spiral ridge present on the nearly straight embolus).

##### Description.

Total length: 0.98–1.10 (male), 1.05–1.25 (female). Carapace 0.44– 0.58 long. Sternum light brown or milky white, with several sparse setae. Six vestigial eyes or ringed with black. Leg formula: 1-2-4-3, tibia I 0.90–1.27 long, leg glands belt-shaped (Fig. 1E). For females, neck of receptacle membranous, receptacle spiral, its dorsal part sclerotized, coiled into 1.25 to 2.5 loops. For males, cymbial apophysis tiny, present meso-prolaterally; embolus long relative to bulb, nearly L-shaped, lacking spiral ridge. Abdomen blue or pale brown with several long setae.

##### Composition.

*Sundalema acicularis* comb. nov., *S. anguina* (Wang & Li, 2010) comb. nov., *S. bonjol* sp. nov., and *S. khaorakkiat* sp. nov.

##### Distribution.

Southeast Asia (Thailand and Indonesia, sites 9–12 in Fig. 33).

##### Comments.

The females of this genus are easily distinguished from all other genera, as their receptacles are long, spiral, and sclerotized; thus, a female has been chosen to represent the holotype.

#### Sundalema bonjol

Taxon classificationAnimaliaAraneaeTelemidae

Zhao & Li sp. nov.

D44A7CAF-8252-502C-87AF-242584AE52BA

http://zoobank.org/ECA45DEA-6F29-474A-BE00-C34081595213

Figures 24 , 25 , 33

##### Type material.

Holotype: ♀ (IZCAS), Indonesia, Sumatra, West Sumatra Province, Payakumbuh, Koto Tinggi Village, Imam Bonjol Cave, 0.0637S, 100.3451E, elevation ca. 962 m, III.2014, Z. Yao leg. Paratypes: 2♂ and 3♀ (IZCAS), same data as holotype.

##### Other material examined.

1♀ (molecular voucher, IZCAS), same data as holotype.

##### Etymology.

The species refers to the type locality; noun in apposition.

##### Diagnosis.

The species resembles *S. anguina* comb. nov. but can be distinguished by the following characters: the eyes are vestigial (Figs 24B, 25A) (vs. ringed with black); the receptacle is coiled into 1.5 loops (Fig. 24A) (vs. 2 loops, cf. Wang and Li 2010a: fig. 4C); the embolus is thin and bent at an obtuse angle (Fig. 25B–D) (vs. wide and bent at a right angle, cf. Wang and Li 2010a: fig. 4A, B).

##### Description.

**Female (holotype).** Total length 1.09. Carapace 0.53 long, 0.45 wide. Abdomen 0.64 long, 0.56 wide. Carapace light brown (Fig. 24B). Four vestigial eyes (Fig. 24B). Chelicerae, legs, labium, endites, and sternum bright brown. Sternum with sparse setae (Fig. 24B, C). Leg measurements: I 3.45 (1.04, 0.18, 1.06, 0.67, 0.50); II 3.10 (0.96, 0.17, 0.92, 0.60, 0.45); III 2.17 (0.68, 0.17, 0.58, 0.40, 0.34); IV 2.89 (0.90, 0.16, 0.79, 0.61, 0.43). Abdomen brown (Fig. 24B, C). Receptacle coiled into 1.5 loops, and neck as wide as distal part (Fig. 24A).

**Male.** Total length 1.03. Carapace 0.47 long, 0.40 wide. Abdomen 0.53 long, 0.44 wide. Coloration as in female (Fig. 25A). Leg measurements: I 3.34 (0.94, 0.16, 1.04, 0.71, 0.49); II 3.05 (0.90, 0.16, 0.91, 0.63, 0.45); III 2.16 (0.67, 0.14, 0.58, 0.42, 0.35); IV 2.82 (0.87, 0.15, 0.79, 0.59, 0.42). Palp: tibia 1.80 times longer than patella, cymbium 1.71 times longer than tibia, 1.69 times longer than femur; length of cymbial apophysis as wide as 1/2 width of cymbial base (Fig. 25C); bulb ellipsoidal (Fig. 25C, D); embolus tube-like, as long as major-axis of bulb (Fig. 25B–D).

##### Distribution.

Indonesia (Sumatra, site 11 in Fig. 33), known only from the type locality.

#### Sundalema khaorakkiat

Taxon classificationAnimaliaAraneaeTelemidae

Zhao & Li sp. nov.

3C9A4F73-7888-5118-80E6-F8661A002181

http://zoobank.org/6D05CD58-B0CF-48B4-99B7-D09DD62F0CB0

**Figure 26.**
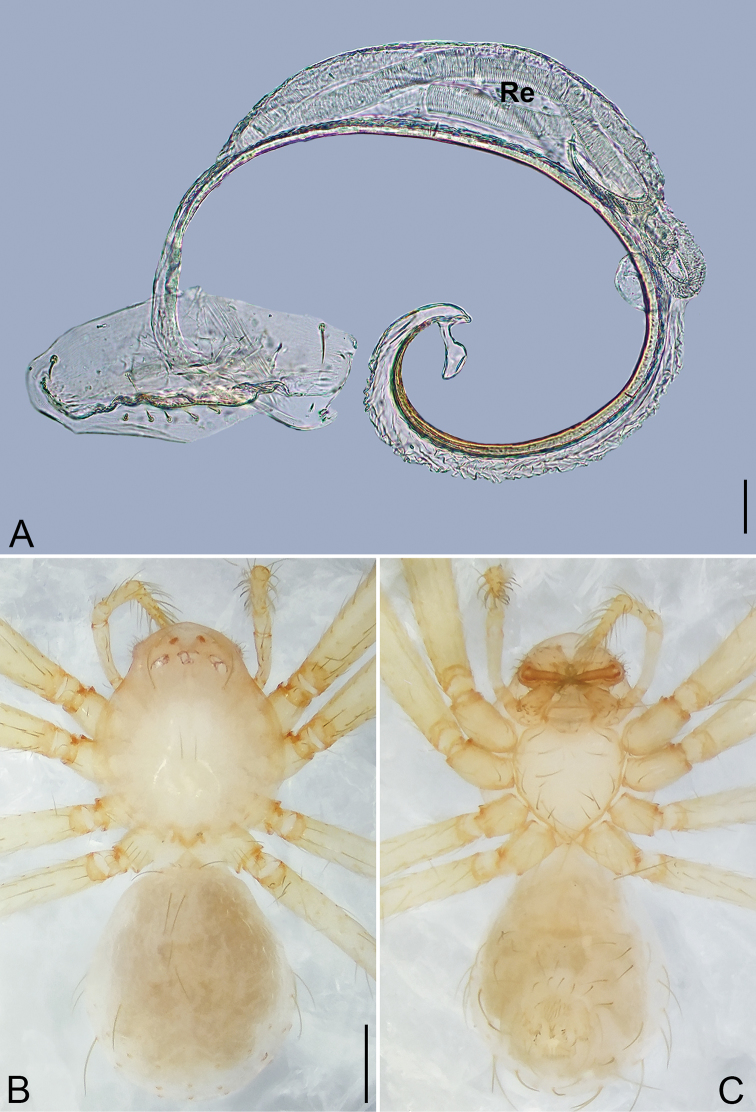
*Sundalema khaorakkiat* sp. nov., female holotype. **A** Endogyne, lateral view **B** habitus, dorsal view **C** habitus, ventral view. Scale bars: 0.05 mm (**A**), 0.2 mm (**B, C**).

**Figure 27.**
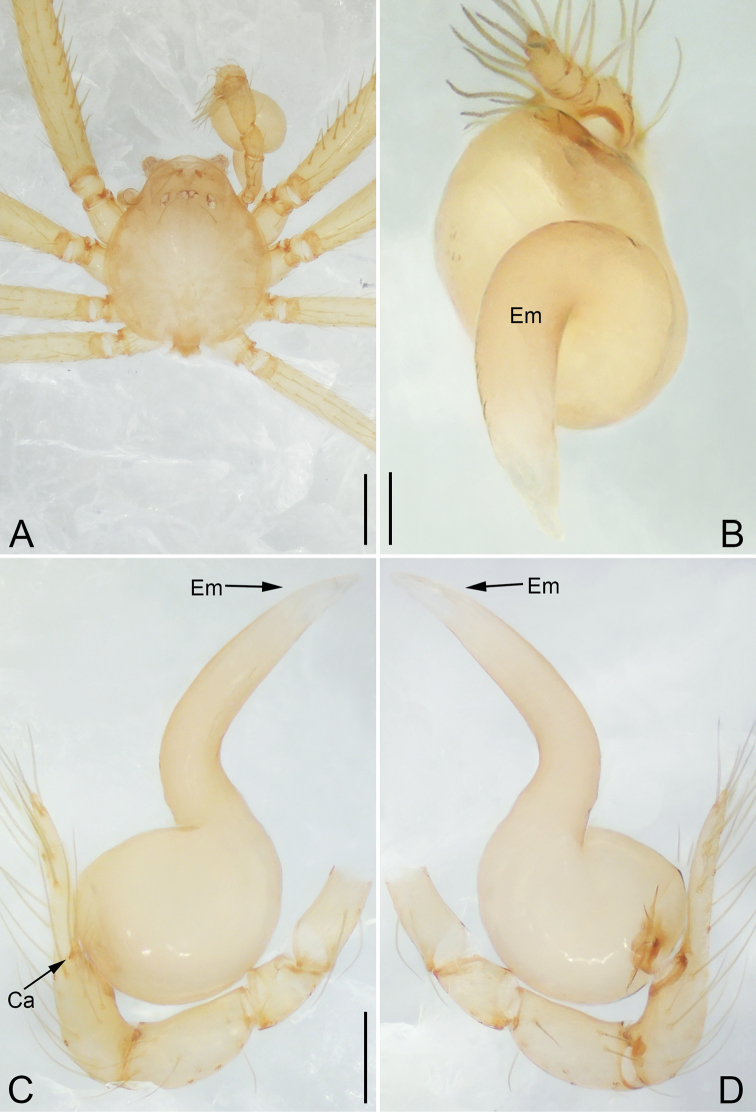
*Sundalema khaorakkiat* sp. nov., male paratype. **A** Habitus, dorsal view **B** embolus, apical view **C** palp, prolateral view **D** palp, retrolateral view. Scale bars: 0.2 mm (**A**), 0.05 mm (**B**), 0.1 mm (**C, D**).

Figures 26 , 27 , 33

##### Type material.

Holotype: ♀ (IZCAS), Thailand, Songkhla Province, Rattaphum District, Khao Rak Kiat Cave, 7.0724N, 100.2502E, elevation ca. 52 m, XI.2015, Z. Chen, G. Zhou and Q. Zhao leg. Paratypes: 2♂ and 2♀ (IZCAS), same data as holotype.

##### Other material examined.

1♀ (molecular voucher, IZCAS), same data as holotype.

##### Etymology.

The species name refers to the type locality; noun in apposition.

##### Diagnosis.

This species resembles *S. acicularis* comb. nov. but can be distinguished by the following characters: the eyes are vestigial (Figs 26B, 27A) (vs. ringed with black); the receptacle is coiled into 1.25 loops (Fig. 26A) (vs. 2.5 loops); the embolus is longer than the cymbium (Fig. 27C, D) (vs. shorter), and the palpal tibia/cymbium ratio is 0.4 (Fig. 27C, D) (vs. 0.45).

##### Description.

**Female (holotype).** Total length 1.25. Carapace 0.58 long, 0.50 wide. Abdomen 0.63 long, 0.55 wide. Carapace pale white (Fig. 26B). Six vestigial eyes (Fig. 26B). Chelicerae, legs, labium milky white, endites brown (Fig. 26B). Sternum milky white with sparse setae (Fig. 26B). Leg measurements: I 3.93 (1.21, 0.19, 1.21, 0.80, 0.52); II 3.52 (1.10, 0.19, 1.06, 0.72, 0.45); III 2.45 (0.73, 0.18, 0.69, 0.49, 0.36); IV 3.28 (1.03, 0.18, 0.92, 0.70, 0.45). Abdomen pale brown with a few long setae (Fig. 26B, C). Receptacle coiled into 1.25 loops, and neck much thinner than mesial part (Fig. 26A).

**Male.** Total length 1.17. Carapace 0.54 long, 0.48 wide. Abdomen 0.63 long, 0.50 wide. Eyes and coloration as in female (Fig. 27A). Leg measurements: I 4.08 (1.24, 0.19, 1.27, 0.86, 0.53); II 3.70 (1.13, 0.19, 1.15, 0.75, 0.48); III 2.67 (0.81, 0.16, 0.76, 0.56, 0.39); IV 3.48 (1.06, 0.17, 1.00, 0.76, 0.49). Palp: Tibia 1.65 times longer than patella, cymbium 2.06 times longer than tibia, 1.50 times longer than femur; cymbial apophysis dark brown and cone shaped (Fig. 27C); bulb ellipsoidal; embolus longer than cymbium (Fig. 27C, D).

##### Distribution.

Thailand (Songkhla, site 12 in Fig. 33), known only from the type locality.

#### Sundalema acicularis

Taxon classificationAnimaliaAraneaeTelemidae

(Wang & Li, 2010) comb. nov.

197FC960-A3F4-5FEB-B79E-EA831CCF9DE1

Telema acicularis Wang and Li 2010a: 2, figs 1–3 (♂♀).

##### Type material.

Holotype: ♂ (RMNH), Thailand, Prachuap Khiri Khan Province, Hua Hin, Tham Kaew Cave, 12.5947N, 99.9571E, 3.I.1989, C.L. Deeleman-Reinhold leg. Paratypes: 1♂ and 1♀ (RMNH), same data as holotype. Examined.

##### Diagnosis.

This species resembles *S. khaorakkiat* sp. nov. but can be distinguished by the following characters: six eyes ringed with black (vs. eyes vestigial); receptacle coiled into 2.5 loops (cf. Wang and Li 2010a: fig. 1B) (vs. 1.25 loops); embolus shorter than cymbium (cf. Wang and Li 2010a: fig. 1A) (vs. longer), and the palpal tibia/cymbium length ratio is 0.45 (cf. Wang and Li 2010a: fig. 1A) (vs. 0.4).

##### Description-amendments.

Palpal tibia/cymbium ratio 0.45 in the male. Receptacle coiled into 2.5 loops (cf. Wang and Li 2010a: fig. 1B). For a more detailed description, see Wang and Li (2010a).

##### Distribution.

Thailand (Prachuap Khiri Khan, site 9 in Fig. 33), known only from the type locality.

#### Sundalema anguina

Taxon classificationAnimaliaAraneaeTelemidae

(Wang & Li, 2010) comb. nov.

D5708785-FDE0-5477-853B-5B41719A1DBE

Figure 1E

Telema anguina Wang and Li 2010a: 2, figs 4–7 (♂♀).

##### Type material.

Holotype: ♂ (RMNH), Thailand, Krabi Province, Diamond Cave, 8.0670N, 98.9164E. 3.I.1989, C.L. Deeleman-Reinhold leg. Paratypes 1♂ and 1♀ (RMNH), same data as holotype. Examined.

##### Other material examined.

3♀ (including one molecular voucher, IZCAS) from the type locality, X.2014, Z. Chen and Y. Li leg.

##### Diagnosis.

This species resembles *S. bonjol* sp. nov. but can be distinguished by the following characters: the eyes are ringed with black (cf. Wang and Li 2010a: fig. 6B) (vs. vestigial); the receptacle is coiled into two loops (cf. Wang and Li 2010a: figs 4C, 7B) (vs. 1.5 loops); the embolus is thin and bent at a right angle (cf. Wang and Li 2010a: fig. 4A, B) (vs. much wider and obtuse angled).

##### Description.

See Wang and Li (2010a).

##### Distribution.

Thailand (Krabi, site 10 in Fig. 33), known only from the type locality.

#### Genus Telema

Taxon classificationAnimaliaAraneaeTelemidae

Simon, 1882

ED63BB54-ABA7-5576-A980-C795014E1EC6

Telema Simon 1882: 205; Lehtinen 1967: 301; Gertsch 1973: 154; Yaginuma 1973: 17; Wang et al. 2012: 18; Dupérré and Tapia 2015: 191; Zhao et al. 2018a: 14.

##### Type species.

*Telema tenella* Simon, 1882 from southern France.

##### Diagnosis.

*Telema* resembles *Telemofila* but can be distinguished by the following: plate-shaped tibial glands (Fig. 1A; cf. Emerit 1984: fig. A) (vs. belt-shaped); lacking a cymbial apophysis (cf. Wang et al. 2012: fig. 2C) (vs. cymbial apophysis present); the endogyne is walking-stick shaped (cf. Wang et al. 2012: fig. 3C, D) (vs. bean-shaped or globular).

##### Description.

Total length: 1.24–1.48 (male), 1.06–1.90 (female). Carapace 0.45–0.78 long. In *T. auricoma* Lin & Li, 2010 and *T. nipponica*, carapace with radial shaded pattern, six eyes ringed with black, sternum dark brown, abdomen blue; in *T. tenella*, *T. guihua* Lin & Li, 2010, and *T. wunderlichi* Song & Zhu, 1992, carapace without pattern, eyes absent, sternum and abdomen bright brown. Leg formula: 1-2-4-3, tibia I 1.32–2.45, tibia with many plate-shaped glands (Fig. 1A; cf. Emerit 1984: fig. A). In male, length of cymbium > femur > tibia > patella; bulb ellipsoid, large relative to body; embolus short relative to bulb, duckbill shaped. Receptacle walking-stick shaped, with a few membranous tubes inside.

##### Composition.

*Telema auricoma*, *T. guihua*, *T. nipponica*, *T. tenella* and *T. wunderlichi*. The composition is supported not only by morphological characters but also by molecular phylogenetic analyses (Fig. 34; *T. nipponica* is not included).

##### Distribution.

Eurasian disjunctive range, known from Southern Europe (France and Spain, one species) and East Asia (Japan and southwestern China, four species, sites 13–16 in Fig. 33).

#### Telema auricoma

Taxon classificationAnimaliaAraneaeTelemidae

Lin & Li, 2010

D116C0D1-4A42-5C49-8F0A-D27F8B6E6D7F

Telema auricoma Lin and Li 2010: 3, fig. 1 (♀).

##### Type material.

Holotype: ♀ (IZCAS), China, Guizhou Province, Dafang County, Xiaotun Town, Shiqiang Village, Daxiao Cave, 27.0925N,105.5551E, 1168m, 4.V.2007, J. Liu and Y. Lin leg. Paratypes 2♀ (IZCAS), same data as holotype. Examined.

##### Other material examined.

1♀ (molecular voucher, IZCAS), same data as holotype; 2♀ (IZCAS), Guizhou Province, Bijie County, Xiaoyan Cave, 27.1181N, 105.2367E; 1♀ (IZCAS) Guizhou Province, Dafang County, Shilongshang Cave, 27.0925N, 105.5551E; 3♀ (IZCAS) Guizhou Province, Hezhang County, Tanjiayan Cave, 27.2003N, 104.5910E; 1♀ (IZCAS) Guizhou Province, Weining County, Banbianshan Cave, 33.9061N, 104.5396E; 2♀ (IZCAS), Yunnan Province, Xuanwei County, Jianjiao Cave, 33.3085N, 104.3900E; 3♀ (IZCAS), Yunnan Province, Xuanwei County, Fengchao Cave, 33.3909N, 104.2093E.

##### Diagnosis and description.

See Lin and Li (2010).

##### Distribution.

China (Yunnan-Guizhou Plateau).

##### Comments.

All known specimens of this species are female, and molecular barcoding data from all eight populations examined shows no differences (unpublished data). This indicates that the species may be parthenogenetic, a character that may allow it to easily disperse more broadly than gametogenetic species.

#### Telema guihua

Taxon classificationAnimaliaAraneaeTelemidae

Lin & Li, 2010

CFF55A0E-ED6C-5DEC-AD26-977439D6FB06

Figures 1A , 33

Telema guihua Lin and Li 2010: 12, figs 6, 7 (♂♀).

##### Type material.

Holotype: ♂ (IZCAS), China, Guizhou Province, Suiyang County, Wenquan Town, Guihua Village, Mahuang Cave, 28.2437N,107.2891E, elevation ca. 730 m, 4.V.2007, J. Liu and Y. Lin leg. Paratypes: 1♂ and 2♀ (IZCAS), same data as holotype. Examined.

##### Other material examined.

1♀ (molecular voucher, IZCAS), same data as holotype.

##### Diagnosis.

This species resembles *T. wunderlichi* but can be distinguished by the following characters: the small body size (vs. larger size); the embolus is membranous (vs. sclerotized), and the tip of the embolus is blunt (cf. Lin and Li 2010: fig. 6E) (vs. sharply pointed).

##### Description.

See Lin and Li (2010).

##### Distribution.

China (Guizhou, site 14 in Fig. 33), known only from the type locality.

#### Telema nipponica

Taxon classificationAnimaliaAraneaeTelemidae

(Yaginuma, 1972)

CF6A927C-D381-5CAC-9569-2CADAB6F63B3

Merizocera nipponica Yaginuma 1972: 286, figs 10–14 (♀).
Telema nipponica : Yaginuma 1973: 22, figs 1–6 (♂♀); Yaginuma 1974: 14, figs 4–6 (♂); Shinkai 1977: 322, figs 1–3 (♂); Yaginuma 1986: 20, fig. 13.3 (♂); Chikuni 1989: 26, fig. 1 (♂); Ono 2009: 121, figs 1–6 (♂♀).

##### Type material.

Holotype: ♀, Japan, Yamanashi Prefecture, Narusawa-mura, Karumizu, Shoiko-daini-fuketsu Cave, 22.IX.1969, S. Ueno and K. Kato leg. Not examined.

##### Diagnosis.

This species resembles *T. tenella* but can be distinguished by the thumb-like shape of the embolus (cf. Yaginuma 1974: fig. 4), whereas in *T. tenella*, the embolus is short and triangular (cf. Wang et al. 2012: fig. 2C, D).

##### Description.

See Yaginuma (1972, 1973).

##### Distribution.

Japan (Site 15 in Fig. 33).

#### Telema wunderlichi

Taxon classificationAnimaliaAraneaeTelemidae

Song & Zhu, 1994

012F4FDC-5174-57D6-8546-7FF61B84D8CD

Telema wunderlichi Song and Zhu 1994: 36, fig. 1A–E (♂♀); Song et al. 1999: 51, fig. 11G, 22A–C (♂♀); Yin et al. 2012: 161, fig. 29a, d (♂♀).

##### Type material.

Holotype: ♀ (IZCAS), China, Hunan Province, Zhangjiajie Prefecture, Sangzhi County, Wulingyuan Scientific and Historic Interest Area, 29.1171N, 110.4792E, elevation ca. 280 m, 27.XI.1992. D. Wang leg. Paratypes: 1♂ and 1♀ (IZCAS), same data as holotype. Examined.

##### Other material examined.

1♂ and 2♀ (including molecular voucher, IZCAS) from the type locality: XII.2015, Z. Chen leg.

##### Diagnosis.

This species resembles *T. guihua* but can be distinguished by the following characters: the larger body size (vs. smaller), the slightly sclerotized embolus (cf. Song and Zhu 1994: fig. 1E) (vs. membranous), the sharply pointed embolus tip (cf. Song and Zhu 1994: fig. 1E) (vs. blunt).

##### Description.

See Song and Zhu (1994), and Lin and Li (2010).

##### Distribution.

China (Hunan, site 16 in Fig. 33).

#### Genus Telemofila

Taxon classificationAnimaliaAraneaeTelemidae

Wunderlich, 1995

03441732-F397-569B-B54E-42DD9AB82563

Telemofila Wunderlich 1995: 562.

##### Type species.

*Telemofila samosirensis* Wunderlich, 1995 from Sumatra, Indonesia.

##### Diagnosis.

*Telemofila* can be distinguished from *Telema* by the following characters: leg formula is 1-4-2-3 (vs. 1-2-4-3), tibial glands are belt-shaped (Fig. 1G) (vs. plate-shaped); a cymbial apophysis is present (vs. absent), the embolus is sickle shaped or claw-like (vs. duckbill-shaped), and the length of the embolus is three times shorter than the diameter of the bulb (vs. two times shorter); the distal part of the receptacle is swollen (vs. not swollen).

##### Description.

Total length: 0.90–1.27 (male), 0.98–1.11 (female). Carapace 0.37–0.50 long. Sternum 0.20–0.25 long, with several sparse setae. Tibia I 0.51–0.82 long, leg formula: 1-4-2-3, belt-shaped glands present (Fig. 1G). Six eyes ringed with black, body blue or brown. For males, cymbial apophysis present mesially, length as wide as cymbial base (cf. Wunderlich 1995: fig. 16), embolus sickle-shaped, length of embolus 1/3 as long as diameter of bulb (cf. Wunderlich 1995: fig. 17). For females, receptacle bean shaped or globular, neck narrower than distal part (cf. Wang and Li 2010a: figs 11A, B, 15A, B).

##### Composition.

*Telemofila fabata* (Wang & Li, 2010) comb. nov., *T. malaysiaensis* comb. nov., *T. pecki* (Brignoli, 1980), and *T. samosirensis* Wunderlich, 1995.

##### Distribution.

Rainforests in Southeast Asia (Indonesia, Singapore, Malaysian Borneo, sites 17–19 in Fig. 33) and a cave in New Caledonia.

##### Comments.

The placement of *T. pecki* in this genus is dubious because its leg formula is 1-2-4-3 (Brignoli 1980) and the embolus is triangular (cf. Brignoli 1980: figs 1, 2). These characters are inconsistent with the genus characters of *Telemofila*. However, we have been unable to examine the types of *T. pecki*, and molecular data from this species is lacking.

#### Telemofila fabata

Taxon classificationAnimaliaAraneaeTelemidae

(Wang & Li, 2010) comb. nov.

30F14952-0B4E-5621-8519-7485657AF747

Telema fabata Wang and Li 2010a: 10, figs 8–11 (♂♀).

##### Type material.

Holotype: ♂ (RMNH), Singapore, Bukit Timah Nature Reserve, Seraya Loop, 1.3521N, 103.8198E, 2.II.1983, P.R. Deeleman leg. Paratypes: 1♂ and 1♀ (RMNH), same data as holotype. Examined.

##### Other material examined.

1♂ and 3♀ (including molecular voucher, IZCAS) from the type locality, VIII.2015, S. Li and Y. Tong leg.

##### Diagnosis.

This species resembles *T. malaysiaensis* comb. nov. but can be distinguished by the following: bigger body size, bean-shaped receptacle (cf. Wang and Li 2010a: fig. 8C, D) (vs. globular); claw-like embolus (cf. Wang and Li 2010a: fig. 8A, B) (vs. sickle-shaped). This species can be distinguished from *T. samosirensis* by the embolus being three times shorter than the bulb (cf. Wang and Li 2010a: fig. 9A, B) (vs. two times shorter).

##### Description.

See Wang and Li (2010a).

##### Distribution.

Singapore (Site 17 in Fig. 33).

#### Telemofila malaysiaensis

Taxon classificationAnimaliaAraneaeTelemidae

(Wang & Li, 2010) comb. nov.

A0B9DBEB-071A-5B18-A2EE-8BBEF56CE7E1

Telema malaysiaensis Wang and Li 2010a: 10, figs 12–15 (♂♀).

##### Type material.

Holotype: ♂ (RMNH), Malaysian Borneo, Sarawak Province, swampy lowland rainforest of Bako National Park, 1.7167N, 110.4667E, 29.III.1985, P.R. & C.L. Deeleman-Reinhold leg. Paratypes: 1♂ and 1♀ (RMNH), same data as holotype. Examined.

##### Diagnosis.

This species resembles *T. fabata* comb. nov. but can be distinguished by the following: the smaller body size, the shape of the receptacle is globular (cf. Wang and Li 2010a: fig. 15B) (vs. receptacle bean-shaped); the embolus is sickle-shaped (cf. Wang and Li 2010a: fig. 12A–C) (vs. claw-like embolus).

##### Description.

See Wang and Li (2010a).

##### Distribution.

Malyasia (Borneo, Sarawak, site 18 in Fig. 33).

#### Telemofila samosirensis

Taxon classificationAnimaliaAraneaeTelemidae

Wunderlich, 1995

D9854967-54F6-5073-902A-B27F7685B47D

Figures 1G , 33

Telemofila samosirensis Wunderlich 1995: 562, figs 10–17 (♂).

##### Type material.

Holotype: ♂ (SMF), Indonesia, Sumatra, North Sumatra Province, Lake Toba, Samosir Village, 2.7424N, 98.7699E, elevation ca. 916 m, VIII. 1994, J. Wunderlich leg. Not examined.

##### Other material examined.

2♀ (including molecular voucher, IZCAS) from the type locality: I.2014, H. Zhao leg.

##### Diagnosis.

This species can be distinguished from *T. fabata* comb. nov. and *T. malaysiaensis* comb. nov. by the length of the embolus which is equal to the radius of the bulb (cf. Wunderlich 1995: fig. 17) (vs. length of embolus 1/3 as long as the diameter of the bulb in two similar species).

##### Descriptions.

See Wunderlich (1995).

##### Distribution.

Indonesia (Sumatra, North Sumatra, site 19 in Fig. 33).

#### Genus Zhuanlema

Taxon classificationAnimaliaAraneaeTelemidae

Zhao & Li gen. nov.

F07B306D-AE49-5280-AD76-051356FED0EF

http://zoobank.org/D5FC4E70-33EA-4741-88FF-85005FF3C759

##### Type species.

*Zhuanlema peteri* sp. nov. from Luang Prabang Province, Laos.

##### Etymology.

The generic name is derived from “Zhuan”, referring to the Chinese pinyin “zhuan”, indicating that the apex of the embolus is twisted, and “-lema” which is a convention from the type genus of the family. Feminine in gender.

##### Diagnosis.

**Figure 28.**
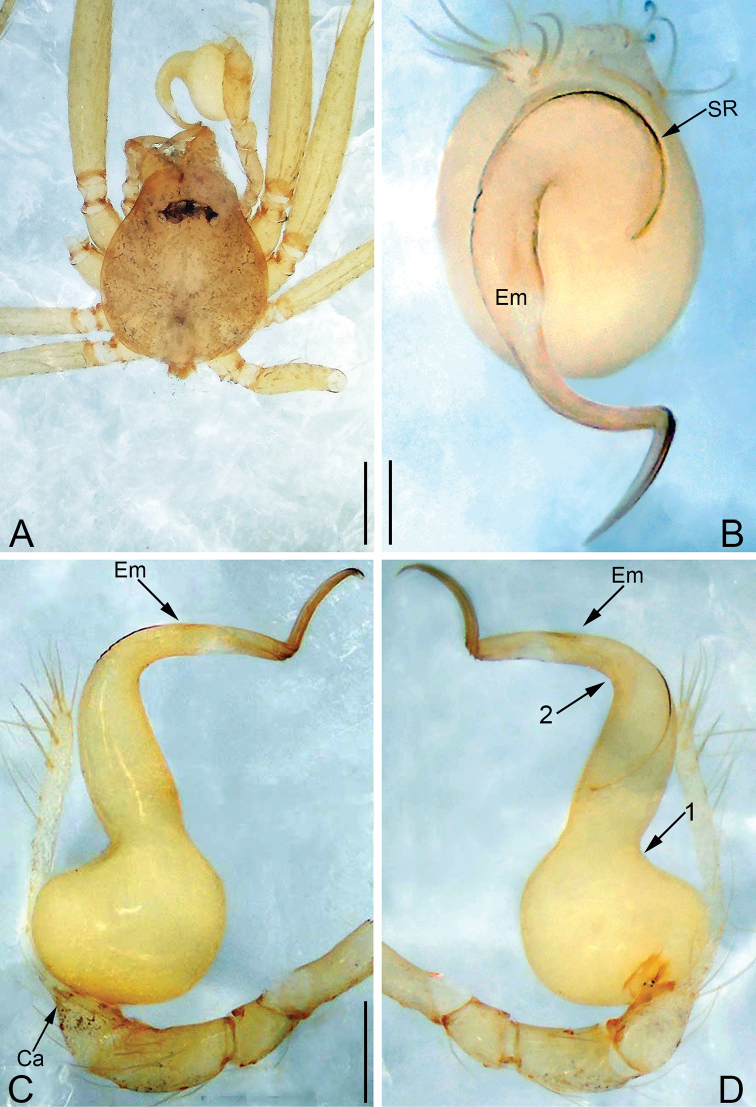
*Zhuanlema peteri* sp. nov., male holotype. **A** Habitus, dorsal view **B** embolus, apical view **C** palp, prolateral view **D** palp, retrolateral view. Scale bars: 0.2 mm (**A**), 0.05 mm (**B**), 0.1 mm (**C, D**).

**Figure 29.**
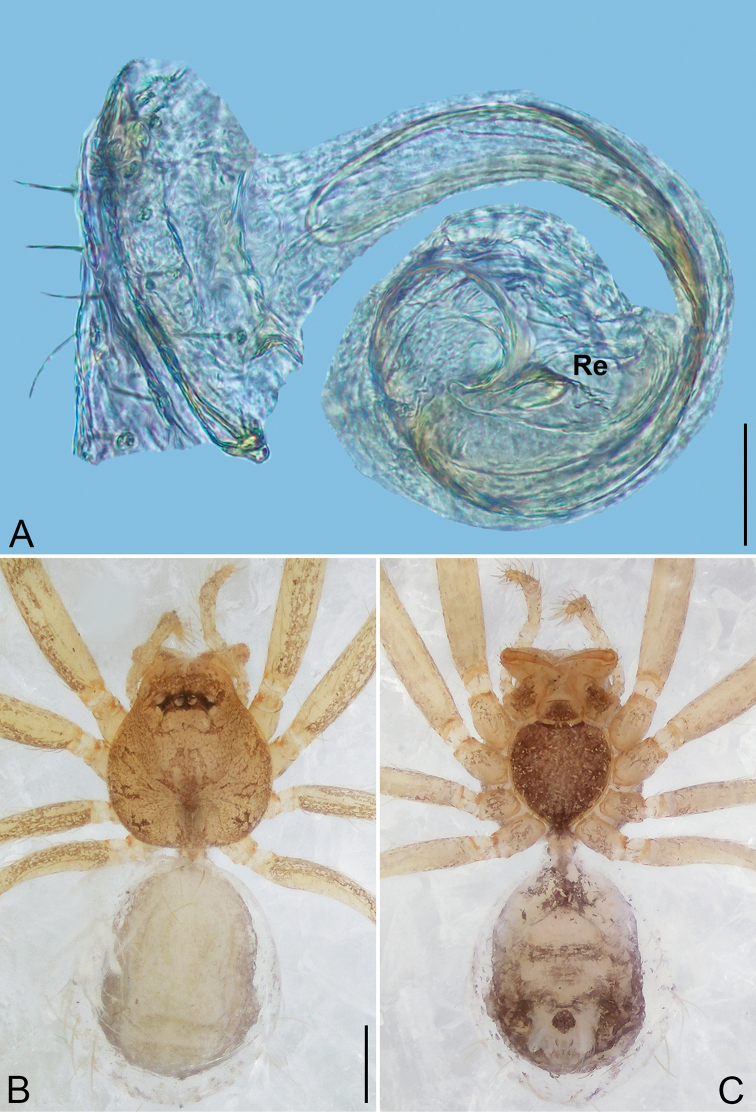
*Zhuanlema peteri* sp. nov., female paratype. **A** Endogyne, lateral view **B** habitus, dorsal view **C** habitus, ventral view. Scale bars: 0.05 mm (**A**), 0.2 mm (**B, C**).

The new genus resembles species in the *bailongensis*-group but can be distinguished by the following characters: the apex of the embolus is twisted (Fig. 28C) (vs. tube-like embolus), the cymbial apophysis is located basally and four times shorter than the width of the cymbial base (Fig. 28C, D) (vs. cymbial apophysis located mesially or sub-basally, and longer or 1–3 times shorter than the width of the cymbial base). Females can be distinguished by the sclerotized receptacle (Fig. 29A) (vs. membranous).

##### Description.

See species description.

##### Composition.

*Zhuanlema peteri* sp. nov.

##### Distribution.

Laos (Luang Prabang, site 20 in Fig. 33), known only from the type locality.

#### Zhuanlema peteri

Taxon classificationAnimaliaAraneaeTelemidae

Zhao & Li sp. nov.

55BCB96C-8AA3-535F-A512-B6EC9D9F0C73

http://zoobank.org/C8F4AC29-E5E1-4486-87BE-756E7954150D

Figures 28 , 29 , 33

##### Type material.

Holotype: ♂ (SMF): Laos, Luang Prabang Province, NE Luang Prabang, Nam Ou, Nong Khiao, Tham Pathok, 20.5514N, 102.6321E, elevation ca. 373 m, 13.III.2007, P. Jäger leg. Paratypes: 1♂ and 2♀ (SMF), same data as holotype.

##### Other material examined.

1♀ (molecular voucher, IZCAS), same data as holotype.

##### Etymology.

The species is named in honor of Peter Jäger (Frankfurt am Main, Germany), a prolific spider taxonomist.

##### Diagnosis.

See genus diagnosis.

##### Description.

**Male (holotype)**: Total length unknown. Carapace 0.49 long, 0.41 wide. Abdomen lost. Carapace brown (Fig. 28A). Six eyes ringed with black (Fig. 28A). Chelicerae, legs, labium, and endites brown. Sternum light brown with sparse setae. Glands belt-shaped, leg measurements: I 2.44 (0.71, 0.14, 0.73, 0.46, 0.40); II 2.23 (0.67, 0.14, 0.61, 0.43, 0.38); III 1.65 (0.49, 0.14, 0.43, 0.31, 0.28); IV 2.20 (0.65, 0.15, 0.61, 0.44, 0.35).

Palp: tibia 2.15 times longer than patella, cymbium 2.80 times longer than tibia, two times longer than femur. Cymbium bent, cymbial apophysis tiny, about 1/4 of cymbial base width (Fig. 28C); bulb shaped as in Fig. 28C, D with very long and twisted embolus, bent at right-angle dorsally on bulb (arrow 1 in Fig. 28D). Embolus with a right-angled bend (arrow 2 in Fig. 28D), its tip twisted and sclerotized slightly (Fig. 28C, D), spiral ridge originates from base of embolus (Fig. 28B).

**Female**: Total length 1.18. Carapace 0.49 long, 0.43 wide. Abdomen 0.63 long, 0.56 wide. Coloration as in male (Fig. 29B, C). Leg measurements: I 2.57 (0.77, 0.15, 0.77, 0.48, 0.40); II 2.34 (0.71, 0.14, 0.67, 0.44, 0.38); III 1.72 (0.54, 0.12, 0.45, 0.33, 0.28); IV 2.32 (0.71, 0.13, 0.66, 0.47, 0.35). Abdomen dark brown. Endogyne as in Fig. 29A, receptacle with sclerotized tube inside, comma-shaped, swollen distally, distal part of receptacle two times wider than neck.

##### Distribution.

Laos (Luang Prabang, site 20 in Fig. 33), known only from the type locality.

**Figure 30.**
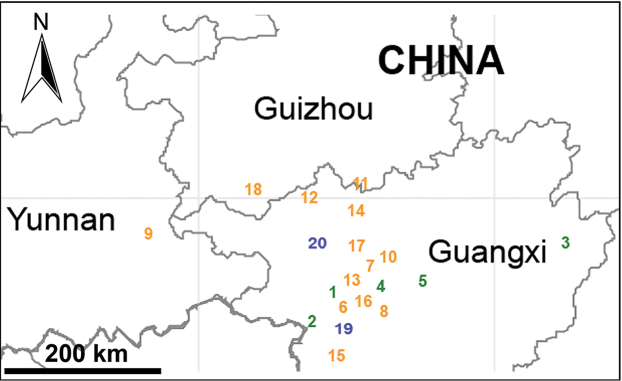
Distribution records of *Pinelema* spp., the *adunca*-group (green), the *bailongensis*-group (orange), and the *cunfengensis*-group (blue): **1***P. adunca* comb. nov. **2***P. qingfengensis***3***P. renalis* comb. nov. **4***P. tortutheca* comb. nov. **5***P. yashanensis* comb. nov. **6***P. bailongensis***7***P. cheni***8***P. cordata***9***P. curcici***10***P. huoyan***11***P. liangxi***12***P. lizhuang***13***P. strentarsi***14***P. wangshang***15***P. wenyang***16***P. xiushuiensis***17***P. yunchuni***18***P. zhewang***19***P. cunfengensis***20***P. spirae* comb. nov.

**Figure 31.**
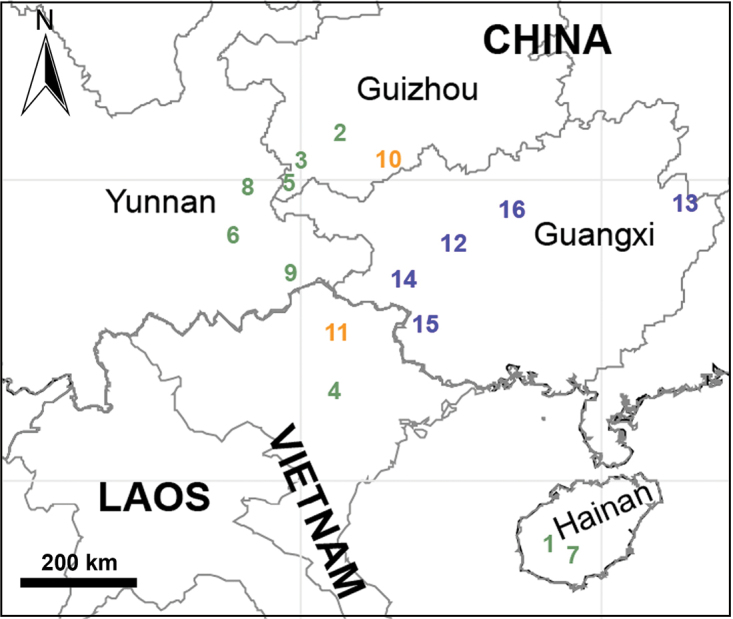
Distribution records of *Pinelema* spp., the *feilong*-group (green), the *pacchanensis*-group (orange), and the *podiensis*-group (blue): **1***P. bella* comb. nov. **2***P. circularis* comb. nov. **3***P. claviformis* comb. nov. **4***P. damtaoensis***5***P. feilong* comb. nov. **6***P. huobaensis***7***P. spina* comb. nov. **8***P. vesiculata* comb. nov. **9***P. yaosaensis***10***P. daguaiwan* sp. nov. **11***P. pacchanensis***12***P. bifida* comb. nov. **13***P. biyunensis* comb. nov. **14***P. podiensis***15***P. shiba* sp. nov.**16***P. zonaria* comb. nov.

**Figure 32.**
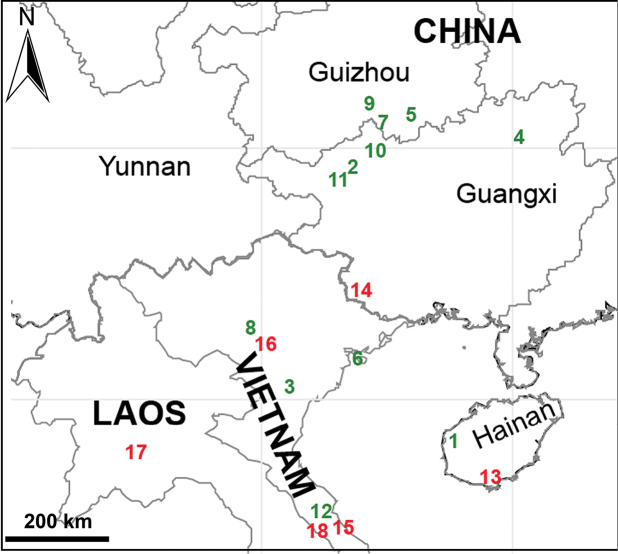
Distribution records of *Pinelema* spp., the *xiezi*-group (green) and species not attached to a species group (red): **1***P. breviseta* comb. nov. **2***P. conglobare* comb. nov. **3***P. cucphongensis* comb. nov. **4***P. cucurbitina* comb. nov. **5***P. dongbei* comb. nov. **6***P. exiloculata* comb. nov. **7***P. grandidens* comb. nov. **8***P. laensis***9***P. oculata* comb. nov. **10***P. pedati* comb. nov. **11***P. spinafemora* comb. nov. **12***P. xiezi***13***P. dengi* comb. nov. **14***P. mikrosphaira* comb. nov. **15***P. nuocnutensis***16***P. spirulata***17***P. tham* sp. nov. **18***P. zhenzhuang*.

**Figure 33.**
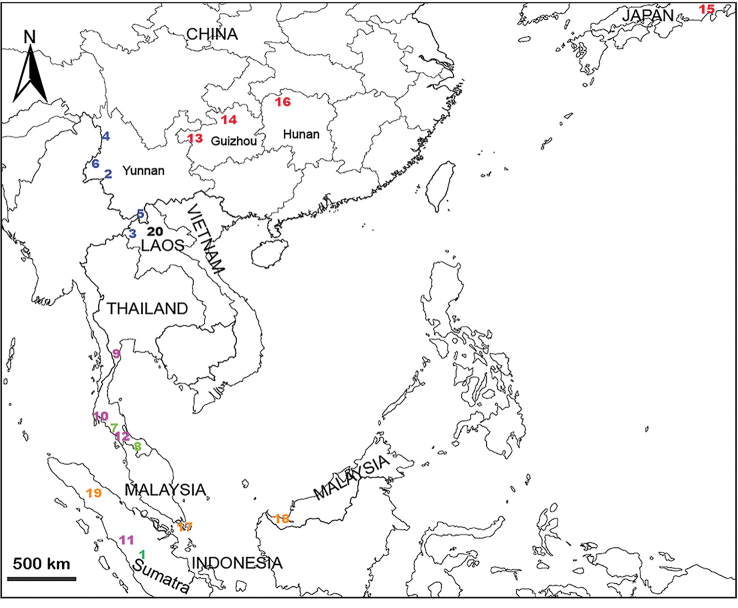
Distribution records of telemid species in East and Southeast Asia (except *Pinelema*). **1***Apneumonella jacobsoni***2***Mekonglema bailang* sp. nov. **3***M. kaorao* sp. nov. **4***M. walayaku* sp. nov. **5***M. xinpingi* comb. nov. **6***M. yan* sp. nov. **7***Siamlema changhai* sp. nov. **8***S. suea* sp. nov. **9***Sundalema acicularis* comb. nov. **10***S. anguina* comb. nov. **11***S. bonjol* sp. nov. **12***S. khaorakkiat* sp. nov. **13***Telema auricoma***14***T. guihua***15***T. nipponica***16***T. wunderlichi***17***Telemofila fabata* comb. nov. **18***T. malaysiaensis* comb. nov. **19***T. samosirensis***20***Zhuanlema peteri* sp. nov.

## Molecular phylogenetic analyses

### Sequence data and model selection

For 71 telemid taxa, a total of 71 and 67 sequences were successfully generated for H3 (331 base pairs, bp) and Wnt (330 bp), respectively. All sequences were submitted to GenBank (Suppl. material 1: Table S2). The best-fit Akaike information criterion (AIC) model of the concatenated BI dataset was SYM+I+G.

### Molecular phylogenetic results

**Figure 34.**
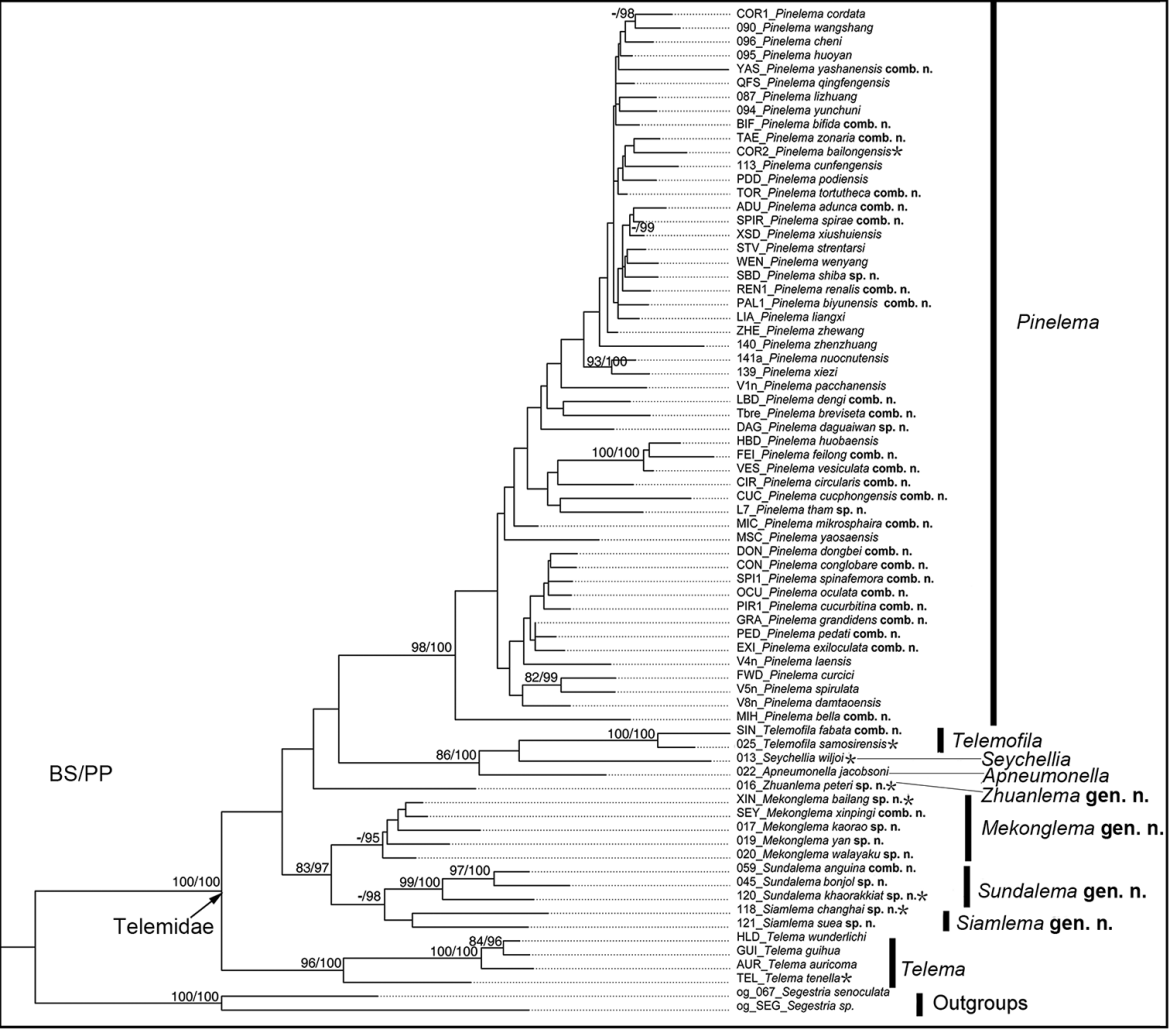
Maximum likelihood tree of Asian Telemidae. Each genus is indicated by black bar or solid line. BS < 70 and PP < 95 are not shown, taxa followed by an asterisk indicate the type species of the corresponding genus. BS: bootstrap support; PP: posterior possibility.

The topology of both the ML and BI trees are consistent at the genus level, branches to all tips are long, indicating distinct genetic divergence of each lineage (Fig. 34). Telemidae consists of two major clades: one of *Telema* and one of the other eight genera.

*Telema* includes four species (molecular data of *T. nipponica* was not acquired), and the remaining *Telema* species are clustered in *Pinelema*, *Sundalema* gen. nov., and *Telemofila* (new combinations in Fig. 34), supporting the hypothesis of previously incorrect generic placement of those species.

*Pinelema* is monophyletic with high support values (BS = 98 and PP = 100), although most interior nodes are not well supported (Fig. 34). *Telemofila* is robustly monophyletic (BS = 100 and PP = 100), and molecular data support the transfer of *Telemofila fabata* comb. nov. to this genus (Fig. 34). *Mekonglema xinpingi* comb. nov. did not cluster with the type species of *Seychellia*, so the hypothesis of the incorrect generic placement of *M. xinpingi* comb. nov. is supported (Fig. 34). The generic placement of *Apneumonella jacobsoni* is ambiguous because material of the type species *A. oculata* is lacking. The four new genera (*Mekonglema* gen. nov., *Siamlema* gen. nov., *Sundalema* gen. nov., and *Zhuanlema* gen. nov.) are monophyletic and (excluding *Siamlema* gen. nov.) strongly supported by both ML and BI (Fig. 34). This result is consistent with morphological delimitation (Fig. 34).

## Discussion

In this paper we constructed a molecular phylogeny of Telemidae from East and Southeast Asia for the first time, and our results have changed the taxonomic framework of Asian telemids. We revised the taxonomic status of these Asian telemids, introducing four new genera and 12 new species, and 31 species were transferred to other genera.

When researchers are delimiting genera of Telemidae, qualitative morphological characters should be considered. These characters include: for both sexes, the leg formula and the shape of tibial glands; for males, the presence or absence of a cymbial apophysis, the length ratio of the palpal femur/cymbium, and the accessory structures on the palpal tibia or bulb; for females, the shape of the receptacle and the presence/absence of membranous/sclerotized tubes within the receptacle. Quantitative morphological characters of reproductive organs should be considered when researchers are delimiting different species in the same genus. These characters include the ratio of the embolus/bulb length, the angle between the embolus and bulb, and the width ratio of the receptacle tip/neck, etc.

Despite the large genetic difference between *Telema* species in East Asia and *T. tenella* (e.g. the long branch between *T. tenella* and Asian *Telema* spp. in Fig. 34) this genus occupies a small morphospace. This is also supported by paleontological evidence, as the morphological characters of *T. moritzi* Wunderlich, 2004 (extinct species in Baltic amber, ca. 45 million years ago) are difficult to distinguish from extant *Telema* spp. (Wunderlich 2004). The occurrence of *Telema* spp. in the entire Palearctic is intriguing given their extremely weak dispersal ability and requires further investigation. It is possible that they dispersed from Europe to Asia (or *vice versa*) in ancient warm stage, then many species became extinct when the climate cooled. Only a few species survived in refuges like southern Europe and the tropical and subtropical areas of Asia, and this has resulted in the current fragmented distribution.

## Supplementary Material

XML Treatment for Pinelema

XML Treatment for adunca

XML Treatment for Pinelema adunca

XML Treatment for Pinelema qingfengensis

XML Treatment for Pinelema renalis

XML Treatment for Pinelema tortutheca

XML Treatment for Pinelema yashanensis

XML Treatment for bailongensis

XML Treatment for Pinelema bailongensis

XML Treatment for Pinelema curcici

XML Treatment for cunfengensis

XML Treatment for Pinelema cunfengensis

XML Treatment for Pinelema spirae

XML Treatment for feilong

XML Treatment for Pinelema bella

XML Treatment for Pinelema circularis

XML Treatment for Pinelema claviformis

XML Treatment for Pinelema damtaoensis

XML Treatment for Pinelema feilong

XML Treatment for Pinelema huobaensis

XML Treatment for Pinelema spina

XML Treatment for Pinelema vesiculata

XML Treatment for Pinelema yaosaensis

XML Treatment for pacchanensis

XML Treatment for Pinelema daguaiwan

XML Treatment for Pinelema pacchanensis

XML Treatment for podiensis

XML Treatment for Pinelema bifida

XML Treatment for Pinelema biyunensis

XML Treatment for Pinelema podiensis

XML Treatment for Pinelema shiba

XML Treatment for Pinelema zonaria

XML Treatment for xiezi

XML Treatment for Pinelema breviseta

XML Treatment for Pinelema conglobare

XML Treatment for Pinelema cucphongensis

XML Treatment for Pinelema cucurbitina

XML Treatment for Pinelema dongbei

XML Treatment for Pinelema exiloculata

XML Treatment for Pinelema grandidens

XML Treatment for Pinelema laensis

XML Treatment for Pinelema oculata

XML Treatment for Pinelema pedati

XML Treatment for Pinelema spinafemora

XML Treatment for Pinelema xiezi

XML Treatment for Pinelema dengi

XML Treatment for Pinelema mikrosphaira

XML Treatment for Pinelema nuocnutensis

XML Treatment for Pinelema spirulata

XML Treatment for Pinelema tham

XML Treatment for Pinelema zhenzhuang

XML Treatment for Apneumonella

XML Treatment for Apneumonella jacobsoni

XML Treatment for Mekonglema

XML Treatment for Mekonglema bailang

XML Treatment for Mekonglema kaorao

XML Treatment for Mekonglema walayaku

XML Treatment for Mekonglema xinpingi

XML Treatment for Mekonglema yan

XML Treatment for Siamlema

XML Treatment for Siamlema changhai

XML Treatment for Siamlema suea

XML Treatment for Sundalema

XML Treatment for Sundalema bonjol

XML Treatment for Sundalema khaorakkiat

XML Treatment for Sundalema acicularis

XML Treatment for Sundalema anguina

XML Treatment for Telema

XML Treatment for Telema auricoma

XML Treatment for Telema guihua

XML Treatment for Telema nipponica

XML Treatment for Telema wunderlichi

XML Treatment for Telemofila

XML Treatment for Telemofila fabata

XML Treatment for Telemofila malaysiaensis

XML Treatment for Telemofila samosirensis

XML Treatment for Zhuanlema

XML Treatment for Zhuanlema peteri

## Footnotes

Females of Pinelema and Apneumonella are indistinguishable.

Mekonglema gen. nov. part I includes M. kaorao sp. nov., M. walayaku sp. nov., and M. xinpingi comb. nov.

Females of Mekonglema gen. nov. part II (M. bailang sp. nov. and M. yan sp. nov.) and Siamlema gen. nov. part II (S. changhai sp. nov.) are indistinguishable.

## Supplementary materials

Supplementary material 1 Taxonomic revision on Telemidae (Arachnida, Araneae) from East and Southeast Asia Data type Primer/specimen/DNA sequence Explanation note **Table S1.** The locus, primer pairs, and PCR protocols used in this study (Nested PCR, the product of round 1 was used as the template for round 2). **Table S2.** List of voucher information and GenBank accession numbers (na, indicates the molecular data is not acquired). https://binary.pensoft.net/file/411182This dataset is made available under the Open Database License (http://opendatacommons.org/licenses/odbl/1.0/). The Open Database License (ODbL) is a license agreement intended to allow users to freely share, modify, and use this Dataset while maintaining this same freedom for others, provided that the original source and author(s) are credited.Huifeng Zhao, Shuqiang Li, Aibing Zhang
