# Supplementary material for: Taxonomic revision of Telemidae (Arachnida, Araneae) from East and Southeast Asia
Source: Zookeys. 2020 May 18;933:15–93. doi: 10.3897/zookeys.933.38653 (PMC7248132; doi:10.3897/zookeys.933.38653)
Supplement: Supplementary material 1 — Taxonomic revision on Telemidae (Arachnida, Araneae) from East and Southeast Asia [file zookeys-933-015-s001.doc]

Table S1. The locus, primer pairs, and PCR protocols used in this study (Nested PCR, the product of round 1 was used as the template for round 2).

| Locus |  | Primer | Sequence (forward and reverse) 5’→3’ | Nested PCR | Annealing temperature and cycle number | References |
| --- | --- | --- | --- | --- | --- | --- |
| H3 | F | H3af | ATGGCTCGTACCAAGCAGACVGC | No | 51℃ (30) | Colgan et al. 1998 |
|  | R | H3ar | ATATCCTTRGGCATRATRGTGAC |  |  |  |
| Wnt | F | SPWGF1 | GYAAATGCCAYGGWATGTCMGG | Round 1 | 48℃ (30) | Blackledge et al. 2009 |
|  | R | SPWGR1 | ACTTGRCAACACCARTGAAAWG |  |  |  |
|  | F | WNT2F | CNGTTCAAACTTGYTGGATG | Round 2 | 51℃ (30) | Li et al. 2020 |
|  | R | WNT2R | CAGTGRAATGTRCARTTG |  |  |  |

**References cited in table S1**

Blackledge TA, Scharff N, Coddington JA, Szüts T, Wenzel JW, Hayashi CY, Agnarsson I (2009) Reconstructing web evolution and spider diversification in the molecular era. Proceedings of the National Academy of Sciences of the United States of America 106: 5229–5234. https://doi.org/10.1073/pnas.0901377106

Colgan DJ, McLauchlan A, Wilson GDF, Livingston SP, Edgecombe GD, Macaranas J, Cassis G, Gray MR (1998) Histone H3 and U2 snRNA DNA sequences and arthropod molecular evolution. Australian Journal of Zoology 46: 419. https://doi.org/10.1071/zo98048

Li F, Shao L, Li S (2020) Tropical niche conservatism explains the Eocene migration from India to Southeast Asia in ochyroceratid spiders. Systematic Biology, published online. https:// doi.org/10.1093/sysbio/syaa006

Table S2. List of voucher information and GenBank accession numbers (na, indicates the molecular data is not acquired)

| Voucher_ID | Locality | Species | H3 | Wnt |
| --- | --- | --- | --- | --- |
| 013 | Mahe, Seychelles | *Seychellia wiljoi* | MN706453 | MN706383 |
| 016 | Luang Prabang, Laos | *Zhuanlema peteri* sp. nov. | MN706454 | MN706384 |
| 017 | Luang Prabang, Laos | *Mekonglema kaorao* sp. nov. | MN706452 | MN706385 |
| 019 | Yunnan, China | *Mekonglema yan* sp. nov. | MN706455 | MN706386 |
| 020 | Yunnan, China | *Mekonglema walayaku* sp. nov. | MN706456 | MN706387 |
| 022 | Sumatra, Indonesia | *Apneumonella jacobsoni* | MN706468 | na |
| 025 | Sumatra, Indonesia | *Telemofila samosirensis* | MN706457 | MN706388 |
| 045 | Sumatra, Indonesia | *Sundalema bonjol* sp. nov. | MN706458 | MN706389 |
| 059 | Krabi, Thailand | *Sundalema anguina* | MN706459 | MN706390 |
| 087 | Guangxi, China | *Pinelema lizhuang* | MN706460 | MN706391 |
| 090 | Guangxi, China | *Pinelema wangshang* | MN706461 | MN706392 |
| 094 | Guangxi, China | *Pinelema yunchuni* | MN706462 | MN706393 |
| 095 | Guangxi, China | *Pinelema huoyan* | MN706463 | MN706394 |
| 096 | Guangxi, China | *Pimeloma cheni* | MN706464 | MN706395 |
| 113 | Guangxi, China | *Pinelema cunfengensis* | MN706465 | MN706396 |
| 118 | Trang, Thailand | *Siamlema changhai* sp. nov. | MN706466 | MN706397 |
| 120 | Song Khla, Thailand | *Sundalema khaorakkiat* sp. nov. | MN706467 | MN706398 |
| 121 | Yala, Thailand | *Siamlema suea* sp. nov. | MN706469 | MN706399 |
| 139 | Quang Binh, Vietnam | *Pinelema xiezi* | MN706471 | MN706400 |
| 140 | Quang Binh, Vietnam | *Pinelema zhengzhuang* | MN706472 | na |
| 141a | Quang Binh, Vietnam | *Pinelema nuocnutensis* | MN706473 | MN706401 |
| ADU | Guangxi, China | *Pinelema adunca* | MN706474 | MN706402 |
| AUR | Guizhou, Yunnan, China | *Telema auricoma* | MN706484 | MN706403 |
| BIF | Guangxi, China | *Pinelema bifida* | MN706470 | MN706404 |
| CIR | Guizhou, China | *Pinelema circularis* | MN706477 | MN706407 |
| CON | Guangxi, China | *Pinelema conglobare* | MN706478 | MN706408 |
| COR1 | Guangxi, China | *Pinelema cordata* | MN706475 | MN706405 |
| COR2 | Guangxi, China | *Pinelema bailongensis* | MN706476 | MN706406 |
| CUC | Cuc Phuong National Park, Vietnam | *Pinelema cucphongensis* | MN706479 | MN706409 |
| DAG | Guizhou, China | *Pinelema daguaiwan* sp. nov. | MN706480 | MN706410 |
| DON | Guizhou, China | *Pinelema dongbei* | MN706481 | MN706411 |
| EXI | Cat Ba National Park, Vietnam | *Pinelema exiloculata* | MN706482 | MN706412 |
| FEI | Guizhou, China | *Pinelema feilong* | MN706483 | MN706413 |
| FWD | Yunnan,China | *Pinelema curcici* | MN706485 | MN706414 |

Table S2. Continued.

| GRA | Guizhou, China | *Pinelema grandidens* | MN706487 | na |
| --- | --- | --- | --- | --- |
| GUI | Guizhou, China | *Telema guihua* | MN706488 | MN706415 |
| HBD | Yunnan,China | *Pinelema huobaensis* | MN706489 | MN706416 |
| HLD | Hunan, China | *Telema wunderlichi* | MN706490 | na |
| L7 | Vientiane, Laos | *Pinelema tham* sp. nov. | MN706486 | MN706419 |
| LBD | Hainan, China | *Pinelema dengi* | MN706491 | MN706417 |
| LIA | Guizhou, China | *Pinelema liangxi* | MN706492 | MN706418 |
| MIC | Guangxi, China | *Pinelema mikrosphaira* | MN706493 | MN706420 |
| MIH | Hainan, China | *Pinelema bella* | MN706494 | MN706421 |
| MSC | Yunnan,China | *Pinelema yaosaensis* | MN706495 | MN706422 |
| OCU | Guizhou, China | *Pinelema oculata* | MN706496 | MN706423 |
| og_067 | Xinjiang, China | *Segestria senoculata* | MN706523 | MN706450 |
| og_SEG | Xinjiang, China | *Segestria sp*. | MN706524 | MN706451 |
| PAL1 | Guangxi, China | *Pinelema biyunensis* | MN706497 | MN706424 |
| PDD | Guangxi, China | *Pinelema podiensis* | MN706498 | MN706425 |
| PED | Guangxi, China | *Pinelema pedati* | MN706499 | MN706426 |
| PIR1 | Guangxi, China | *Pinelema cucurbitina* | MN706500 | MN706427 |
| QFS | Guangxi, China | *Pinelema qingfengensis* | MN706501 | MN706428 |
| REN1 | Guangxi, China | *Pinelema renalis* | MN706502 | MN706429 |
| SBD | Guangxi, China | *Pinelema shiba* | MN706503 | MN706431 |
| SEY | Yunnan,China | *Mekonglema xinpingi* comb. nov. | MN706504 | MN706432 |
| SIN | Singapore | *Telemofila fabata* | MN706522 | MN706430 |
| SPI1 | Guangxi, China | *Pinelema spinafemora* | MN706505 | MN706433 |
| SPIR | Guangxi, China | *Pinelema spirae* | MN706506 | MN706434 |
| STV | Guangxi, China | *Pinelema strentarsi* | MN706507 | MN706435 |
| TAE | Guangxi, China | *Pinelema zonaria* | MN706508 | MN706436 |
| Tbre | Hainan, China | *Pinelema breviseta* | MN706509 | MN706437 |
| TEL | France | *Telema tenella* | MN706510 | MN706438 |
| TOR | Hainan, China | *Pinelema tortutheca* | MN706511 | MN706439 |
| V1n | Bac Kan, Vietnam | *Pinelema pacchanensis* | MN706512 | MN706440 |
| V4n | Phu Tho, Vietnam | *Pinelema laensis* | MN706513 | MN706441 |
| V5n | Phu Tho, Vietnam | *Pinelema spirulata* | MN706514 | MN706442 |
| V8n | Vinh Phuc, Vietnam | *Pinelema damtaoensis* | MN706515 | MN706443 |
| VES | Yunnan,China | *Pinelema vesiculata* | MN706516 | MN706444 |
| WEN | Guangxi, China | *Pinelema wenyang* | MN706517 | MN706445 |
| XIN | Yunnan,China | *Mekonglema bailing* sp. nov. | MN706518 | MN706446 |
| XSD | Guangxi, China | *Pinelema xiushuiensis* | MN706519 | MN706447 |
| YAS | Guangxi, China | *Pinelema yashanensis* | MN706520 | MN706448 |
| ZHE | Guizhou, China | *Pinelema zhewang* | MN706521 | MN706449 |
